# Developing Quorum Sensing‐Based Collaborative Dynamic Control System in *Halomonas* TD01

**DOI:** 10.1002/advs.202408083

**Published:** 2025-03-16

**Authors:** Yi‐Na Lin, Yu‐Xi Li, Ye Zheng, Yi‐Hao Deng, Kai‐Xuan Liu, Yue Gan, Hao Li, Jun Wang, Jia‐Wen Peng, Rui‐Zhe Deng, Huai‐Ming Wang, Hui Wang, Jian‐Wen Ye

**Affiliations:** ^1^ School of Biology and Biological Engineering South China University of Technology Guangzhou 510006 China; ^2^ Department of General Surgery (Colorectal Surgery) The Sixth Affiliated Hospital Sun Yat‐sen University Guangzhou 510655 China; ^3^ Guangdong Provincial Key Laboratory of Colorectal and Pelvic Floor Diseases The Sixth Affiliated Hospital Sun Yat‐sen University Guangzhou 510655 China; ^4^ Biomedical Innovation Center The Sixth Affiliated Hospital Sun Yat‐sen University Guangzhou 510655 China

**Keywords:** dynamic control, quorum sensing, high cell density induction, *Halomonas*, cell‐cell communication, metabolic engineering

## Abstract

Dynamic control exhibits increasing significance in microbial cell factory engineering by precisely manipulating gene expression over time and levels. However, the practical uses of most dynamic control tools still remain challenging because of poor scale‐up robustness, especially for non‐model chassis. Herein, a quorum sensing (QS)‐based collaborative dynamic control system is constructed in *Halomonas* TD by regrouping two orthogonal quorum‐sensing modules into two cell types, namely cell‐A harboring *cinR‐luxI* and cell‐B harboring *luxR‐cinI* together with sfGFP driven by P_cin_ and P_lux_ promoters, respectively. Effective gene expression control with over 15‐time dynamic foldchange is achieved by mixing cells A and B at different ratios and time points in a lab‐scale fed‐batch study. Besides, dynamic inhibitory and amplified control is further developed by cascading CRISPRi/dCas9 system and MmP1 RNA polymerase, respectively, yielding up to 80% repression efficiency and 30‐time amplification foldchange under high cell density fermentation. Moreover, 500 mg L^−1^ indigo and 4.7 g L^−1^ superoxide dismutase (SOD) are obtained by engineered *Halomonas* using QS‐based control tools in the fed‐batch study, showing 1.5‐ and 1.0‐fold higher, respectively, than the yields by recombinants induced by IPTG. This study exemplifies a standardized and streamlined inducer‐free dynamic control pattern for metabolic engineering with promising robustness in scale‐up fermentation contexts.

## Graphical abstract

A standardized and streamlined inducer‐free quorum sensing (QS)‐based collaborative dynamic control pattern is developed for sufficient gene expression control in *Halomonas* TD by mixing two engineered cells, cells A and B. Proof‐of‐concept studies are designed to test the dynamic control performance in mixed cultures in fed‐batch conditions. Optimized QS‐based control systems are applied to achieve high‐yield production of indigo and superoxide dismutase (SOD).

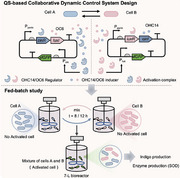

## 1 Introduction

Metabolic engineering of microbes powered by synthetic biology tools^[^ ^1^, ^2^, ^3^, ^4^, ^5^ ^]^ aims to rewire cellular metabolism toward different value‐added bioproductions for industrial biomanufacturing purposes.^[^ ^6^, ^7^ ^]^ In addition to pathway optimization, different genetic circuits have been developed to achieve precise and complicated gene expression control for various microbial engineering purposes.^[^ ^4^, ^8^, ^9^ ^]^ For example, engineered microbes harboring tailor‐made biosensors in response to diverse input signals, such as chemical molecules,^[^ ^2^, ^10^ ^]^ temperature,^[^ ^11^ ^]^ light^[^ ^12^, ^13^, ^14^, ^15^ ^]^ intermediate metabolites^[^ ^16^, ^17^ ^]^ and cell population,^[^ ^18^, ^19^, ^20^ ^]^ display proven feasibility in multifunction pathway regulation to address the trade‐off difficulties of production synthesis and cell growth during batch and/or fed‐batch fermentation process.^[^ ^4^, ^21^, ^22^, ^23^ ^]^ However, challenges still remain for industrial scale‐up uses, such as the difficulties of light‐control discussed by Joaquin Gutierrez Mena et al.^[^ ^24^ ^]^ and Sylvain Pouzet et al.^[^ ^25^ ^]^

Recently, dynamic regulation based on cell‐cell communication devices has become an attractive strategy for different purposes, such as bioproduction optimization,^[^ ^20^ ^]^ multicellular behavior control,^[^ ^26^ ^]^ layer‐cascaded logic gate engineering,^[^ ^10^, ^27^ ^]^ edge detection and computing,^[^ ^28^ ^]^ and so on.^[^ ^29^ ^]^ In particular, quorum sensing (QS)‐based control depending on a growing cell population with accumulated N‐acyl homoserine lactones (AHLs) as input signals, allowing free diffusion across cell membranes, has been extensively studied for diverse application purposes.^[^ ^30^ ^]^ Various QS modules, including lux,^[^ ^3^ ^]^ cin,^[^ ^3^ ^]^ esa,^[^ ^31^ ^]^ tra^[^ ^32^ ^]^ and so on,^[^ ^33^, ^34^ ^]^ have been thus mined and studied. The well‐studied QS device from *Vibrio fisheri*,^[^ ^35^ ^]^ namely lux system consisting of LuxR regulator, AHL (OC6) synthase encoded by *luxI* gene, and QS‐controlled promoter P_lux_, has been used for metabolic flux control in different recombinant chassis like *Escherichia coli* (*E. coli*),^[^ ^36^ ^]^ *Bacillus subtilis* ^[^ ^37^ ^]^ and so on. Moreover, different QS‐based genetic circuits of mono‐, bi‐ and multi‐function control behaviors in both single cell population and microbial consortia levels have been developed to achieve versatile dynamic modulation of protein resource re‐allocation,^[^ ^19^, ^33^ ^]^ trade‐off between cell growth and biosynthesis of inositol,^[^ ^20^ ^]^ glucaric acid^[^ ^38^ ^]^ etc., demonstrating great potential in microbial cell factory engineering without extract cost for supplying input signals like IPTG, arabinose, etc.

However, because of the low response threshold of cell density, which would lead to unexpected early execution of control functions during the scale‐up seed preparation process, further industrial uses currently still remain challenged. In addition, recent works focusing on QS‐based cell‐cell communication have established different microbial consortia of diversified characterizations, such as genetic oscillation,^[^ ^39^ ^]^ customized pattern formation,^[^ ^40^ ^]^ co‐repressive circuit,^[^ ^41^, ^42^ ^]^ cell state control^[^ ^43^ ^]^ and so on. Therefore, the increasing proof‐of‐concept studies of QS‐based cell‐cell communication showed promising potentials for biomimetic microbial consortia design over temporal and spatial levels. Nevertheless, engineering cell‐cell communication allowing precise dynamic gene expression and/or repression control for large‐scale industrial uses is still difficult to achieve, such as interactive patterns and the ratio of recombinant cells under strict sterilization conditions.

In this study, we have constructed a cell‐cell collaborative dynamic control system based on the combinatory design of Cin‐ (CinR/CinI) and Lux‐ (LuxR/LuxI) type QS modules in *Halomonas* TD, which is a well‐studied halophilic chassis of predominant polyhydroxyalkanoates (PHA) synthesis capability allowing open and unsterile fermentation.^[^ ^44^, ^45^, ^46^ ^]^ In previous reports, QS modules like lux and cin have been successfully constructed in *Halomonas* TD, however, only used as inducible systems for static pathway fine‐tuning instead of dynamic control.^[^ ^3^, ^47^ ^]^ Here, dynamic expression and repression control of genes of interest over time and level has been achieved at the desired time point throughout batch and fed‐batch fermentation process, yielding maximal over 1000‐fold dynamic range for gene activation and 80% gene repression efficiency by cascading the uses of MmP1 RNA polymerase and dCas9, respectively. Enhanced production synthesis performance of indigo, poly(3‐hydroxybutyrate‐*co*‐3‐hydroxyvalerate) (PHBV), and superoxide dismutase (SOD) were achieved by recombinant *Halomonas* TD using QS‐based collaborative dynamic control. Therefore, our results demonstrated a convenient and effective “just in time” dynamic control paradigm by recombinant *Halomonas* TD during high cell density growth at varied cultural scales under open and unsterile conditions.

## 2 Results

### 2.1 Constructing QS‐Based Collaborative Dynamic Control System

**Figure 1:**
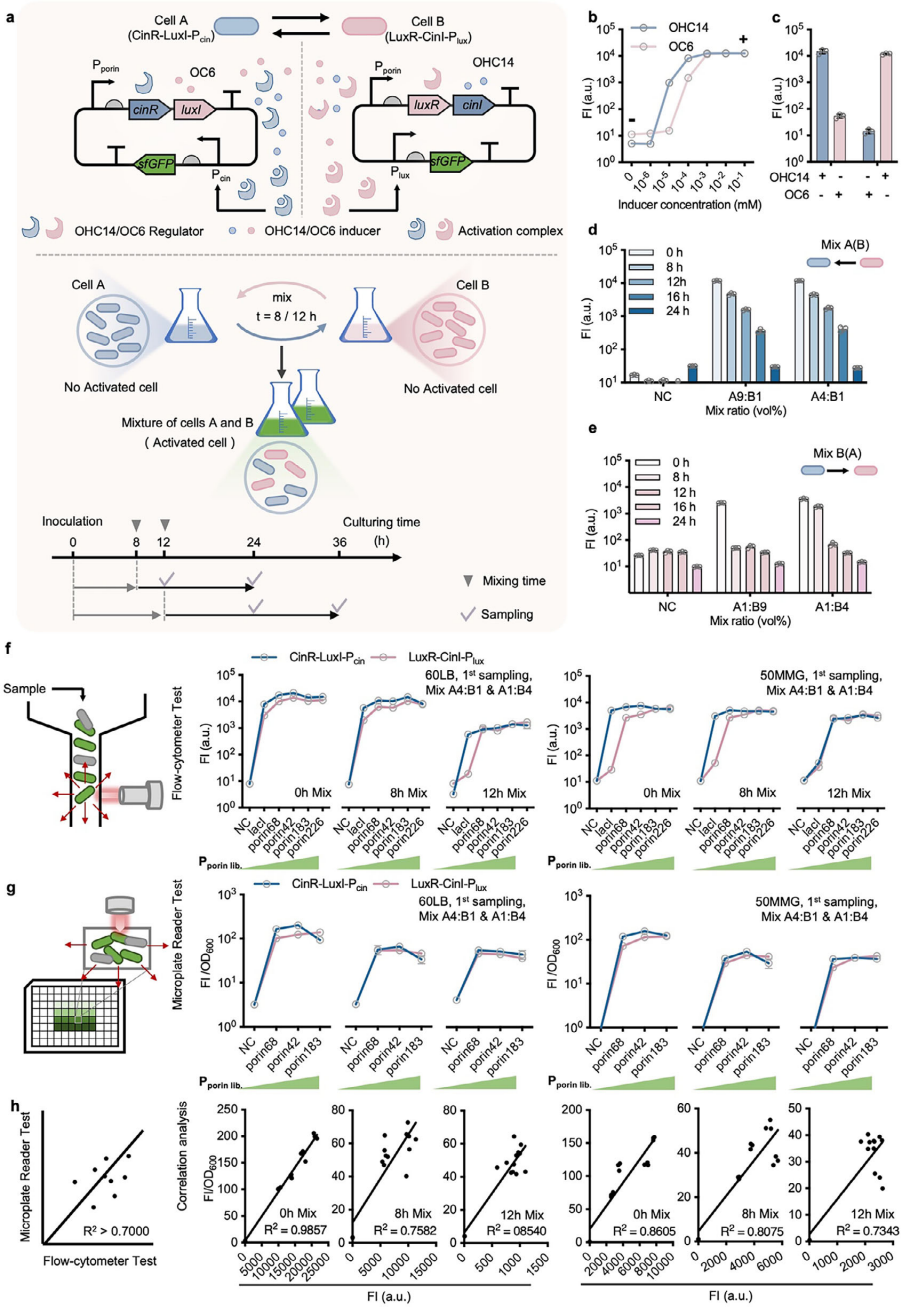
Design and characterization of QS‐based collaborative dynamic control system by recombinant *Halomonas* TD. a) A circuit diagram of a QS‐based collaborative dynamic control system was designed for gene expression control in *Halomonas* TD, forming cells A and B. Characterization workflow was also designed to test the dynamic control performance of cell‐A and ‐B mixed culture. b) Dose‐response curves of two QS‐based collaborative circuits in cells A and B were characterized independently in 60LB by supplementing different concentrations of OHC14 (cell A) and OC6 (cell B), respectively. Fluorescent intensity of cells A and B with *sf*GFP expression controlled by P_cin_ and P_lux_, respectively, was analyzed by flow cytometry. c) Orthogonal test across cells A and B by supplementing OHC14 and OC6, respectively. “+” and “–” indicate 10^−2^ mM and 0 mM supplementation of both OHC14 and OC6. d. e) Prototyping test of QS‐based collaborative dynamic control system in *Halomonas* TD by mixing cells A and B with different volume ratios (9:1, A9:B1 in d and A1:B9 in e; 4:1, A4:B1 in d and A1:B4 in e) after 0, 8, 12, 16 and 24 h independent pre‐culture of cells A and B in 60LB. f) Promoter engineering was employed to optimize the expression level of *cinR‐luxI* and *luxR‐cinI* for enhanced dynamic induction performance. Promoter mutants of different strengths including P_porin68_, P_porin42_, P_porin183,_ and P_porin226_ were constructed and tested in contrast to the original one (P_lacI_). FI of mixed culturing cells grown in 60LB and 50MMG, respectively, were analyzed by flow cytometer. g) Constructs containing optimized *cinR*‐*luxI* and *luxR*‐*cinI* modules driven by P_porin68_, P_porin42,_ and P_porin183_, respectively, were selected for further testing by microplate reader compared to the negative control group (NC). h) Correlation analysis of mixed cultural expression levels of sfGFP from part f (*x‐axis*) and g (*y‐axis*). Data point plots with R^2^ > 0.7 demonstrated a high correlation ship of FI measurement between flow‐cytometer analysis at the single‐cell level and microplate reader in the cell‐population level. NC, wild‐type TD. FI, Fluorescent intensity in arbitrary unit (a.u.). FI/OD_600_, normalized fluorescence by dividing OD_600_. Cell cultures were obtained and analyzed via flow‐cytometer (100‐time dilution, 1st sampling) and/or microplate reader (diluted to 0.2–0.8 of OD_600_, 1st sampling). Data points in b to g are shown as mean ± SD of three replicates.

Two orthogonal QS‐regulators encoded by *luxR* and *cinR*, and QS‐signal molecule synthases encoded by *luxI* and *cinI*, were grouped into two modules, *cinR‐luxI* and *luxR‐cinI* driven by P_lacI_ promoter, and then constructed in *Halomonas* TD independently, together with sfGFP reporter units controlled by P_cin_ and P_lux_ promoters, respectively, forming two types of cells, cell A (CinR‐LuxI‐P_cin_) and cell B (LuxR‐CinI‐P_lux_) (**Figure** **1**a). Cells A and B with independent growth can synthesize AHL signal molecules, OHC14 and OC6, respectively, which could diffuse freely across the cell membrane. Intracellular regulators CinR and LuxR with specific binding force to OHC14 and OC6, respectively, are able to form complexes CinR‐OHC14 and LuxR‐OC6 only after mixing the cultures of cells A and B, which leads to effective induction of target genes (e.g., *sf*GFP) driven by P_cin_ and P_lux_, namely QS‐based collaborative dynamic control system. Thus, the “ON” and “OFF” stages of target gene expression over time and level can be tightly controlled by mixing two independent cell cultures with a designed ratio. The mixing time point can be freely adjusted, such as 8 and 12 h growth after inoculation under different cell densities, to achieve desired dynamic control performance.

Before assessing the QS‐based dynamic control performance, dose‐response curves of two constructs, CinR‐LuxI‐P_cin_ and LuxR‐CinI‐P_lux_, contained by recombinant cells A and B were first characterized by supplementing different concentrations of OHC14 and OC6 as inducers, respectively (Figure 1b). The fluorescent intensity (FI) of cells A and B was measured by flow cytometer with a dynamic range reaching up to 2489‐ and 1109‐fold, respectively, between “ON” and “OFF” stages. Meanwhile, the orthogonality of these two constructs was studied by cells A and B, respectively, grown in 60LB medium supplemented with 10^−2^ and 0 mM OHC14 and OC6, which showed negligible crosstalk effects of CinR versus OC6 and LuxR versus OHC14. These results indicate promising potentials for ON‐stage expression and OFF‐stage leakiness control of cells A and B under mixed and independent growth, respectively (Figure 1c). Subsequently, mixed culturing of cells A and B at different ratios, from 1:99 to 1:1, and time points of varied cell density, including 0, 8, 12, 16, and 24 h growth after inoculation, were performed to study the dynamic control performance by recording FI value of recombinant cells. Interestingly, 0.5 vol% of cell B initially mixed with 99.5 vol% cell A (A199:B1) as inoculum (mixed at 0 h) for 12 h growth was able to achieve effective expression of sfGFP with FI value greater than 10^3^. And saturated induction level of sfGFP could be obtained at the mixed ratio of A9:B1 and A4:B1 (Figure 1d; Figure S1a, Supporting Information). Nevertheless, only when the mixed ratio of cells A and B reached 1:9 (A1:B9) or higher can lead to significant expression of sfGFP with FI value approximately reaching from 2000 to 5000 (Figure 1e; Figure S1b, Supporting Information), which was still much lower than that directly induced by 10^−3^ mM OC6, reaching over 10^4^ (Figure 1b). Besides, an increased mixed ratio of cell A into cell B culture (from A1:B9 to A1:B4) can significantly improve the FI level of B(A) group (Figure 1e). Meanwhile, 10^−3^ mM OC6 and OCH14 were additionally supplemented into A1:B9 and A9:B1 mixed culture, respectively, with an aim to achieve improved induction level of sfGFP (Figure S1c, Supporting Information). Results showed that a significant improvement of FI can be observed in A1:B9 group while a slight improvement was obtained in A9:B1 group. Therefore, increasing the mixing ratio of two cell types and engineering the synthesis of QS signal molecules are both effective strategies to improve the dynamic induction output. Moreover, longer growth of cells A and B before mixing, especially for pre‐cultured cells entering stationary phase after over 16 h growth, affects the dynamic control performance of both mixed cultural groups, A(B) and B(A), with dramatic decreased expression level of sfGFP (Figure 1d,e; Figure S2, Supporting Information).

To optimize the induction output of the QS‐based collaborative dynamic control system, *porin* promoter mutants of varied strength, including P_porin68_, P_porin42_, P_porin183_ and P_porin226_^[^ ^48^ ^]^ was constructed to achieve enhanced expression of QS‐based control panels, *cinR*‐*luxI*, and *luxR*‐*cinI*, in contrast to the prototyping design driven by weak P_lacI_ promoter, as well as wild type strain used as negative control (NC). Obviously, the dynamic induction levels of sfGFP by recombinant cells mixed (A4:B1 & A1:B4) at different time points grown in 60LB and 50MMG media, respectively, were improved significantly, especially for cell cultures grown in 50MMG medium or mixed at late log‐phase (12 h mix) (Figure 1f; Figure S2, Supporting Information). In particular, the FI of cell cultures sampled after 24 h mixed growth (2nd sampling, Figure S3, Supporting Information) manifested a consistent expression level of sfGFP in contrast to that sampled at 12 h (1st sampling, Figure 1f), which allows long‐term intracellular enzyme maintenance once induced. Besides, the FI value of mixed culturing cells from 60LB groups plotted against 50MMG ones displayed high linear correlation‐ship with Pearson coefficient (*R* ^2^) reaching over 0.7 (Figure S4, Supporting Information), demonstrating robust gene expression control performance from nitrogen‐limited to nitrogen‐enriched conditions. Moreover, FI of mixed culturing cells from Figure 1f, including P_porin68_, P_porin42_, P_porin183,_ and NC groups, was also measured by microplate reader simultaneously to analyze the correlation‐ship of gene expression performance between single cell level and cell population level (Figure 1g; Figures S5 and S6, Supporting Information), which is important for scale‐up robustness of industrial purpose. Similar dynamic control patterns of mixed growing cells were observed across different recombinant constructs, mixing time points, and growth media, showing high linear correlations with *R* ^2^ varied from 0.72 to 0.98 (Figure 1h; Figure S7, Supporting Information), which was close to the constitutive *porin* promoter mutants (P_porin68_, P_porin58_, P_porin183_, P_porin140_, and P_porin141_) analyzed in the same way using sfGFP as a reporter (*R* ^2^ = 0.94–0.99) (Figure S8, Supporting Information).^[^ ^48^ ^]^ Therefore, optimized performance of QS‐based dynamic control systems can be achieved by modulating the expression level of QS‐based control panels containing regulators and signal molecule synthases. Notably, stronger expression of QS‐based control panels driven by P_porin183_ and P_porin226_ only did not show effective improvement on sfGFP expression (Figure 1f,g).

### 2.2 Prototyping Test and Applications of QS‐Based Collaborative Dynamic Control System

**Figure 2:**
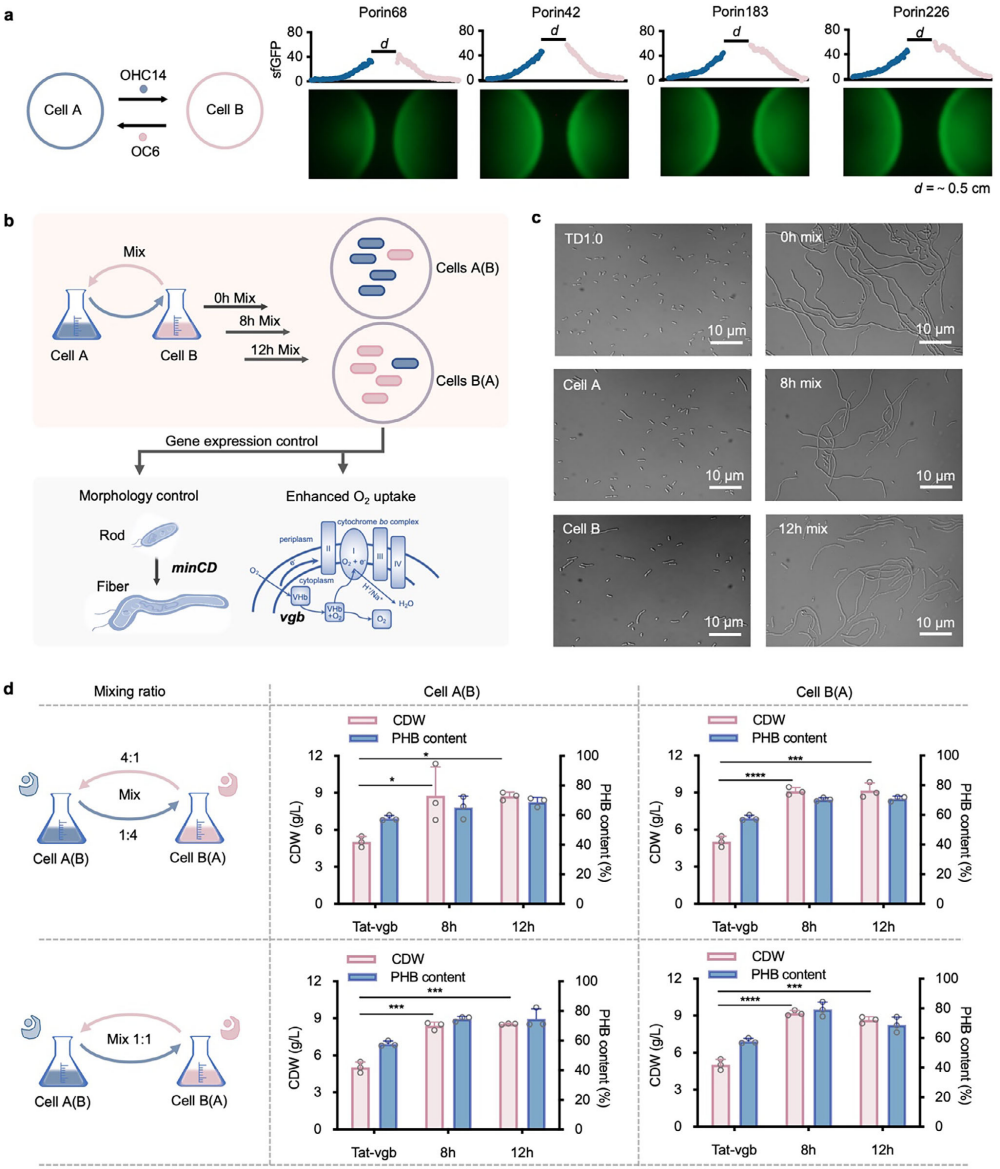
Prototyping test and applications of QS‐based collaborative dynamic control system in *Halomonas* TD. a) Interactive pattern formation from two independent bacterial lawns with different cell‐cell communication capabilities (*cinR‐luxI* and *luxR‐cinI* modules driven by P_porin68_, P_porin42_, P_porin183,_ and P_porin226_, respectively) were recorded after 24 h incubation on agar plates. FI of different bacterial lawns was quantified by software ImageJ. Distance (*d*) between two bacteria lawns was set at 0.5 cm. b) Schematic design for dynamic control of cell morphology and oxygen uptake capability mediated by controlling the expression of *minCD* and *vgb* clusters, respectively. c) Confocal imaging of mixed cultural cells A and B harboring *minCD* cluster controlled by P_cin_ and P_lux_, respectively. Cells A and B were mixed at 1:1 (vol%) after 0, 8, and 12 h pre‐culture independently (right row). Strains of wild‐type TD, cells A and B were independently cultured as control groups (left row). d) Cell growth and PHB accumulation by recombinant *Halomonas* TD with induced *vgb* expression. Cell A (*vgb* driven by P_cin_) and B (*vgb* driven by P_lux_) were mixed at 4:1 (upper panel) and 1:1 (bottom panel) ratios, respectively, after 8 and 12 h pre‐culture independently. Histograms in the middle and right rows indicate two independent mixed groups of each mixed ratio. Constitutive expression of Tat‐*vgb* (Tat‐*vgb*) was used as a control based on a previous study. Data points in d are shown as mean ± SD of three replicates. *p* value: n.s., not significant; *p < 0.0332; **p < 0.0021; ***p < 0.0002; ****p < 0.0001.

To further assess the variance of cell‐cell communication capability by cells A and B containing different QS‐based control panels under the control of P_porin68_, P_porin42_, P_porin183,_ and P_porin226_ promoters, visualized interacting patterns were studied by two independent cell lawns (cell A on left, cell B on right) grown for 24 h on a 60LB agar plate. The round rim distance (*d*) of two cell lawns from each group was set at 0.5 (**Figure** **2**a), 1, and 1.5 cm (Figure S9, Supporting Information) for comparative analysis. The resultant formation of interactive patterns was captured by microscopy under bright‐ and dark fields, respectively, exhibiting similar fluorescent gradients determined by AHL molecules diffusion from collaborative cell lawn (Figure 2a; Figure S9, Supporting Information). Expectedly, enhanced expression of QS‐based control panels enabled stronger cell‐cell communication capability of two interactive cell lawns with FI level increased by up to 60% (Figure 2a). Moreover, the maximum interactive distance *d* enabling detected fluorescence can reach up to 1.5 cm when QS‐based control panels are driven by P_porin183_ and P_porin226_ promoters (Figure S9, Supporting Information). Therefore, the optimized QS‐based collaborative systems showed improved signaling and sensing performance, enabling sufficient dynamic control uses in scalable mixed cultural conditions.

For many metabolic engineering cases of *Halomonas* TD, controlling the recombinant cells from the “cell growing” to the “cell working” stage dynamically is a long‐term challenge due to the lack of sufficient genetic tools. In previous studies, the overexpression of septum site‐determining proteins (MinCD) and *Vitreoscilla* hemoglobin (VHb) allowing stronger oxygen uptake capability can achieve larger cell space for PHA granules accumulation and improved growth under limited oxygen supply conditions,^[^ ^49^, ^50^ ^]^ respectively. However, these attempts affected the growth in lag‐ and log‐phase with reduced cell population while constitutively expressing target genes. Herein, constructs harboring MinCD and VHb encoded genes driven by a QS‐based collaborative system based on P_porin183_ group from Figure 2a were designed for prototyping application tests in shake flask cultivation (Figure 2b). As shown in Figure 2c, the mixed cultures of cells A and B containing MinCD expression control panels showed obvious fiber‐shape cells at different mixing time points (0, 8, and 12 h, right row). In contrast, the cells from independent cultures of start host TD1.0, and cells A and B maintained rod‐shape (left row). Besides, controlling VHb expression precisely by mixing cells A and B (upper panel, mixed at A4:B1 and A1:B4; bottom panel, mixed at A1:B1) at 8 and 12 h, respectively, resulting in 75% improvement of cell dry weight (CDW) with enhanced PHB accumulation compared to the control group carrying plasmids with constitutive VHb expression^[^ ^51^ ^]^ (Figure 2d). In conclusion, the prototyping applications of the QS‐based collaborative dynamic system exemplified successful cases in gene expression control in non‐model *Halomonas* chassis.

### 2.3 Chromosomal Integration of QS‐Based Control Panels For Improved Scale‐Up Robustness

**Figure 3:**
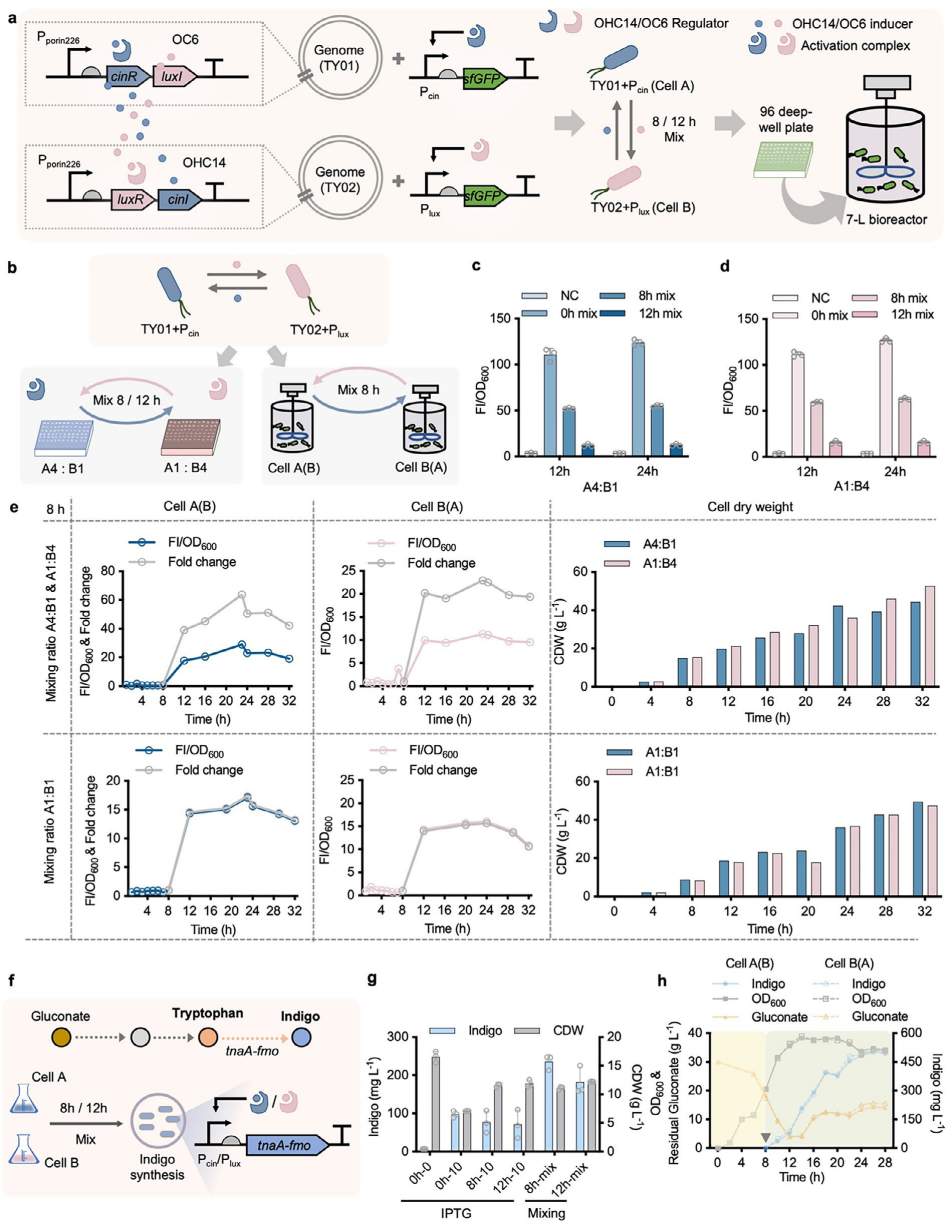
Chromosomal integration of QS‐based control panels for improved scale‐up robustness. a) Schematic design for chromosomal integration of QS‐based control panels. Two gene clusters including *cinR‐luxI* and *luxR‐cinI* under the control of P_porin226_ were independently integrated on locus G53 of *Halomonas* TD1.0, forming two recombinant strains, TY01 (cell A) and TY02 (cell B), respectively. Constructs containing sfGFP expression modules driven by P_cin_ and P_lux_ were conjugated into TY01 (TY01+P_cin_) and TY02 (TY02+P_lux_), respectively, for dynamic control assessment. b) Schema for assessing QS‐based collaborative dynamic control system in different cultural scales, including 96‐deep well plate and 7‐L bioreactor. c. d) Dynamic control assessment of mixed culturing cells A and B at different time points (0, 8, and 12 h after inoculation). Two mixed cultural groups of cells A and B, namely A4:B1 in blue and A1:B4 in pink, were grown in a 96‐deep well plate and sampled twice for FI measurement after 12 h (1st sampling) and 24 h (2nd sampling) mixing growth, respectively. e) Scale‐up assessment of QS‐based collaborative dynamic control system. Pre‐cultures of cells A and B were mixed at 4:1 (upper panel) and 1:1 (bottom panel) ratios, respectively, after 8 h independent growth in two 7‐L bioreactors. Data points plotted in the left and middle rows, as well as histograms in the right row, show sfGFP expression levels and CDW accumulation from two independent mixed cultural groups of each mixed ratio. f) Schema for enhanced indigo synthesis from gluconate and tryptophan by dynamically regulating the expression of *tnaA*‐*fmo* cluster in recombinant TY01 and TY02. g) Shake flask studies of indigo production by overexpressing *tnaA*‐*fmo* cluster controlled by QS‐based collaborative control system at different growth time points against IPTG‐induced system (10 mg L^−1^). h) Fed‐batch study of indigo production from gluconate and tryptophan in 7‐L bioreactors. OD_600_, residual gluconate, and indigo titer from each bioreactor were recorded every 4 h. The Inverted triangle in grey indicates the cell mixing time point (8 h) with an aim to activate the expression of *tnaA*‐*fmo* cluster for initiating indigo synthesis. FI values in c to e were measured by microplate reader after cell cultures diluted to 0.2–0.8 of OD_600_ and normalized by dividing OD_600_ value (FI/OD_600_). Data points in c, d, and g are shown as mean ± SD of three replicates. Fed‐batch fermentations in e and h were respectively conducted in two parallel 7‐L bioreactors for the mixed culture of cells A and B after 8 h fermentation.

To achieve robust dynamic control performance in scale‐up fermentation test, the QS‐based control panels, *cinR‐luxI* and *luxR‐cinI*, were independently integrated on the chromosome of TD1.0 (locus G53), forming TY01 (cell A) and TY02 (cell B) strains, respectively (**Figure** **3**a). The chromosomal expression of QS‐based control panels was controlled by P_porin226_, the strongest promoter tested in Figure 2a, due to the expression level reduction from the high‐copy plasmid‐carried system to single‐copy chromosome‐based system reported in the previous study.^[^ ^52^ ^]^ Dose‐response characterization of recombinant cells A and B harboring sfGFP expression vessel driven by P_cin_ (TY01+P_cin_) and P_lux_ (TY02+P_lux_) promoters, respectively, revealed high usability for further dynamic control uses mediated by mixed culturing (Figure S10, Supporting Information). Mixed culturing of these two types of cells at 4:1 (A4:B1 and A1:B4) at different time points (0, 8 and 12 h) in 96 deep‐well plate showed indeed obvious dynamic expression foldchange in contrast to the NC group (Figure 3b–d). The dynamic induction performance of sfGFP at single cell level was assessed by flow cytometer, showing over 84% cell event of positive fluorescence with Gaussian‐like distribution pattern in all mixed cultures (1:1 and 1:4) (Figure S11, Supporting Information). However, the slight variance of expression level between two cell populations was still observed (Figure S11e, Supporting Information), which could be further relieved by engineering the expression of QS regulators, signal molecule synthesis and promoter core region of P_cin_ and P_lux_ according to the previous reports.^[^ ^3^, ^34^, ^53^ ^]^ Besides, the population proportions of cells A and B in different mixed cultures (1:1 and 1:4) were studied by introducing double fluorescent reporters, sfGFP and CyOFP1 controlled by P_lux_ and P_cin_ (Figures S12a and S13a, Supporting Information), respectively. The cell population proportion of cells A and B could be stably maintained around at the mixing ratios after 12 and 24 h growth with fluorescent positive events reaching over 90% (Figures S12b and S13b).

For the scale‐up test in 7‐L bioreactors, recombinant cells were mixed at the ratios of 4:1 (upper panel) and 1:1 (bottom panel), respectively, after 8 h independent pre‐culturing (Figure 3e). Time‐course FI measurement throughout the fermentation process indicated effective sfGFP expression after mixing growth at 8 h. Interestingly, the mixed cultural group of A4:B1 showed higher dynamic foldchange compared to the A1:B4 one (≈64‐fold versus ≈23‐fold) because of the stronger ON‐stage expression and lower OFF‐stage leakiness of P_cin_ promoter, which was a dominant control system in A4:B1 group. In contrast, the FI of mixed groups at 1:1 ratio showed similar expression levels and dynamic foldchange, however, lower than that of A4:B1 group. Moreover, a comparative analysis of sfGFP expression measured by flow‐cytometer at single cell level against microplate reader during fed‐batch study was implemented, manifesting robust expression pattern in both level and fluorescent positive event (78%) throughout 32 h fermentation (Figure S14, Supporting Information).

For the case‐study test, the key synthesis pathway of indigo,^[^ ^54^ ^]^ which is a value‐added dye affecting cell growth once intracellular synthesis starts, encoded by *tnaA‐fmo* cluster was constructed in TY01 and TY02 strains (Figure 3f) compared to IPTG‐induced system in TD1.0 used as a control (Table S3, Supporting Information). In a shake flask study, over 235 mg L^−1^ indigo could be obtained from tryptophan by controlling the expression of *tnaA‐fmo* after 8 h growth (Figure 3g), which was 1.5‐fold higher than that induced by 10 mg L^−1^ IPTG with similar induction output (FI) characterized by sfGFP (Figure S15, Supporting Information). The indigo titer was further increased up to 500 mg L^−1^ by mixing recombinant cells at 8 h (inverted triangle in grey) after 28 h fed‐batch fermentation conducted in 7‐L bioreactors (Figure 3h). In summary, the chromosomally integrated QS‐based control panels empowered sufficient and robust dynamic gene expression control with foldchange greater than 15‐fold by recombinant *Halomonas* TD under high cell density, which were induced with cell mass reaching over 15 g L^−1^ (CDW) in lab‐scale fed‐batch culturing condition. Besides, a slight decrease of FI/OD_600_ value of all mixed cultural groups was observed in the latent growth phase after 24 h growth probably because of the PHB accumulation with the dramatic increase of OD_600_ under limited nitrogen source feeding (Figures S14 and S16, Supporting Information). Moreover, enhanced production of indigo can be achieved by a QS‐based collaborative dynamic control system whether conducted in batch or fed‐batch conditions, which demonstrates promising potential use in microbial cell factory engineering. However, optimizing dynamic foldchange and designing dynamic inhibitory control are still required for diverse gene expression control demands of different levels and functions.

### 2.4 Engineering Dynamic Inhibitory Control by Cascading CRISPRi Repression System

In addition to the dynamic activation control demonstrated in previous figures, dynamic inhibitory control was further developed incorporation of the CRISPRi repression system with an aim to expand the control functions. First, CRISPRi system consisting of dCas9 and gRNA expression modules were constructed on single‐ (pSEVA321, Figure S17a, Supporting Information) and double‐plasmid (pSEVA321/341, Figure S17b, Supporting Information) systems, respectively, for assessing gene inhibition performance in TD‐GFP strain, which contains *sf*GFP expression unit controlled by P_porin226_ on locus G7 derived from TD1.0. Interestingly, the double‐plasmid system showed effective inhibition on sfGFP with over 74% decrease of FI level under different transcribed levels of gRNA induced by 2 to 100 mg L^−1^ IPTG. In contrast, only 53% of sfGFP repression was observed in the presence of 2 mg L^−1^ IPTG by the single‐plasmid group. Nevertheless, varied expression levels of *d*Cas9 driven by P_porin221_, P_porin287,_ and P_porin194_ promoters displayed negligible effects on gene inhibition efficiency (Figure S18, Supporting Information) compared to the design driven by P_J23117_ (Figure S17b, Supporting Information). The prototyping CRISPRi repression system was thus established in TD‐GFP based on the double‐plasmid system.

**Figure 4:**
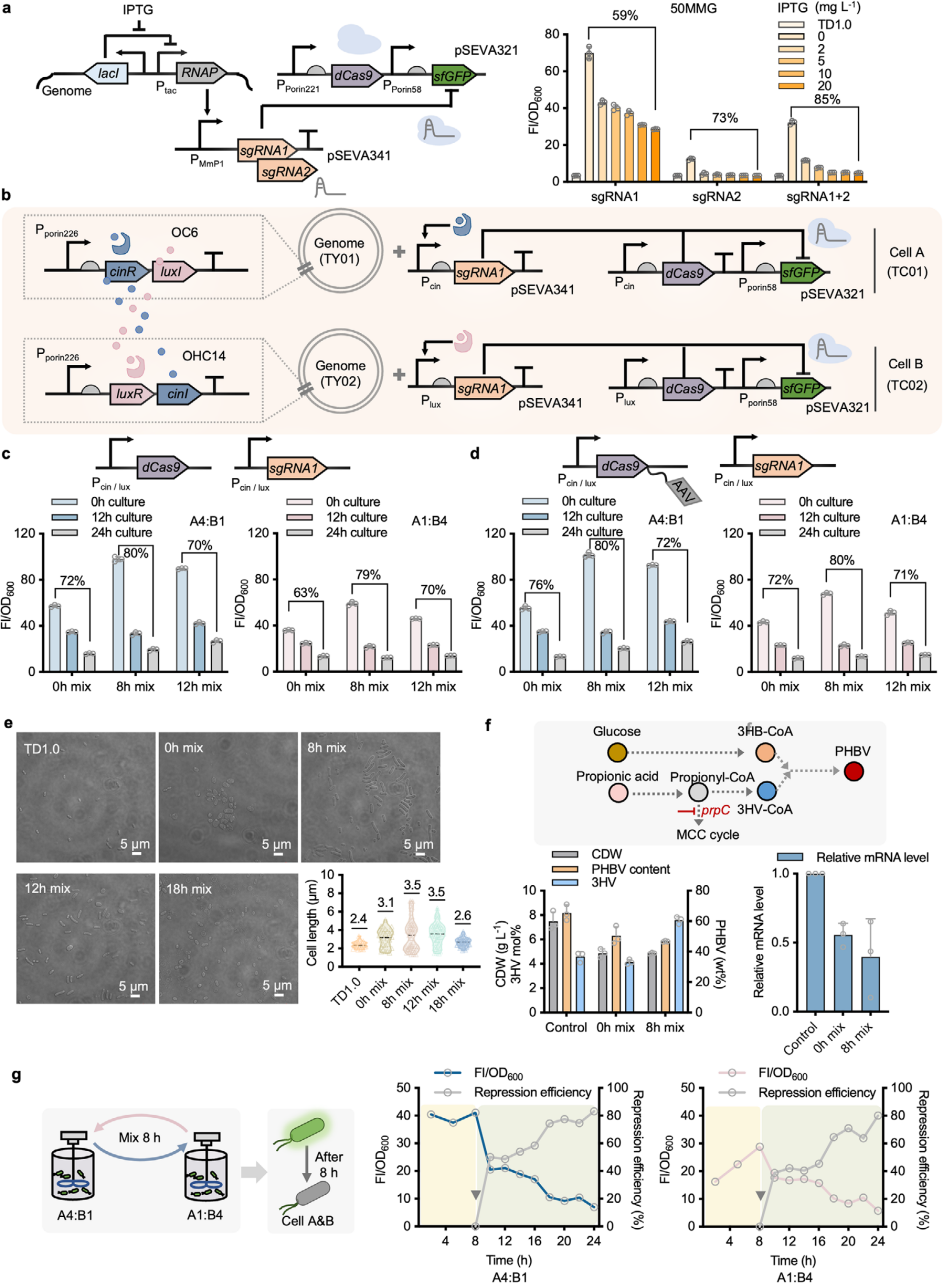
Engineering dynamic inhibitory control by cascading CRISPRi repression system. a) Construction and verification of CRISPRi repression system in *Halomonas* TD. The repression performance of three sgRNA combinations controlled by P_MmP1_ was characterized in 50MMG medium after 24 h cell growth in the presence of 0, 2, 5, 10, and 20 mM IPTG, respectively. b) Schematic circuit design of prototyping CRISPRi inhibitory system activated by mixed culturing cells A and B dynamically, namely QS‐based collaborative dynamic inhibitory control. Specifically, sgRNA1 and *d*Cas9 controlled by P_cin_ and P_lux_ promoters instead of P_MmP1_, from part a were constructed in TY01 and TY02 strains, respectively, forming cells A (TC01) and B (TC02). c) Assessment of repression efficiency by mixing cells A and B after 0 h (0 h mix), 8 h (8 h mix), and 12 h (12h mix) pre‐culture independently. The *sf*GFP expression levels of pre‐cultured cells A and B right before mixing were used as the control group (0h culture) against the repressed groups after 12 h (12 h culture) and 24 h (24 h culture) mixing growth. d) A degradation tag AAV was added to the C‐terminal of dCas9 to study the effect on repression activity. e) Morphological changes of recombinant cells were studied by dynamically inhibiting cell morphology‐related gene, *mreB*, using a QS‐based CRISPRi repression system from part d. Cell length of captured cells A and B mixed (1:1) at 0, 8, 12, and 18 h was measured by ImageJ for quantitative comparison in contrast to the start host, TD1.0. f) Prototype test of QS‐based CRISPRi repression system for PHBV production from glucose and propionate by dynamically inhibiting *prpC* encoding 2‐methylcitrate synthase. The relative mRNA level of the repressed *prpC* gene was measured compared to the wide‐type TD1.0 strain used as a negative control. g) Fed‐batch study of repression efficiency was conducted in two 7‐L bioreactors by recombinant *Halomonas* cells harboring a QS‐based CRISPRi system using sfGFP as a reporter from part d. The time‐course normalized FI was recorded every 4 h. The repression efficiency (lines in gray) was thus calculated based on the FI/OD_600_ value measured at 8 h, the mixing time point of cells A and B (inverted triangle). FI values were measured by a microplate reader after cell cultures were diluted to 0.2‐0.8 of OD_600_ and normalized by dividing OD_600_ (FI/OD_600)_. Data points in parts a, and c to f are shown as mean ± SD of three replicates. The regression foldchange of each QS‐based CRISPRi system in e to f was shown at the top of the histograms. Fed‐batch fermentations in g were conducted in two parallel 7‐L bioreactors for independent pre‐culture of cells A and B, and mixed culture after 8 h.

Next, two guide RNAs, sgRNA1 and sgRNA2, and their combination (sgRNA1+2) driven by P_MmP1_ were designed to test the repression efficiency of *sf*GFP incorporation of constitutively expressed *d*Cas9 driven by P_porin221_ based on the double‐plasmid system. The repression performances of three sgRNA combinations were characterized in 50MMG medium after 12 and 24 h growth, respectively, in the presence of 0, 2, 5, 10, and 20 mg L^−1^ IPTG (**Figure** **4**a; Figure S19 and Table S1, Supporting Information). By contrast, sgRNA1 exhibited the best performance with a lower leakage‐repression effect and significant repression activity. Subsequently, to construct a QS‐based inhibitory control system with a further reduction of leakage‐repression, the expression control of both sgRNA1 and dCas9 was replaced by P_cin_ and P_lux_ promoters and constructed in TY01 and TY02 strains, namely TC01 (cell A) and TC02 (cell B), respectively (Figure 4b). Therefore, the mixed culture of cells A and B can dynamically inhibit the expression of sfGFP with reduced FI levels. Expectedly, obvious gene repression can be achieved by mixing cells A and B at 0, 8, and 12 h, respectively, with over 63% decrease in FI/OD_600_ values. Mixed cultures at 8 h displayed the highest repression level, reaching up to 80%. However, the initial mixed culturing group (0 h mix) showed lower basal FI level (histograms of 0 h culture) but poorer repression performance due to a lack of sfGFP accumulation phase compared to another two groups mixing at 8 and 12 h (Figure 4c). Moreover, a degradation tag AAV was added to the C‐terminal of dCas9 aiming to reduce its leakage expression (Figure 4d). However, negligible improvement can be obtained, even incorporated with promoter replacement by P_luxCATG_, a P_lux_ mutant of lower leakiness (Figure S20a, Supporting Information). Notably, the addition of an AAV tag to sfGFP could significantly improve the repression efficiency, which reached up to 97% and 91% in A4:B1 and A1:B4 mixed culturing groups, respectively (Figure S20b, Supporting Information). Meanwhile, the basal FI levels decreased obviously because of enhanced degradation activity of sfGFP (Figure S20b, Supporting Information). In summary, dynamic inhibitory control of effectiveness has been established by cascading CRISPRi repression system based on QS‐based collaborative dynamic control design.

For the proof‐of‐concept study, the optimized QS‐based inhibitory control circuit was employed to inhibit the expression of cell morphology‐related gene *mreB* in recombinant TC01 and TC02 strains by removing sfGFP module and replacing sgRNA1 with sgRNA*_mreB_* (Tables S1 and S2, Supporting Information). Therefore, the precultures of recombinant cells A and B were mixed at different time points, including 0, 8, 12, and 18 h, to obtain visualized morphological changes under confocal microscopy (Figure 4e). The cell length of captured cells measured by ImageJ revealed obvious cell‐shape changes compared to the control group by wild‐type TD1.0. Interestingly, the mixed growth cells varied dramatically in shape at different mixing time points, namely growth phases, showing large sphere‐shape mixed at 0 h and then turning to be longer rod‐shape when mixed at 8 and 12 h, however, smaller sphere cells were observed when mixed at 18 h. These results uncovered that dynamic repression of the *mreB* gene at different cell growth phases enabled different cell shape manipulation compared to the previous study focusing on only gene knock‐out with large sphere‐shape formation.^[^ ^55^ ^]^ Besides, as another prototype test, QS‐based CRISPRi repression was also employed to generate PHA copolymers consisting of 3‐hydroxybutyrate (3HB) and 3‐hydroxyvalerate (3HV), namely PHBV,^[^ ^56^ ^]^ from glucose and propionate by dynamically inhibiting the bypass activity encoded by *prpC* gene, which able to converts propionate into MCC cycle (Figure 4f). Shake flask results revealed that the molar ratio of 3HV was increased by nearly 1‐fold, reaching up to 8 mol%, compared to the control group by TD1.0 grown in the same condition. Similarly, RT‐qPCR profiling was simultaneously performed to verify that the transcription level of *prpC* was significantly decreased by over 60% after gene repression triggered at 8 h. However, poorer growth of the mixed cultural group (8 h mix) can be observed probably due to the overexpression of dCas9. For the fed‐batch study of dynamic inhibitory control, recombinant cells A and B from Figure 4d were grown in a 7‐L bioreactor independently for 8 h and then mixed at 1:4 (A1:B4 and A4:B1) to activate the inhibitory system using sfGFP as a reporter. Obviously, FI levels of the two mixed groups exhibited a remarkable decrease by over 80% (Figure 4g). The time‐course repression dynamics and efficiency, as well as CDW and PHB accumulation (Figure S21, Supporting Information), were recorded every 4 h throughout the fed‐batch study, showing a similar repression pattern between two mixed cultural groups. These results thus demonstrated the strong potential of QS‐based dynamic inhibitory control systems for scale‐up industrial uses in the coming future.

### 2.5 Constructing High‐Performing Amplifier System by Cascading MmP1 RNA Polymerase

**Figure 5:**
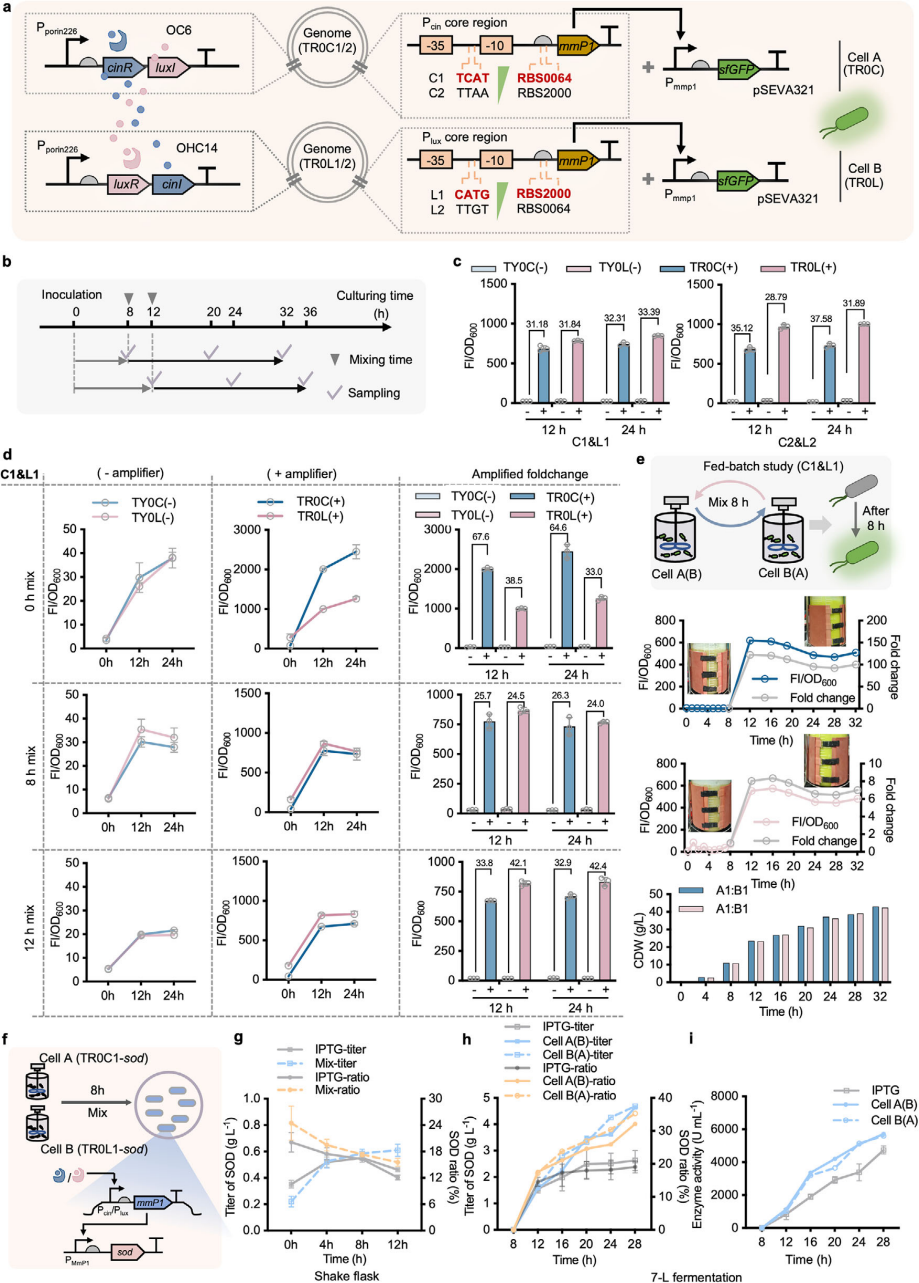
Constructing a QS‐based collaborative amplifier by dynamically controlling the expression of MmP1 RNA polymerase. a) Schematic design of QS‐based amplifier circuits. MmP1 RNA polymerase (MmP1) driven by P_cin_ (C1+RBS0064 & C2+RBS2000) and P_lux_ (L1+RBS2000 & L2+RBS0064) of different transcription and translation strengths were constructed on the genome locus GME_16862‐GME_16 867 of TY01 and TY02 strains, respectively, forming 4 recombinant strains, TR0C1/2 and TR0L1/2. Furthermore, *sf*GFP controlled by P_MmP1_, activated by MmP1, was constructed on the plasmid‐carried system for amplified expression control in recombinant in TR0C1/2 (cell A) and TR0L1/2 (cell B) strains, respectively. b) Characterization workflow for testing the dynamic control performances of QS‐based amplifier by cell‐A and ‐B mixed cultures. Inverted triangles represent the mixing time points of cells A and B, and ticks indicate the timing of cell culture sampling for FI measurement. c) Comparative analysis of two amplifier designs based on part a, TR0C1+TR0L1 (C1&L1) and TR0C2+TR0L2 (C2&L2), namely TY0C(+) and TY0L(+), against the control groups of TY0C(‐) and TY0L(‐) by mixing cells A and B after 12 h preculture in 60LB independently. d) Dynamic amplification control of the C1&L1 group was assessed by mixing cells A and B after 0 h (upper panel), 8 h (middle panel), and 12 h (bottom panel) independent growth in 96 deep‐well plates in 60LB. FI/OD_600_ of QS‐based collaborative dynamic activation circuits without (“−”) and with (“+”) amplifier (C1&L1) were shown for comparative analysis. ‘0 h’ In the *x‐axis* (left and middle rows) indicates the “OFF‐state” right before cell mixing compared to the “ON‐state” after 12  and 24 h mixed culture. Histograms with amplified foldchange were shown in the right row. e) Scale‐up test of QS‐based amplifier (C1&L1) by recombinant *Halomonas* cells was conducted in two parallel 7‐L bioreactors. FI/OD_600_ and CDW of each bioreactor were recorded every 4 h. f) Prototype test of QS‐based amplifier control for effective enzyme production, which is similar to protein‐producing chassis *E. coli* DE3, using superoxide dismutase (SOD) as demo product. g) Shake flask studies of SOD production, including titer and protein ratio (SOD/total protein), at different induction time points (0, 4, 8, and 12 h) by QS‐based amplifier compared to IPTG‐induced system in the presence of 50 mg L^−1^ IPTG. h and i) Fed‐batch study of SOD (7‐L bioreactor) by mixing cells after 8 h cultivation. The protein titer, ratio (h), and enzyme activity (i) of SOD from each bioreactor were recorded every 4 h. FI values were measured by microplate reader after cell cultures diluted to 0.2–0.8 of OD_600_ and normalized by dividing OD_600_ (FI/OD_600_). “+” and “−” In c and d indicate recombinant strains with and without amplifier designs, respectively. Data points in c, d, and g are shown as mean ± SD of three replicates, fed‐batch fermentation in e, h and i were respectively conducted in two parallel 7‐L bioreactors for independent pre‐culture of cells A and B, and mixed culture after 8 h.

Although the QS‐based collaborative dynamic control systems have been constructed and tested in lab‐scale fermenters, the dynamic range under OFF‐to‐ON control is still limited for genes requiring high‐level expression (Figure 3c–e). To address this purpose, genetic amplifiers were designed by cascading MmP1 as a signal propagator, a T7‐like RNA polymerase, controlled by different P_cin_/P_lux_ and RBS combinations, forming TR0C1/2 strains (cell A harboring C1/C2 designs) and TR0L strains (cell B harboring L1/L2 designs) derived from TY01 and TY02, respectively (**Figure** **5**a). In addition, *sf*GFP reporter under the control of P_MmP1_ triggered by MmP1 polymerase was constructed in TR0C and TR0L strains on the plasmid‐carried system for FI analysis according to the designed characterization workflow (Figure 5a,b). Two paired combinations of recombinant cells A and B, namely C1&L1 (left panel) and C2&L2 (right panel), were first tested by mixing at 1:1 ratio after 12 h independent growth in a 96 deep‐well plate. Accordingly, expression vessels containing *sf*GFP controlled by P_cin_ (C1+RBS0064 & C2+RBS2000) and P_lux_ (L1+RBS2000 & L2+RBS0064) were constructed in TY01 and TY02 strains, respectively, used as control groups, namely TY0C(‐) and TY0L(‐), for amplified foldchange calculation. Notably, almost over 30‐fold amplification foldchange with FI/OD_600_ reaching greater than 680 (a.u.) can be obtained from two mixed culturing groups of each amplifier design after 12 and 24 h mixed growth (Figure 5c). Compared to the obvious expression variance between two mixed culturing groups of C2&L2 designs, C1&L1 displayed better consistency of sfGFP expression level, which is more suitable for practical uses of scale‐up purpose.

Furthermore, the dynamic control performance of QS‐based amplifier systems, C1&L1 (Figure 5d; Figure S22, Supporting Information) and C2&L2 (Figures S23 and S24, Supporting Information), at different mixing time points, including 0 h (upper panel), 8 h (middle panel) and 12 h (bottom panel), were characterized in both 60LB and 50MMG media. All mixed growing groups of two designs maintained at least 20‐fold amplification of FI/OD_600_, compared to the control groups, TY0C(‐) and TY0L(‐). Generally, higher amplification foldchange and expression level of *sf*GFP (FI/OD_600_ > 1000) can be achieved when initially mixing cells A and B (0 h) compared to that mixed at 8 and 12 h. Longer mixed growth time in 50MMG medium (24 vs 12 h) would be more likely to result in decreased FI levels because of the turnover demand of intracellular protein resources under nitrogen‐limited conditions (Figures S22 and S24, Supporting Information). By contrast, the mixed growing cells in 60LB medium maintained similar expression output of sfGFP whatever after 12 or 24 h growth (Figure 5d; Figure S23, Supporting Information). Finally, a fed‐batch study by recombinant cells A and B harboring C1&L1 amplifier design was conducted in two parallel 7‐L bioreactors. FI levels of sfGFP, as well as CDW, were off‐line recorded every 4 h throughout the fermentation process. Similarly, fast‐response of amplified sfGFP expression in a saturated level can be achieved in 4 h, from 8 to 12 h, right after cells mix at 1:1 ratio (Figure 5e). In contrast to the original design (Figure 3e), the QS‐based amplifier exhibited better maintenance of sfGFP abundance during the fermentation process (Figure 5e). Therefore, in contrast to the well‐studied protein‐producing chassis like *E. coli* BL21 DE3 using IPTG as an inducer, the QS‐based collaborative amplifier could be used to achieve high‐level enzyme production by recombinant *Halomonas* TD allowing significant cost reduction under inducer‐free and unsterile condition. For the proof‐of‐concept study, the SOD of strong antioxidative activity encoded by *sod* ^[^ ^57^ ^]^ was constructed on a QS‐based amplifier system in TR0C1 and TR0L1 strains (Figure 5f), as well as IPTG‐induced system in TD1.0 used as a control, which was used for protein synthesis in previous studies.^[^ ^58^, ^59^ ^]^ The titer and protein ratio of SOD induced by cell mixing and 50 mg L^−1^ IPTG, respectively, at 0, 4, 8, and 12 h after inoculation was first assessed in shake flask studies. Expectedly, the cell mixing groups at 8 and 12 h showed better performance than the ones induced by IPTG at the same time point (Figure 5g; Figure S25, Supporting Information). More importantly, longer growth before mixed cultural induction could significantly improve the titer of SOD (Figure 5g; Figure S25, Supporting Information). Finally, a fed‐batch study for SOD production was performed by recombinants harboring a QS‐based amplifier system against an IPTG‐induced system conducted in 7‐L bioreactors. Both the SOD titer and enzyme activity from the two mixed culturing groups were dramatically higher than the IPTG‐induced group after 28 h cultivation with *sod* induced at 8 h (Figure 5h,i; Figure S26, Supporting Information). Interestingly, the total protein amount of all trials was maintained at a similar level throughout the fermentation process (Figure S26b, Supporting Information), indicating more sufficient expression of SOD under high cell density conditions triggered by QS‐based amplifier rather than IPTG induction.

Therefore, the construction of a QS‐based amplifier enables high‐level gene expression control of robustness at a desired time point (or cell density) under fed‐batch cultivation, providing an ingenious and effective tool for dynamic gene expression control over time and level in recombinant *Halomonas* with promising application value in biomanufacturing based on Next Generation Industrial Biotechnology (NGIB).

## 3 Discussion

QS‐based dynamic control in response to accumulated cell‐cell communication signals (AHLs) has been intensively studied aiming to achieve customized gene expression control of different functions over time, level, and space.^[^ ^20^ ^]^ It thus provides a promising strategy for up‐ and/or down‐regulation of genes of interest triggered by cell density only, which is easy to implement during the fermentation process conducted in varied scales.^[^ ^60^ ^]^ However, the cell‐density threshold able to activate dynamic control functions is generally not controllable. In most cases, the threshold of OD_600_ value is pretty low, which could result in premature functioning during fed‐batch fermentation and, even worse, during the seed preparation process in industrial fermentation conducted in a ton‐scale bioreactor that requires precultured cells grown in a large‐size bioreactor, generally one‐tenth of final fermentation volume, to obtain higher seed cell density (Figure S27, Supporting Information). Therefore, in this study, a series of QS‐based collaborative dynamic control systems enabling up‐ (Figures 3 and 5) or down‐ (Figure 4) regulation of target genes under high cell density (CDW > 15 g L^−1^) have been successfully constructed in NGIB chassis, *Halomonas* TD, by harnessing the advantage of contamination‐resistance fermentation process under high‐pH and high‐salt conditions, which allows easy and flexible operations for mixing cell cultures at any desired time point.^[^ ^44^, ^45^, ^61^ ^]^ First, QS‐based collaborative dynamic control circuits were constructed and optimized by tuning the expression of QS‐based control panels from plasmid‐ to chromosome‐based systems. Accordingly, the expression foldchange of *sf*GFP between ON and OFF stages reached over 15‐fold by mixing recombinant cells A and B grown in 7‐L bioreactors (Figures 1 and 3). Besides, over 70% improvement of CDW was obtained by dynamically manipulating the expression of VHb associated with oxygen assimilation driven by a QS‐based control system compared to the constitutive expression group (Figure 2d). Second, a dynamic inhibitory control system was developed by cascading CRISPRi/dCas9 module to achieve effective repression of target genes with up to 90% inhibitory efficiency conducted in 7‐L bioreactors (Figure 4). Notably, the morphological changing dynamic of recombinant *Halomonas* TD from large‐sphere, long‐rod to smaller‐sphere cells was first uncovered by inhibiting the expression of MreB associated with morphology formation in different cell growth phases (Figure 4e). These results indicated that the functions of genes involved in cell‐morphology and cell‐division control may vary a lot according to the differences in cooperative protein types and abundance under different growth phases. Thirdly, a high‐performing QS‐based amplifier was designed to generate ultrahigh‐level gene expression control dynamically by cascading MmP1 polymerase as a signal amplification processing device, which resulted in over 30‐time of amplification foldchange with FI/OD_600_ value approximately reaching greater than 700 from mixed cultures of recombinant cells A and B whatever grown in 96 deep‐well plate or 7‐L bioreactor (Figure 5). All efforts in this study thus demonstrated outstanding scale‐up robustness and broad application potential of QS‐based collaborative dynamic control systems for various microbial engineering purposes. Nevertheless, the expression level variance of target genes from two mixed culturing groups is still difficult to eliminate completely, which needs more attempts to work it out in future studies, but currently 1:1 (vol) mixing can greatly minimize this effect (Figure 3e).

To our knowledge, the open and non‐sterilized fermentation by recombinant *Halomonas* confers a simple and convenient cell mixing operation process when implementing QS‐based collaborative dynamic control functions.^[^ ^44^, ^62^ ^]^ Similarly, for traditional fermentation required restricted sterilization,^[^ ^63^ ^]^ large‐volume cell mixing could be also easily executed by transferring cell cultures of A and B at a given ratio into each seed culture fermenter or storage container, then mixed with each other by gassing pressure only based on common industrial fermentation system. This means that the QS‐based collaborative dynamic control systems have strong usability and versatility across different microbial chassis. In addition, developing bi‐function^[^ ^36^ ^]^ and multi‐function^[^ ^64^ ^]^ QS‐based control circuits by introducing different orthogonal QS‐based input modules,^[^ ^2^, ^30^, ^41^ ^]^ signal processing devices like sigma factor and RNA polymerase,^[^ ^64^, ^65^ ^]^ could further expand the dynamic control patterns involving complex genes and pathways manipulation in different time points and levels within cell consortia consisted of diverse cell types. More interestingly, this study also provides an effective dynamic control tool to explore the underlying mechanism of gene functions and cell activities over the growth phases, such as cell morphology formation affected by defective MreB (Figure 4e).

In conclusion, the QS‐based collaborative dynamic control systems developed in this study exemplify a standardized, streamlined, and ingenious method for dynamic metabolic engineering with promising control performance and robustness in scale‐up fermentation contexts.

## 4 Experimental Section

### Strains, Media, and Chemicals

All strains used in the study are listed in Table S1 (Supporting Information). *E. coli* S17‐1 was used to construct expression plasmids and promoter mutant library and used as conjugation donor.^[^ ^66^ ^]^ *Halomonas* TD1.0^[^ ^67^ ^]^ was derived from *Halomonas* TD01 isolated from Aydingol Lake (Xinjiang province, China)^[^ ^62^ ^]^ by chromosomally integrating MmP1 RNAP expression module on genomic locus GME_16862‐GME_16 867. *Halomonas* TY01 and TY02 derived from TD1.0 were constructed by integrating *cinR‐luxI* and *luxR‐cinI* clusters under the control of P_porin226_ on locus G53, respectively. Four strains including *Halomonas* TR0C1/2 and TR0L1/2 were constructed by replacing the promoter and RBS sequences of MmP1 on the genome of TD1.0 strain using P_cin_ (TR0C1, C1+RBS0064; TR0C2, C2+RBS2000) and P_lux_ (TR0L1, L1+RBS2000; TR0L2, L2+RBS0064) designs, respectively. In this study, *E. coli* S17‐1 and its derivates containing plasmids were grown in an LB medium composed of 10 g L^−1^ tryptone, 5 g L^−1^ yeast extract and 10 g L^−1^ NaCl. *Halomonas* TD01 and its derivatives used and constructed in this study were cultivated in a 60 LB medium composed of 10 g L^−1^ tryptone, 5 g L^−1^ yeast extract and 60 g L^−1^ NaCl, or mineral medium (50MMG) containing 30 g L^−1^ glucose, 50 g L^−1^ NaCl, 1 g L^−1^ yeast extract, 0.5 g L^−1^ urea, 0.2 g L^−1^ MgSO_4_, 9.65 g L^−1^ Na_2_HPO_4_·12H_2_O, 1.5 g L^−1^ KH_2_PO_4_, 10 ml L^−1^ trace element solution‐III and 1 ml L^−1^ trace element solution‐IV.^[^ ^62^, ^68^ ^]^ Antibiotics including 25 mg L^−1^ chloramphenicol and/or 100 mg L^−1^ spectinomycin were added to the media whenever necessary. The pH of cultural media was initially adjusted to 9.0–10.0 before inoculation using 5 M NaOH for a 96‐deep‐well plate and shake flask study by *Halomonas* TD01 and its derivatives. For fed‐batch study, the basic cultural medium (4 L in total) is composed of (g L^−1^): 50 NaCl, 20 glucose, 12 yeast extract, 2 urea, 0.2 MgSO_4_, 3.5 KH_2_PO_4_, 40 mL trace element III (1 vol%) and 4 mL trace element IV (1 vol‰). In addition, feeding solution I (Feed‐I) consists of (g) 267 glucose and 13 urea, while feeding solutions II and III (Feed‐II/III) contain (g) 333 glucose, 1.3 urea, 2.4 (NH_4_)_2_SO_4_, and 400 glucose only, respectively. Feed‐I/II/III were subsequently fed into a 7‐L bioreactor to maintain the residual glucose (g L^−1^) at 5 to 10 measured by medical glucometer (SANNUO, China) throughout the fed‐batch fermentation process of each trial. Relevant antibiotics were initially added in the basic medium right before inoculation whenever necessary for the growth of recombinant cells carrying plasmids. All chemicals were purchased from Sinopharm Chemical Reagent (China) and Sigma‐Aldrich (USA). All DNA sequences of encoding genes were synthesized by Beijing Genomics Institute (BGI, China), and PCR primers were synthesized by Sangon Biotech. (China).

### Conjugation

First, *E. coli* S17‐1 harboring target plasmids were used as donor cells after 12–14 h growth in LB medium. Recombinant strains of *Halomonas* grown in 60LB medium for 12 h were used as recipient cells. Both donor and recipient cells were centrifuged and harvested under 4 °C at 1500×g for 2 min (Cence, TGL‐16, China), washed once with fresh 20LB medium composed of 10 g L^−1^ tryptone, 5 g L^−1^ yeast extract and 20 g L^−1^ NaCl, and recovering to the same volume that sampled. Subsequently, cells were mixed (1:1 volume) and dropped on (60–80 µL) an antibiotic‐free agar plate (20LB) for 8–12 h incubation at 37 °C. Finally, the conjugated cell lawn was suspended using fresh 60 LB medium and spread on (100–200 µL) a 60 LB agar plate supplemented with relevant antibiotics for 36–48 h incubation at 37 °C to acquire positive colonies for further PCR verification.

### Plasmid Construction

The plasmids used in this study were constructed via Golden Gate^[^ ^69^ ^]^ or Gibson assembly.^[^ ^70^ ^]^ Sequences of genes used in this study were listed in Table S2 (Supporting Information). To construct QS‐based collaborative dynamic control circuits, genes encoding core QS‐control panels including *cinR*, *cinI*, *luxR*, *luxI*, and corresponding induced promoters (P_cin_ and P_lux_ containing operator sequences) were synthesized by Beijing Genomics Institute (BGI, China). The *cinR‐luxI* and *luxR‐cinI* modules driven by P_lacI,_ and P_porin_ mutants, including P_porin68_, P_porin42_, P_porin183,_ and P_porin226,_ as well as *sf*GFP controlled by P_cin_ and P_lux_ (and/or their mutants) were constructed on pSEVA321 vector to generate constructs of pP_lacI_‐*cinR‐luxI*‐P_cin_‐*sf*GFP, pP_porin68_‐*cinR‐luxI*‐P_cin_‐*sf*GFP, pP_porin42_‐*cinR‐luxI*‐P_cin_‐*sf*GFP, pP_porin183_‐*cinR‐luxI*‐P_cin_‐*sf*GFP, pP_porin226_‐*cinR‐luxI*‐P_cin_‐*sf*GFP, pP_lacI_‐*luxR‐cinI*‐P_lux_‐*sf*GFP, pP_porin68_‐*luxR‐cinI*‐P_lux_‐*sf*GFP, pP_porin42_‐*luxR‐cinI*‐P_lux_‐*sf*GFP, pP_porin183_‐*luxR‐cinI*‐P_lux_‐*sf*GFP and pP_porin226_‐*luxR‐cinI*‐P_lux_‐*sf*GFP for characterization. Plasmids pP_porin183_‐*cinR‐luxI*‐P_cin_‐*minCD*, pP_porin183_‐*cinR‐luxI*‐P_cin_‐*vgb*, pP_porin183_‐*luxR*‐*cinI*‐P_lux_‐*minCD*, pP_porin183_‐*luxR*‐*cinI*‐P_lux_‐*vgb*, pP_cin_‐*tnaA‐fmo* and pP_lux_‐*tnaA‐fmo* were constructed for prototyping applications of QS‐based collaborative dynamic control system, in recombinant *Halomonas*. To construct QS‐based collaborative CRISPRi system, sgRNAs and dCas9 driven by P_J23117_ and P_MmP1_, respectively, were first constructed on single‐ (pSEVA321) and double‐ (pSEVA321 and pSEVA341 containing P_J23117_‐dCas9 and P_MmP1_‐sgRNA modules, respectively) plasmid systems for CRISPRi‐based gene repression test based on the design in previous study.^[^ ^71^, ^72^ ^]^ In particular, dCas9 controlled by different P_porin_ mutants^[^ ^48^ ^]^ was constructed based on a double‐plasmid system to study the effect on gene repression activity. Besides, two sgRNA sequences of 20‐nt length (sgRNA1 and sgRNA2), located in upstream of the PAM sequence (NGG, “N” represents any deoxynucleotide of A, T, C or G) of sfGFP encoded gene, were selected for CRISPRi characterization in this study. Second, expression vessels harboring dCas9 and sgRNAs modules controlled by P_cin_ and P_lux_, independently and respectively, were constructed based on the double‐plasmid system for the prototyping test of QS‐based collaborative CRISPRi system. Besides, plasmids including dCas9 controlled by P_lux‐CATG_ mutant of lower leakiness^[^ ^3^ ^]^ (pP_lux‐CATG_‐*d*Cas9) and degradation tag AAV added to the C‐terminal of dCas9 (pP_cin/lux‐CATG_‐*d*Cas9*‐AAV*) and sfGFP (pP_porin58_‐*sf*GFP*‐AAV*) were also constructed to study the repression effects. Finally, the QS‐based collaborative CRISPRi designs, including pP_cin/lux_‐*d*Cas9*‐AAV*+pP_cin/lux_‐sgRNA*_mreB_*, pP_cin/lux_‐*d*Cas9‐AAV+pP_cin/lux_‐sgRNA_prpC_ and pP_cin/lux_‐*d*Cas9*‐AAV*‐P_porin58_‐*sf*GFP+pP_cin/lux_‐sgRNA1 were constructed for dynamic manipulation of cell morphology, PHBV synthesis, and scale‐up test using sfGFP as reporter conducted in a 7‐L bioreactor, respectively. For QS‐based collaborative amplifier construction, three major control panels, including *cinR*‐*luxI* and *luxR‐cinI* clusters controlled by P_porin_ mutant (sensing panel), MmP1 controlled by P_lux/cin_ and their derivates (amplified panel), as well as P_MmP1_‐*sf*GFP (reporter panel on plasmid‐carried system only), were designed and constructed on plasmid‐ and/or chromosome‐carried systems (see Table S3, Supporting Information), respectively, for amplification characterization. Then the reporter *sf*GFP controlled by P_MmP1_ involved in the QS‐based amplifier was replaced by *sod* gene for sufficient enzyme synthesis. Additionally, genome editing of *Halomonas* TD and its derivates was performed by using CRISPR/Cas9‐^[^ ^56^ ^]^ and homologous recombination‐^[^ ^59^ ^]^ based methods, which were developed in previous studies.

### Mixed Culture of Recombinant Cells A and B of Different QS‐based Dynamic Control Systems

Single colonies of recombinant cells A and B of different QS‐based dynamic control systems were first grown in 60LB containing relevant antibiotics for 12 h. Subsequently, pre‐cultures were 1 vol% inoculated into fresh 60LB medium for 12 h growth to obtain seed cultures for further experiments. Finally, the obtained seed cultures of different recombinant cells were inoculated into 60LB and/or 50MMG media for independent pre‐culturing in 96‐deep‐well plate (1 vol% inoculation, 1 mL medium in total), shake flask (5 vol% inoculation, 20/50 mL medium in total) and 7‐L bioreactor (10 vol% inoculation, 4 L medium in total), respectively. The independent pre‐cultures of cells A and B of varied cell density, depending on the preculturing time including 0, 8, 12, and 18 h, were next mixed at designed ratios for further assessment of QS‐based collaborative dynamic control performance. All mixed culture processes were carried out under open conditions. For the fed‐batch study, dissolved oxygen (DO%) was maintained over 30% via increasing the agitation rate up to 800 rpm and air injection with a maximum flow rate of 1 VVM (air volume per culture volume per minute). Meanwhile, the pH was maintained at 8.5 adjusted by a 5 m NaOH solution. All batches of 7 L fermentation were conducted at 37 °C. And 2 mL cell cultures were harvested every hour for fluorescence (microplate reader, 488/520 nm) and OD_600_ measurement, and 35 mL cell cultures were harvested every 4 h for CDW and PHB content assay.

### Fluorescent Intensity Characterization

Generally, single colonies of recombinant *Halomonas* cells were first picked as an inoculum for 12 h incubation in 60LB and/or 50MMG media containing relevant antibiotics whenever necessary. Subsequently, the first seed cultures were 1 vol% inoculated into fresh 60LB/50MMG media supplementary with relevant antibiotics for 12 h growth in a 96‐deep‐well plate and shake flask for fluorescence assessment. Different concentrations of inducers including IPTG, OHC14, and OC6 were initially supplemented into the medium for characterizing the dose‐response curves of MmP1‐(P_MmP1_) and QS‐ (P_cin_ and P_lux_) based induced systems, respectively. For *sf*GFP characterization of varied QS‐based collaborative dynamic control systems, independent and/or mixed cultural cell A and B grown in 60LB and 50MMG media in 96‐deep‐well plate, shake flask and 7‐L bioreactor (T&J Bio‐engineering, T&J‐Intelli‐Ferm B 7L, China), respectively, were harvested at different points as needed (see the workflow in Figure 1a) for FI measurement by flow‐cytometer (100‐fold dilution) and a microplate reader (diluted to 0.2–0.8 of OD_600_), respectively. For off‐line measurement of *sf*GFP throughout the fed‐batch study of QS‐based collaborative dynamic control systems, 35 mL cell cultures from each parallel bioreactor were sampled every 4 h for FI (microplate reader), OD_600,_ and CDW assessment. PHB content was also analyzed whenever necessary. Specially, harvested cells were first centrifuged to collect cell pellets, followed by resuspension using PBS buffer solution by recovering the same sampled volume before measuring fluorescence intensity and OD_600_, aiming to remove the unknown interferences from the growth medium. Besides, the resuspended cell cultures were diluted 100‐fold using PBS buffer solution for flow‐cytometer analysis with HTS attachment (CytoFELX, Beckman Coulter, USA). Fluorescence‐positive cells were captured by the channels of forward scatter (FSC, 40 gain value) and side scatter (SSC, 384 gain value), with the excitation spectrum of 488 nm (B525 channel with 21 gain value for *sf*GFP and B585 channel with 18 gain value for *Cy*OFP1). Cytometry data were analyzed by FlowJo software (v10.7) for quantitative determination of FI (mean value of captured cell counts in arbitrary units). For microplate reader analysis, the resuspended cell cultures were diluted to 0.2‐0.8 of OD_600_ with PBS buffer for FI (488/520 nm, Varioskan Flash, Thermo Scientific) and OD_600_ measurement. Normalized FI (FI/OD_600_) was obtained by dividing OD_600_ for further data analysis.

### Confocal Microscope Analysis

Cells of *Halomonas* TD1.0 and its derivates harboring morphology‐control circuits (cell A and cell B, and their mixed cultures (1:1, vol) mixing at different time points) grown in a 150 mL shake flask containing 20 mL 60LB medium supplemented with 30 g L^−1^ glucose were sampled after 24 h independent and/or mixed growth at 37 °C. Subsequently, 10 µL of cell culture was sampled for morphology observation by confocal microscope (LSM 880, Zeiss, Germany) under a light field with randomly shot photos.

### Imaging Analysis of Interacting Patterns

Recombinant cell cultures of cells A and B harboring different QS‐based collaborative dynamic control circuits were dropped on a 60LB agar plate (with antibiotics) to obtain paired circular cell lawns with given distances (0.5, 1, and 1.5 cm) after 24 h incubation. The visualized interacting pattern of each paired group was generated by an Olympus SZX16 camera under Olympus U‐RFL‐T mercury lamp light coupled with different filters (GFP: 436/20 EX filter and 480/40 EM filter). Photos were further processed and adjusted by ImageJ (NIH, USA) to determine the relative fluorescent intensity. The obtained images and data are shown in Figure 2a and Figure S9 (Supporting Information).

### PHAs Production Analysis

The assays of PHB and PHBV content were performed based on the protocol developed in the previous study.^[^ ^59^ ^]^ 30 mL of cell cultures were sampled for 10 min centrifugation at 10000 ×g (Cence, H1750R, China), followed by resuspension using water and washed twice to harvest the cell pellet for overnight lyophilization. CDW of freeze‐dried cells was measured, and PHA content assays were carried out by GC (gas chromatography, GC‐2014, SHIMADZU, Japan). PHB of analytical purity purchased from Sigma‐Aldrich (St. Louis, MO, USA) and 3‐Hydroxyvaleric acid of 98% purity purchased from Macklin Biochemical Technology (Shanghai, China) were used as standards. Shake flask studies for PHB production were conducted by recombinant *Halomonas* TD strains harboring different *vgb* expression modules grown in 500 mL shake flasks at 37 °C. Specifically, recombinant cells A and B, which harbored Tat‐*vgb* expression module driven by P_cin_ (cell A) and P_lux_ (cell B) promoters, respectively, were grown in 50MMG medium independently and mixed at 4:1 and 1:1 ratio, respectively, at 8 and 12 h after inoculation (5 vol%), respectively, to induce the expression of *vgb* gene. In contrast, the start host, TD1.0, harboring Tat‐*vgb* expression module driven by constitutive promoter P_porin_ grown in the same condition was used as a control group. Shake flask studies for PHBV production were conducted by recombinant *Halomonas* TD strains harboring pP_cin/lux_‐*d*Cas9‐AAV+pP_cin/lux_‐sgRNA_prpC_ *prpC* repression CRISPRi modules, grown in a 20 mL shake flask at 37 °C. Recombinant cells A and B, which harbored the *prpC* repression module driven by P_cin_ (cell A) and P_lux_ (cell B) promoters, respectively, were grown in 50MMG medium independently and mixed at 1:1 ratio (0 and 8 h after inoculation) to induce the expression of *d*Cas9 and sgRNA*_prpC_*. Besides, 0.5 g L^−1^ propionic acid used as the precursor of 3HV was added at 12 h for PHBV synthesis. The start host, TD1.0, grown in the same condition was used as the control group. All recombinant cells were cultured for 48 h in total after inoculation. For the fed‐batch study, CDW and PHB content were measured every 4 h throughout each batch of fermentation conducted in a 7‐L bioreactor.

### Indigo Synthesis and Analysis

Single colonies of recombinant cell A (*tnaA‐fmo* cluster controlled by P_cin_), cell B (*tnaA‐fmo* cluster controlled by P_lux_), and recombinant TD1.0 (*tnaA‐fmo* cluster controlled by P_MmP1_) were first grown in 60LB containing relevant antibiotics for 12 h. Subsequently, pre‐cultures were 1 vol% inoculated into fresh 60LB medium for 12 h growth to obtain seed cultures for further experiments. Finally, the obtained seed cultures were 5 vol% inoculated into a modified 50 MM medium supplemented with 36 g L^−1^ gluconate and 3 g L^−1^ urea. The independent pre‐cultures of cells A and B were mixed at 1:1 ratio (8 and 12 h after inoculation) to initiate the expression of *tnaA‐fmo*. In contrast, recombinant TD1.0 harboring *tnaA‐fmo* cluster controlled by P_MmP1_ induced by 10 mg L^−1^ IPTG at 0, 8, and 12 h, respectively, were used as control groups. Particularly, 1.5 g L^−1^ tryptophan was added into the cell cultures for indigo synthesis together with cell mixing or IPTG addition. For fed‐batch study, dissolved oxygen (DO%) was maintained over 30% via adjusting agitation (≤800 rpm) and air injection (≤1 VVM, air volume per culture volume per minute). The basic cultural medium (4 L in total) is composed of (g L^−1^): 50 NaCl, 30 gluconate, 10 yeast extract, 3 urea, 0.2 MgSO_4_, 3.5 KH_2_PO_4_, 40 mL trace element III (1 vol%) and 4 mL trace element IV (0.1 vol%). In addition, the feeding solution consists of (g) 300 gluconate, 43 urea, and 11 yeast extract. The feed solution was fed into a 7‐L bioreactor to maintain the residual gluconate at 15 g L^−1^ measured by high‐performance liquid chromatography (HPLC, LC‐16, Shimadzu, Japan) throughout the fed‐batch fermentation process. Meanwhile, the pH was maintained at 8.5 adjusted by 5 M NaOH and 20% (v/v) phosphoric solutions. And 35 mL cell cultures were sampled and harvested in every 4 h for residual gluconate, indigo titer, OD_600,_ and CDW measurement. For the indigo assay, 100 µL cell cultures were sampled and harvested via centrifugation at 13 000×g for 5 min. The indigo was then extracted from sediment by adding dimethyl sulfoxide (DMSO). Followed by 5 min centrifugation at 13 000×g, indigo in supernatants was measured by a microplate reader (Varioskan LUX, Thermo Scientific, USA) based on the absorbance at 610 nm (OD_610_) to determine the titer of indigo.^[^ ^73^ ^]^

### SOD Synthesis and Analysis

Single colonies of recombinant cell A (TR0C1 harboring p321‐P_MmP1(without LacO)_‐*sod*), cell B (TR0L1 harboring p321‐P_MmP1(without LacO)_‐*sod*), and recombinant TD1.0 harboring p321‐P_MmP1_‐*sod* were first grown in 60LB containing relevant antibiotics for 12 h. Subsequently, pre‐cultures were 1 vol% inoculated into fresh 60LB medium for 12 h growth to obtain seed cultures for further experiments. Finally, the obtained seed cultures of different recombinant cells were 5 vol% inoculated into a modified 50MMG medium supplemented with 5 g L^−1^ urea in a 20 mL shake flask. The independent pre‐cultures of cells A and B were 1:1 (vol%) mixed at 0, 4, 8, and 12 h after inoculation, respectively, for dynamically regulating the expression of *sod*. All mixed culture processes were carried out under open conditions. Recombinant TD1.0 harboring *p321‐P_MmP1_‐sod* grown in the same condition was induced by 50 mg L^−1^ IPTG at 0, 4, 8, and 12 h after inoculation, respectively, as control groups. For the fed‐batch study of sod, dissolved oxygen (DO%) was maintained over 30% via adjusting the agitation (≤800 rpm) and air injection with a maximum flow rate of 1 VVM (air volume per culture volume per minute). The basic cultural medium (4 L in total) is composed of (g L^−1^): 50 NaCl, 20 glucose, 12 yeast extract, 2 urea, 0.2 MgSO_4_, 3.5 KH_2_PO_4_, 40 mL trace element III (1 vol%) and 4 mL trace element IV (0.1 vol%). In addition, feeding solution I (Feed‐I) consists of (g) 267 glucose and 13 urea. Feed‐I was fed into a 7‐L bioreactor to maintain the residual glucose (g L^−1^) at 5 to 10 measured by a medical glucometer (SANNUO, China) throughout the fed‐batch fermentation process. Meanwhile, the pH was maintained at 8.5 adjusted by a 5 M NaOH solution. All batches of 7 L fermentation were conducted at 37 °C. And 35 mL cell cultures were sampled and harvested every 4 h to measure the residual glucose, OD_600_, CDW, PHB content and titer and activity of SOD. For SOD analysis, 1 mL of cell cultures were sampled and harvested by 5 min centrifugation (13 000 × g) at 4 °C. The resulting sediment was then resuspended using a PBS buffer solution for cell disruption by ultrasonic homogenizer (JY92‐II N, SCIENTZ, China). Followed by 5 min centrifugation (13 000×g) at 4 °C to obtain supernatant with soluble SOD for SDS‐PAGE analysis using YoungPAGE 4%–20% Bis‐Tris gels (GenScript Biotech, M00928, USA). The amount of total soluble proteins was determined by the BCA protein assay kit (SmartBuffers, DFR‐0500, China). Gel lanes were scanned by the flatbed scanner (EPSON, Perfection V39II A4, Japan). Protein ratio of SOD in total soluble proteins was calculated based on the shade intensity of gel bands determined by Quantity One (version 4.6.2, Bio‐Rad, USA). The SOD activity was determined using SOD Activity Assay Kit, Micromethod (Sangon Biotech, D799594, China).

### RT‐qPCR Profiling

To quantify the repression level of *prpC*, total RNA was isolated from TD1.0 and the mixed culture of TY01 and TY02 harboring *prpC* repression module (24 h growth) by RNAprep Pure Cell/Bacteria Kit (TIANGEN Biotech, DP430, China). Total RNA concentration was measured by microspectrophotometer (ALLSHENG, Nano‐400A, China). Then 1 µg of target RNA was used as a template for inverse transcription using FastKing gDNA Dispelling RT Kit (TIANGEN, KR118, China). The concentration of cDNA was diluted to 10 ng µL^−1^ as a template for mRNA quantification of *prpC*, and to 0.1 ng µL^−1^ as a template for 16S rRNA quantification to normalize the mRNA reads of *prpC*. All qPCR assays were implemented using 2×RealStar Fast SYBR qPCR Mix (GenStar, A301, China) on a fluorescent quantitative PCR system (CFX Connect, Bio‐Rad USA). Relative mRNA levels of *prpC* under different conditions were calculated using the 2^−ΔΔCt^ method.

## Conflict of Interest

The authors declare no conflict of interest.

## Author Contributions

Y.N.L. and Y.X.L. contributed equally to this work. J.W.Y. and Y.N.L. proposed the idea and designed the experiments. Y.N.L. and Y.X.L. constructed and optimized a series of QS‐based collocative dynamic control systems. Y.N.L. and Y.X.L. performed the shake flasks. Y.N.L., Y.X.L., Y.Z, and Y.H.D performed the fed‐batch fermentation experiments. Y.N.L., Y.X.L, K.X.L, Y.G., H. L., J.W., J.W.P., and R.Z.D. performed other experiments. Y.N.L. and Y.X.L. analyzed the data and wrote the manuscript. J.W.Y., Y.N.L., H.W., and H.M.W. revised the manuscript. All authors have read and approved the final manuscript.

## Supporting information



Supporting Information

## Data Availability

The data that support the findings of this study are available from the corresponding author upon reasonable request.
