# Supplementary material for: Developing Quorum Sensing‐Based Collaborative Dynamic Control System in Halomonas TD01
Source: Adv Sci (Weinh). 2025 Mar 16;12(18):2408083. doi: 10.1002/advs.202408083 (PMC12079531; doi:10.1002/advs.202408083)
Supplement: Supplementary file 1 — Supporting Information [file ADVS-12-2408083-s001.docx]

Supporting Information

**Developing Quorum Sensing-based Collaborative Dynamic Control System in *Halomonas* TD01**

**Authors:**

Yi-Na Lin^1,5^, Yu-Xi Li^1,5^, Ye Zheng^2,3,4^, Yi-Hao Deng^1^, Kai-Xuan Liu^1^, Yue Gan^1^, Hao Li^1^, Jun Wang^1^, Jia-Wen Peng^1^, Rui-Zhe Deng^2,3,4^, Huai-Ming Wang^2,3,4^, Hui Wang^2,3,4^, Jian-Wen Ye^1*^

**Affiliations:**

^1^ School of Biology and Biological Engineering, South China University of Technology, Guangzhou 510006, China.

^2^ Department of General Surgery (Colorectal Surgery), The Sixth Affiliated Hospital, Sun Yat-sen University, Guangzhou 510655, China

^3^ Guangdong Provincial Key Laboratory of Colorectal and Pelvic Floor Diseases, The Sixth Affiliated Hospital, Sun Yat-sen University, Guangzhou 510655, China

^4^ Biomedical Innovation Center, The Sixth Affiliated Hospital, Sun Yat-sen University, Guangzhou 510655, China

^5^ Co-first authors contribute equally in this study: Yi-Na Lin, Yu-Xi Li.

***Corresponding author:**

Jian-Wen Ye: yejianwen@scut.edu.cn (Ye JW);

School of Biology and Biological Engineering, South China University of Technology, Guangzhou 510006, China

Supplementary Tables

**Table S1 Strains and genes used in this study.**

| **Strains/ Genes** | **Descriptions** | **References** |
| --- | --- | --- |
| *E. coli* S17-1 | A vector donor used for conjugation, harbors the *tra* genes from plasmid RP4 in the chromosome | Simon et al., 1983^[1]^ |
| *Halomonas.* TD01 | Wild-type *Halomonas* TD01 strain isolated from Aydingkol Lake in Xinjiang Province, China | Tan et al., 2011^[2]^ |
| *Halomonas* TD1.0 | *Halomonas* TD01 integrated with MmP1 RNA polymerase expression module | Zhao et al., 2017^[3]^ |
| TD-GFP | Genomic integration of P_porin226_-*sf*GFP in TD1.0 strain | This study |
| TY01 | Genomic integration of P_porin226_-*cinR-luxI* expression unit in G53 loci of TD1.0 strain | This study |
| TY02 | Genomic integration of _Pporin226_-*luxR-cinI* expression unit in G53 loci of TD1.0 strain | This study |
| TR01C1 | Genomic replacement of P_tac_ promoter by P_cin-TACT_ together with RBS_0064 to control the expression of Mmp1 RNA Polymerase encoded gene in TY01 | This study |
| TR01C2 | Genomic replacement of P_tac_ promoter by P_cin-TTAA_ together with RBS2000 to control the expression of Mmp1 RNA Polymerase encoded gene in TY01 | This study |
| TR02L1 | Genomic replacement of P_tac_ promoter by P_lux-CATG_ together with RBS2000 to control the expression of Mmp1 RNA Polymerase encoded gene in TY02 | This study |
| TR02L2 | Genomic replacement of P_tac_ promoter by P_lux-TTGT_ together with RBS0064 to control the expression of Mmp1 RNA Polymerase encoded gene in TY02 | This study |
| **Genes** |  |  |
| *cinR* | Encoding QS-regulators CinR with strong OHC14-binding affinity, forming OHC14-CinR complex to activate the transcription activity of P_cin_ promoters | This study |
| *cinI* | Encoding QS-signal molecule (OHC14) synthesis protein, CinI | This study |
| *luxR* | Encoding QS-regulators LuxR with strong OC6-binding affinity, forming OC6-LuxR complex to activate the transcription activity of P_lux_ promoters | This study |
| *luxI* | Encoding QS-signal molecule (OC6) synthesis protein, LuxI | This study |
| *minCD* | Cell division inhibitor associated with cell elongation | Tan et al., 2014^[4]^ |
| *vgb* | Encoding VHb protein allowing enhanced oxygen availability of recombinant cell | Ouyang et al., 2018^[5]^ |
| *tnaA* | Encoding tryptophanase from *Escherichia coli* W3110 | Du et al., 2018^[6]^ |
| *fmo* | Encoding monooxygenase from *Methylophaga aminisulfidivorans* | Du et al., 2018^[6]^ |
| *dCas9* | Encoding deactivated SpCas9 (dCas9) derived from *Streptococcus pyogenes* | Tao et al., 2017^[7]^ |
| *MmP1* | Encoding T7-like RNA polymerase, MmP1 | Zhao et al., 2017^[3]^ |
| *mreB* | Encoding actin-like protein, MreB, associated with cell shape formation | Jiang et al., 2017^[8]^ |
| *prpC* | Encoding 2-methylcitrate synthase converting propionyl-CoA towards methyl citric acid cycle | Fu et al., 2014^[9]^ |
| AAV | Degradation tag, listed in Supplementary Table 2 | Wang et al., 2021^[10]^ |
| sgRNA1 | Single stranded RNA targeting to genomic integrated *sf*GFP (site 1), listed in Supplementary Table 2 | This study |
| sgRNA2 | Single stranded RNA targeting to genomic integrated *sf*GFP (site 2), listed in Supplementary Table 2 | This study |
| sgRNA*_mreB_* | Single stranded RNA targeting to *mreB*, listed in Supplementary Table 2 | This study |
| sgRNA*_prpC_* | Single stranded RNA targeting to *prpC*, listed in Supplementary Table 2 | This study |
| *sod* | Encoding superoxide dismutase (SOD) from *Escherichia coli* DE3 | Zhou et al., 2022^[11]^ |

**Table S2 Sequences of genes used in this study.**

| **Gene & degradation tag & sgRNAs** | **Nucleotide Sequence** |
| --- | --- |
| *cinR* | ATGATTGAGAATACCTATAGCGAAAAGTTCGAGTCCGCGTTCGAACAGATCAAAGCGGCGGCCAACGTGGATGCCGCCATCCGTATTCTCCAGGCGGAATATAACCTCGATTTCGTCACCTACCATCTCGCCCAGACAATCGCGAGCAAGATCGATTCGCCCTTCGTGCGCACCACCTATCCGGATGCCTGGGTTTCCCGTTACCTCCTCAACTGCTATGTGAAGGTCGATCCGATCATCAAGCAGGGCTTCGAACGCCAGCTGCCCTTCGACTGGAGCGAGGTCGAACCGACGCCGGAGGCCTATGCCATGCTGGTCGACGCCCAGAAACACGGCATCGATGACAATGGCTACTCCATCCCCGTCGCCGACAAGGCGCAGCGCCGCGCCCTGCTGTCGCTGAATGCCCATATACCGGCCGACGAATGGACCGAGCTCGTGCGCCGCTGCCGCAATGAGTGGATCGAGATCGCCCATCTGATCCACCGCAAGGCCGTATATGAGCTGCATGGCGAAAACGATCCGGTGCCGGCATTGTCGCCGCGCGAGATCGAGTGTCTGCACTGGACCGCCCTCGGCAAGGATTACAAGGATATTTCGGTCATCCTGGGCATATCAGAGCATACCACACGCGATTACCTGAAAACCGCCCGCTTCAGGCTCGGCTGCACCACGATCTCGGCCGCCGCGTCGCGGGCTGTTCAATTGCGCATCATCAATCCCTATAGGATCCGCATGACGCGACGTAATTGGTAA |
| *cinI* | ATGTTCGTTATCATTCAGGCACATGAGTATCAGAAATACGCTGCCGTACTCGACCAGATGTTTCGTCTGCGCAAGAAGGTCTTCGCCGATACGCTCTGCTGGGACGTTCCTGTCATCGGCCCTTACGAACGTGACAGCTACGATTCGCTTGCTCCCGCCTATCTCGTCTGGTGCAACGACAGCCGCACCCGTCTTTATGGCGGCATGCGCCTGATGCCGACGACCGGCCCGACCCTTCTCTACGACGTCTTCCGCGAGACGTTCCCTGATGCCGCCGATCTTATCGCCCCCGGCATCTGGGAAGGCACGCGCATGTGCATCGACGAGGAGGCGATCGCCAAGGATTTCCCCGAGATCGACGCCGGCCGCGCCTTCTCCATGATGCTGCTCGCGCTTTGCGAATGCGCGCTCGATCACGGCATCCACACGATGATCTCCAACTACGAGCCCTACCTCAAGCGCGTCTACAAGCGCGCCGGCGCCGAGGTGGAAGAACTCGGCCGCGCAGACGGCTACGGCAAATATCCCGTCTGCTGCGGCGCCTTCGAAGTCTCGGACCGCGTGCTGCGCAAGATGCGCGCCGCCCTCGGCCTCACCCTACCCCTTTATGTCAGGCACGTGCCGGCCCGCTCGGTCGTGACCCAATTCCTGGAGATGGCAGCATGA |
| *luxR* | ATGAAAAACATAAATGCCGACGACACATACAGAATAATTAATAAAATTAAAGCTTGTAGAAGCAATAATGATATTAATCAATGCTTATCTGATATGACTAAAATGGTACATTGTGAATATTATTTACTCGCGATCATTTATCCTCATTCTATGGTTAAATCTGATATTTCAATCCTAGATAATTACCCTAAAAAATGGAGGCAATATTATGATGACGCTAATTTAATAAAATATGATCCTATAGTAGATTATTCTAACTCCAATCATTCACCAATTAATTGGAATATATTTGAAAACAATGCTGTAAATAAAAAATCTCCAAATGTAATTAAAGAAGCGAAAACATCAGGTCTTATCACTGGGTTTAGTTTCCCTATTCATACGGCTAACAATGGCTTCGGAATGCTTAGTTTTGCACATTCAGAAAAAGACAACTATATAGATAGTTTATTTTTACATGCGTGTATGAACATACCATTAATTGTTCCTTCTCTAGTTGATAATTATCGAAAAATAAATATAGCAAATAATAAATCAAACAACGATTTAACCAAAAGAGAAAAAGAATGTTTAGCGTGGGCATGCGAAGGAAAAAGCTCTTGGGATATTTCAAAAATATTAGGTTGCAGTGAGCGTACTGTCACTTTCCATTTAACCAATGCGCAAATGAAACTCAATACAACAAACCGCTGCCAAAGTATTTCTAAAGCAATTTTAACAGGAGCAATTGATTGCCCATACTTTAAAAATTGA |
| *luxI* | ATGACTATAATGATAAAAAAATCGGATTTTTTGGCAATTCCATCGGAGGAGTATAAAGGTATTCTAAGTCTTCGTTATCAAGTGTTTAAGCAAAGACTTGAGTGGGACTTAGTTGTAGAAAATAACCTTGAATCAGATGAGTATGATAACTCAAATGCAGAATATATTTATGCTTGTGATGATACTGAAAATGTAAGTGGATGCTGGCGTTTATTACCTACAACAGGTGATTATATGCTGAAAAGTGTTTTTCCTGAATTGCTTGGTCAACAGAGTGCTCCCAAAGATCCTAATATAGTCGAATTAAGTCGTTTTGCTGTAGGTAAAAATAGCTCAAAGATAAATAACTCTGCTAGTGAAATTACAATGAAACTATTTGAAGCTATATATAAACACGCTGTTAGTCAAGGTATTACAGAATATGTAACAGTAACATCAACAGCAATAGAGCGATTTTTAAAGCGTATTAAAGTTCCTTGTCATCGTATTGGAGACAAAGAAATTCATGTATTAGGTGATACTAAATCGGTTGTATTGTCTATGCCTATTAATGAACAGTTTAAAAAAGCAGTCTTAAAT |
| *sfgfp* | ATGCGTAAAGGCGAAGAGCTGTTCACTGGTGTCGTCCCTATTCTGGTGGAACTGGATGGTGATGTCAACGGTCATAAGTTTTCCGTGCGTGGCGAGGGTGAAGGTGACGCAACTAATGGTAAACTGACGCTGAAGTTCATCTGTACTACTGGTAAACTGCCGGTACCTTGGCCGACTCTGGTAACGACGCTGACTTATGGTGTTCAGTGCTTTGCTCGTTATCCGGACCATATGAAGCAGCATGACTTCTTCAAGTCCGCCATGCCGGAAGGCTATGTGCAGGAACGCACGATTTCCTTTAAGGATGACGGCACGTACAAAACGCGTGCGGAAGTGAAATTTGAAGGCGATACCCTGGTAAACCGCATTGAGCTGAAAGGCATTGACTTTAAAGAAGACGGCAATATCCTGGGCCATAAGCTGGAATACAATTTTAACAGCCACAATGTTTACATCACCGCCGATAAACAAAAAAATGGCATTAAAGCGAATTTTAAAATTCGCCACAACGTGGAGGATGGCAGCGTGCAGCTGGCTGATCACTACCAGCAAAACACTCCAATCGGTGATGGTCCTGTTCTGCTGCCAGACAATCACTATCTGAGCACGCAAAGCGTTCTGTCTAAAGATCCGAACGAGAAACGCGATCATATGGTTCTGCTGGAGTTCGTAACCGCAGCGGGCATCACGCATGGTATGGATGAACTGTACAAATGA |
| *CyOFP1* | ATGGTGAGCAAAGGCGAAGAACTGATCAAAGAAAACATGCGCAGCAAACTGTACCTGGAAGGCAGCGTGAACGGCCACCAATTCAAATGCACCCACGAAGGCGAAGGCAAACCGTACGAAGGCAAACAGACCAACCGCATCAAAGTGGTGGAAGGCGGCCCGCTGCCTTTCGCCTTCGATATTCTGGCCACTCATTTCATGTACGGCAGCAAAGTGTTCATCAAATACCCGGCCGACCTGCCGGACTACTTCAAACAGAGCTTCCCGGAAGGCTTCACCTGGGAACGCGTTATGGTTTTCGAAGACGGCGGCGTTCTGACCGCCACTCAAGATACTAGCCTGCAAGATGGCGAACTGATCTATAACGTGAAAGTGCGCGGCGTGAACTTCCCGGCCAATGGCCCGGTTATGCAGAAAAAAACCCTGGGCTGGGAACCGAGCACCGAAACCATGTATCCGGCCGACGGTGGCCTTGAAGGTCGTTGTGATAAAGCCCTGAAACTGGTGGGCGGCGGCCATCTGCATGTTAACTTTAAAACCACCTACAAGAGCAAGAAGCCGGTGAAAATGCCGGGCGTGCACTACGTGGATCGCCGTCTGGAACGTATTAAAGAAGCCGATAACGAAACCTACGTGGAACAGTACGAACACGCCGTGGCCCGCTATAGCAATCTGGGCGGTGGTATGGATGAACTGTACAAATAA |
| *minCD* | ATGAGCCTCAACGCCAATAGTGCCGACATTGCCTTCACCTTCAAAGGTGGCATGCTGCCAATGACCGTCATGGAATTGAGCAGCGCTGACCCGGAACATATACGAAGTCAGCTAGCTGGCAAGTTGTCGCAATCCCCCGCGTTCTTTCAGCATACACCGGTTGTGCTGAGCGTGGAAAAACTCGATGAACCTCACTTGGCGCTTGAGCGCATTTGCGCGGTCTGTCGCGATCATAAATTATTCCCGGTAGCCGTACGTGGCGGAGCTGAACCTGTACGCCAATCTGCCTGGGCATTAGGGCTAGGCTGGGTTGCGCCTGTTGAAGAAGGGCGGACTAGGCTGTTAGAGAGCGTTGGTCCTGCCGCGATCTCTGATGACGCCATAGAGGAGGTGGAACCTGCCGAGCAGGAAGTGGTGGCGGTGGCAACACGCTTATTTCGCGGTACGGTTCGCTCTGGCCAACAGGTGAGCGCATCAGAAGGCGATCTAGTGGTGATTGGGGCAGTAAATGCGGGCGCTGAAGTGTTGGCGGCCGGTAGTATCCATGTATACGGAGCACTCCGTGGACGAGCGTTAGCGGGTATTCATGGAAATACTCAGGCGGGTATTTACTGTCGGGAATTAGAAGCAGAGCTTCTCTCCGTGGCAGGGAATTACAAACGCTTAGAAGATATTGATTCTCAGTTGCTTGGTCGCGCTACAGAGGTGCATTTCGCTCAAGAGCAGCTGGAAATTAAGCCGCTGGGATAATTGGCCAAAATTATTGTAGTGACCTCCGGTAAAGGGGGGGTTGGTAAGACCACTAGCGCTGCCGCCATTTCAACAGGCCTCGCCCTGCGTGGTAAAAAAACAGTCGTCATTGATTTCGATGTTGGTCTACGTAACCTCGACTTGATCATGGGCTGTGAGCGCCGCGTTGTTTATGACTTGGTAAACGTTATCCAAGGGGAAGCAGGGCTTAATCAGGCGCTGATTCGCGATAAACGCGTTGAAACCCTATTTATTCTCCCAGCCTCTCAAACGCGTGATAAAGATGCACTAACGCAGGAAGGCGTAGAGCGAATACTCGAGCAGCTCAAACAAGATTTTGATTTTATCTTGTGTGACTCCCCCGCAGGCATTGAGCGAGGTGCCCAGCTCGCTATGTACTTCGCTGATGAGGCGATTGTTGTCACGAATCCTGAAGTTTCCTCAGTGCGTGACTCTGACCGCATTTTGGGGCTACTTGGTTCCAAGACGCGGCGCGCTGAACAAAGCCTGGATCCGGTTAAAGAGCATTTGCTGATTACGCGCTATAACCCTTCTCGCGTAACGTCTGGGGATATGCTGACCCTGGATGACATTCGTGAAATCTTGTCTATTGATCTGCTTGGCCTCATCCCTGAATCCGAAGCGGTGCTACGTGCATCTAACCAAGGCGTTCCTGTTACTCACGATGCAGCGAGCGATGCAGGTCAGGCGTATTCAGATACTGTATCGCGCCTGTTAGGTGAAGATATGCCTCTGCGCTTCCATGAAGTACAGCGTAAGGGATTGTTGAACCGTATGTTCGGGGGTGGTCGGCGATGA |
| *Tat-vgb* | ATGAACAATAACGATCTCTTTCAGGCATCACGTCGGCGTTTTCTGGCACAACTCGGCGGCTTAACCGTCGCCGGGATGCTGGGGCCGTCATTGTTAACGCCGCGACGTATGTTAGACCAGCAAACCATTAACATCATCAAAGCCACTGTTCCTGTATTGAAGGAGCATGGCGTTACCATTACCACGACTTTTTATAAAAACTTGTTTGCCAAACACCCTGAAGTACGTCCTTTGTTTGATATGGGTCGCCAAGAATCTTTGGAGCAGCCTAAGGCTTTGGCGATGACGGTATTGGCGGCAGCGCAAAACATTGAAAATTTGCCAGCTATTTTGCCTGCGGTCAAAAAAATTGCAGTCAAACATTGTCAAGCAGGCGTGGCAGCAGCGCATTATCCGATTGTCGGTCAAGAATTGTTGGGTGCGATTAAAGAAGTATTGGGCGATGCCGCAACCGATGACATTTTGGACGCGTGGGGCAAGGCTTATGGCGTGATTGCAGATGTGTTTATTCAAGTGGAAGCAGATTTGTACGCTCAAGCGGTTGAATAA |
| *tnaA* | ATGGAAAACTTTAAACATCTCCCTGAACCGTTCCGCATTCGTGTTATTGAGCCAGTAAAACGTACCACTCGCGCTTATCGTGAAGAGGCAATTATTAAATCCGGTATGAACCCGTTCCTGCTGGATAGCGAAGATGTTTTTATCGATTTACTGACCGACAGCGGCACCGGGGCGGTGACGCAGAGCATGCAGGCTGCGATGATGCGCGGCGACGAAGCCTACAGCGGCAGTCGTAGCTACTATGCGTTAGCCGAGTCAGTGAAAAATATCTTTGGTTATCAATACACCATTCCGACTCACCAGGGCCGTGGCGCAGAGCAAATCTATATTCCGGTACTGATTAAAAAACGCGAGCAGGAAAAAGGCCTGGATCGCAGCAAAATGGTGGCGTTCTCTAACTATTTCTTTGATACCACGCAGGGCCATAGCCAGATCAACGGCTGTACCGTGCGTAACGTCTATATCAAAGAAGCCTTCGATACGGGCGTGCGTTACGACTTTAAAGGCAACTTTGACCTTGAGGGATTAGAACGCGGTATTGAAGAAGTTGGTCCGAATAACGTGCCGTATATCGTTGCAACCATCACCAGTAACTCTGCAGGTGGTCAGCCGGTTTCACTGGCAAACTTAAAAGCGATGTACAGCATCGCGAAGAAATACGATATTCCGGTGGTAATGGACTCCGCGCGCTTTGCTGAAAACGCCTATTTCATCAAGCAGCGTGAAGCAGAATACAAAGACTGGACCATCGAGCAGATCACCCGCGAAACCTACAAATATGCCGATATGCTGGCGATGTCCGCCAAGAAAGATGCGATGGTGCCGATGGGCGGCCTGCTGTGCATGAAAGACGACAGCTTCTTTGATGTGTACACCGAGTGCAGAACCCTTTGCGTGGTGCAGGAAGGCTTCCCGACATATGGCGGCCTGGAAGGCGGCGCGATGGAGCGTCTGGCGGTAGGTCTGTATGACGGCATGAATCTCGACTGGCTGGCTTATCGTATCGCGCAGGTACAGTATCTGGTCGATGGTCTGGAAGAGATTGGCGTTGTCTGCCAGCAGGCGGGCGGTCACGCGGCATTCGTTGATGCCGGTAAACTGTTGCCGCATATCCCGGCAGACCAGTTCCCGGCACAGGCGCTGGCCTGCGAGCTGTATAAAGTCGCCGGTATCCGTGCGGTAGAAATTGGCTCTTTCCTGTTAGGCCGCGATCCGAAAACCGGTAAACAACTGCCATGCCCGGCTGAACTGCTGCGTTTAACCATTCCGCGCGCAACATATACTCAAACACATATGGACTTCATTATTGAAGCCTTTAAACATGTGAAAGAGAACGCGGCGAATATTAAAGGATTAACCTTTACGTACGAACCGAAAGTATTGCGTCACTTCACCGCAAAACTTAAAGAAGTTTAA |
| *fmo* | ATGGCAACTCGTATTGCGATACTTGGTGCAGGCCCAAGTGGTATGGCACAACTCAGAGCATTCCAATCCGCCCAGGAAAAAGGTGCTGAGATCCCTGAACTCGTTTGTTTTGAAAAACAAGCTGATTGGGGCGGCCAGTGGAATTACACATGGCGCACTGGTTTAGATGAAAATGGCGAACCTGTTCATAGCAGTATGTATCGCTATCTGTGGTCAAACGGCCCGAAAGAATGTCTTGAATTTGCTGATTACACGTTTGACGAACACTTTGGTAAGCCCATCGCCTCTTATCCACCCCGTGAAGTCTTATGGGACTATATTAAAGGCCGTGTTGAAAAAGCCGGCGTCAGAAAATATATCCGTTTTAATACCGCTGTTCGTCATGTTGAATTCAACGAAGACAGCCAAACTTTTACCGTTACCGTGCAGGACCATACTACTGACACAATTTACTCTGAAGAGTTTGACTATGTTGTCTGTTGTACCGGTCACTTCTCAACACCTTACGTGCCTGAATTTGAAGGCTTTGAAAAATTTGGTGGCCGCATTCTGCATGCCCATGACTTCCGTGACGCATTAGAATTTAAAGACAAAACTGTATTACTGGTCGGCAGCAGTTACTCAGCTGAAGATATCGGCTCACAATGTTATAAATACGGCGCGAAAAAACTGATCAGCTGCTACCGTACCGCACCGATGGGTTATAAATGGCCTGAAAACTGGGATGAAAGACCCAACCTGGTTCGTGTTGATACTGAAAACGCTTATTTTGCCGATGGTTCATCAGAAAAAGTCGATGCGATTATTCTGTGTACCGGTTATATCCATCACTTCCCCTTCCTCAATGACGATCTGCGTCTGGTCACCAATAACCGTTTATGGCCGCTCAACCTTTATAAAGGCGTGGTGTGGGAAGATAATCCAAAATTCTTCTACATTGGCATGCAGGATCAATGGTACAGCTTCAATATGTTTGATGCCCAAGCCTGGTATGCCCGTGATGTGATTATGGGTCGACTGCCATTGCCATCAAAAGAAGAGATGAAAGCCGACAGCATGGCCTGGCGTGAAAAAGAACTGACGCTGGTTACGGCTGAAGAAATGTACACCTACCAGGGTGACTACATTCAGAATCTGATTGATATGACTGACTATCCGTCATTTGATATTCCGGCAACCAACAAAACTTTCCTGGAATGGAAACATCACAAAAAAGAAAACATCATGACTTTCCGTGACCACTCATACCGTTCACTGATGACTGGCACGATGGCACCGAAACATCACACACCATGGATAGATGCACTGGATGATTCTCTGGAAGCCTATCTCTCTGATAAGAGCGAAATTCCTGTGGCTAAAGAAGCTTAA |
| *dCas9* | ATGGATAAGAAATACTCAATAGGCTTAGCTATCGGCACAAATAGCGTCGGATGGGCGGTGATCACTGATGAATATAAGGTTCCGTCTAAAAAGTTCAAGGTTCTGGGAAATACAGACCGCCACAGTATCAAAAAAAATCTTATAGGGGCTCTTTTATTTGACAGTGGAGAGACAGCGGAAGCGACTCGTCTCAAACGGACAGCTCGTAGAAGGTATACACGTCGGAAGAATCGTATTTGTTATCTACAGGAGATTTTTTCAAATGAGATGGCGAAAGTAGATGATAGTTTCTTTCATCGACTTGAAGAGTCTTTTTTGGTGGAAGAAGACAAGAAGCATGAACGTCATCCTATTTTTGGAAATATAGTAGATGAAGTTGCTTATCATGAGAAATATCCAACTATCTATCATCTGCGAAAAAAATTGGTAGATTCTACTGATAAAGCGGATTTGCGCTTAATCTATTTGGCCTTAGCGCATATGATTAAGTTTCGTGGTCATTTTTTGATTGAGGGAGATTTAAATCCTGATAATAGTGATGTGGACAAACTATTTATCCAGTTGGTACAAACCTACAATCAATTATTTGAAGAAAACCCTATTAACGCAAGTGGAGTAGATGCTAAAGCGATTCTTTCTGCACGATTGAGTAAATCAAGACGATTAGAAAATCTCATTGCTCAGCTCCCCGGTGAGAAGAAAAATGGCTTATTTGGGAATCTCATTGCTTTGTCATTGGGTTTGACCCCTAATTTTAAATCAAATTTTGATTTGGCAGAAGATGCTAAATTACAGCTTTCAAAAGATACTTACGATGATGATTTAGATAATTTATTGGCGCAAATTGGAGATCAATATGCTGATTTGTTTTTGGCAGCTAAGAATTTATCAGATGCTATTTTACTTTCAGATATCCTAAGAGTAAATACTGAAATAACTAAGGCTCCCCTATCAGCTTCAATGATTAAACGCTACGATGAACATCATCAAGACTTGACTCTTTTAAAAGCTTTAGTTCGACAACAACTTCCAGAAAAGTATAAAGAAATCTTTTTTGATCAATCAAAAAACGGATATGCAGGTTATATTGATGGGGGAGCTAGCCAAGAAGAATTTTATAAATTTATCAAACCAATTTTAGAAAAAATGGATGGTACTGAGGAATTATTGGTGAAACTAAATCGTGAAGATTTGCTGCGCAAGCAACGGACCTTTGACAACGGCTCTATTCCCCATCAAATTCACTTGGGTGAGCTGCATGCTATTTTGAGAAGACAAGAAGACTTTTATCCATTTTTAAAAGACAATCGTGAGAAGATTGAAAAAATCTTGACTTTTCGAATTCCTTATTATGTTGGTCCATTGGCGCGTGGCAATAGTCGTTTTGCATGGATGACTCGGAAGTCTGAAGAAACAATTACCCCATGGAATTTTGAAGAAGTTGTCGATAAAGGTGCTTCAGCTCAATCATTTATTGAACGCATGACAAACTTTGATAAAAATCTTCCAAATGAAAAAGTACTACCAAAACATAGTTTGCTTTATGAGTATTTTACGGTTTATAACGAATTGACAAAGGTCAAATATGTTACTGAAGGAATGCGAAAACCAGCATTTCTTTCAGGTGAACAGAAGAAAGCCATTGTTGATTTACTCTTCAAAACAAATCGAAAAGTAACCGTTAAGCAATTAAAAGAAGATTATTTCAAAAAAATAGAATGTTTTGATAGTGTTGAAATTTCAGGAGTTGAAGATAGATTTAATGCTTCATTAGGTACCTACCATGATTTGCTAAAAATTATTAAAGATAAAGATTTTTTGGATAATGAAGAAAATGAAGATATCTTAGAGGATATTGTTTTAACATTGACCTTATTTGAAGATAGGGAGATGATTGAGGAAAGACTTAAAACATATGCTCACCTCTTTGATGATAAGGTGATGAAACAGCTTAAACGTCGCCGTTATACTGGTTGGGGACGTTTGTCTCGAAAATTGATTAATGGTATTAGGGATAAGCAATCTGGCAAAACAATATTAGATTTTTTGAAATCAGATGGTTTTGCCAATCGCAATTTTATGCAGCTGATCCATGATGATAGTTTGACATTTAAAGAAGACATTCAAAAAGCACAAGTGTCTGGACAAGGCGATAGTTTACATGAACATATTGCAAATTTAGCTGGTAGCCCTGCTATTAAAAAAGGTATTTTACAGACTGTAAAAGTTGTTGATGAATTGGTCAAAGTAATGGGGCGGCATAAGCCAGAAAATATCGTTATTGAAATGGCACGTGAAAATCAGACAACTCAAAAGGGCCAGAAAAATTCGCGAGAGCGTATGAAACGAATCGAAGAAGGTATCAAAGAATTAGGAAGTCAGATTCTTAAAGAGCATCCTGTTGAAAATACTCAATTGCAAAATGAAAAGCTCTATCTCTATTATCTCCAAAATGGAAGAGACATGTATGTGGACCAAGAATTAGATATTAATCGTTTAAGTGATTATGATGTCGATGCCATTGTTCCACAAAGTTTCCTTAAAGACGATTCAATAGACAATAAGGTCTTAACGCGTTCTGATAAAAATCGTGGTAAATCGGATAACGTTCCAAGTGAAGAAGTAGTCAAAAAGATGAAAAACTATTGGAGACAACTTCTAAACGCCAAGTTAATCACTCAACGTAAGTTTGATAATTTAACGAAAGCTGAACGTGGAGGTTTGAGTGAACTTGATAAAGCTGGTTTTATCAAACGCCAATTGGTTGAAACTCGCCAAATCACTAAGCATGTGGCACAAATTTTGGATAGTCGCATGAATACTAAATACGATGAAAATGATAAACTTATTCGAGAGGTTAAAGTGATTACCTTAAAATCTAAATTAGTTTCTGACTTCCGAAAAGATTTCCAATTCTATAAAGTACGTGAGATTAACAATTACCATCATGCCCATGATGCGTATCTAAATGCCGTCGTTGGAACTGCTTTGATTAAGAAATATCCAAAACTTGAATCGGAGTTTGTCTATGGTGATTATAAAGTTTATGATGTTCGTAAAATGATTGCTAAGTCTGAGCAAGAAATAGGCAAAGCAACCGCAAAATATTTCTTTTACTCTAATATCATGAACTTCTTCAAAACAGAAATTACACTTGCAAATGGAGAGATTCGCAAACGCCCTCTAATCGAAACTAATGGGGAAACTGGAGAAATTGTCTGGGATAAAGGGCGAGATTTTGCCACAGTGCGCAAAGTATTGTCCATGCCCCAAGTCAATATTGTCAAGAAAACAGAAGTACAGACAGGCGGATTCTCCAAGGAGTCAATTTTACCAAAAAGAAATTCGGACAAGCTTATTGCTCGTAAAAAAGACTGGGATCCAAAAAAATATGGTGGTTTTGATAGTCCAACGGTAGCTTATTCAGTCCTAGTGGTTGCTAAGGTGGAAAAAGGGAAATCGAAGAAGTTAAAATCCGTTAAAGAGTTACTAGGGATCACAATTATGGAAAGAAGTTCCTTTGAAAAAAATCCGATTGACTTTTTAGAAGCTAAAGGATATAAGGAAGTTAAAAAAGACTTAATCATTAAACTACCTAAATATAGTCTTTTTGAGTTAGAAAACGGTCGTAAACGGATGCTGGCTAGTGCCGGAGAATTACAAAAAGGAAATGAGCTGGCTCTGCCAAGCAAATATGTGAATTTTTTATATTTAGCTAGTCATTATGAAAAGTTGAAGGGTAGTCCAGAAGATAACGAACAAAAACAATTGTTTGTGGAGCAGCATAAGCATTATTTAGATGAGATTATTGAGCAAATCAGTGAATTTTCTAAGCGTGTTATTTTAGCAGATGCCAATTTAGATAAAGTTCTTAGTGCATATAACAAACATAGAGACAAACCAATACGTGAACAAGCAGAAAATATTATTCATTTATTTACGTTGACGAATCTTGGAGCTCCCGCTGCTTTTAAATATTTTGATACAACAATTGATCGTAAACGATATACGTCTACAAAAGAAGTTTTAGATGCCACTCTTATCCATCAATCCATCACTGGTCTTTATGAAACACGCATTGATTTGAGTCAGCTAGGAGGTGACTAA |
| *MmP1* | ATGTCAATTGCGGCGGCGGTGAACAAAAATGATTTCTCGGACGTTGAACTGGCTGCGATCCCGTTTAACACCCTGGCGGACCATTACGGTGCGGATCTGGCCCGTGAACAGCTGCAACTGGAACACGAAAGCTATGTGATGGGCGAAGAACGTTTCCGCAAAATGCTGGAACGCCAGGAAAAAGCGGAAGAATTTGGTGATAGCTCTGTTGCCAAACCGCTGATTATCACGCTGCTGCCGAAAGTCACGCAGCGTATTACCGACTGGCTGAACGAATGGGCAGATCCGAATAAAAAAGGCCGCAAACCGATTGCTTATACCCATCTGAAAGATATCAAACCGGAAACGCTGGCCTTCATTACCATCAAAGTGGTTCTGAATAAACTGGCGGGTAAAGATGACGCCTTTATGCAGCCGCTGGCATACGCTATTGGTAGTTCCATCGAAGATGAAGCACGTTTCGGCCGTATCCGCGAACTGGAAATGGCACACTTTAAAAAATGCGCTGAAGAAAACCTGAATAAACGTCGCGGCACCGCGTATCGCAAAGCCTTTCTGAGTGTCGTGGAAGCGGATATGCTGGACAAAGGTCTGCTGGGCGGTGAATCATGGGGCACGTGGAACAAAACCGATGTGATGAATATTGGTATCTCGATGCTGGAAAAACTGATTGAAGCCACGGGCCTGGTTGAACTGCGTGAAAAACGCAACTTTGAAGAAATGGATCGTATTGTCATCGCAGAAGAATACGTGAAAGCGATGGCCACCCGCGCACAGTCACTGGCTGGCATCTCGCCGATGTATCAACCGTGTGTTGTCCCGCCGAAACCGTGGGTGAGCATTACGGGCGGTGGCTACTGGGCAAACGGTCGTAAACCGACCGCTCTGATCCGTACCCATACGCGCAAAGCACTGTATCGCTACGAAGATGTTTATATGCCGGAAGTCTACAAAGCGATTAATTATGCCCAGGAAACCCCGTGGCGTATCAACCGCAAAGTGCTGGCGGTGGTTAACGAACTGGTTAAATGGAAAAACAACCCGGTCAAAGACATGCCGAGCATTGATAAACTGGAACTGCCGCAGCGTCCGGATGACATCGATACCAACGAAGAAGCGCTGCGTTCTTGGAAACGCGAAGCCGCAGCTGTTTACCGCAAAGATGAACAGCGTAAAAGCCGCTATCTGAGTATGTCCTTTGCACTGGAACAAGCTAACAAATTCTCTAACAAAAAAGCAATCTACTTCCCGTACAATATGGACTGGCGTGGCCGCGTCTATGCACTGCCGATGTTCAACCCGCAGGGTAATGATATGGTTAAAGGCCTGCTGACCCTGGCCAAAGGTAAACCGATTGGTAAAGACGGCTTTTACTGGCTGAAAATCCATGGTGCAAACACGGCTGGCGTCGATAAAGTGACCTTTCCGGAACGTATTAAATTCATCGAAGATAACCACGACAATATTATGCAGTGCGCGGAAAGCCCGCTGGACAATCTGTGGTGGACGGAACAAGATTCTCCGTTTTGCTTCCTGGCGTTTTGTTTCGAATATGCCCAGGTCACCAAAAAAGGTCTGGGCTGGGTGTGCAGTCTGCCGATTGCCCTGGATGGTTCATGTTCGGGCATCCAACACTTTTCCGCAATGCTGCGTGATGACATTGGTGGCCGCGCTGTTAACCTGCTGCCGAGCGAAACCGTCCAGGACATTTATGGTATCGTGGCAGATAAAGTTAATGAAGCTCTGAAAGAACTGGTCATCAACGGCACGGATAATTACACCGACACGGTGACCGATAAATCTACCGGTGAAATTATCGAACGTTATCGCCTGGGCGAAAAAGAACTGGCGCGTCAGTGGCTGGAATTTGGCGTCACGCGTAGCGTGACCAAACGCTCTGTGATGACCCTGGCCTACGGTTCAAAAGAATATGGCTTTCGTGACCAGGTTCTGGAAGATACGATTCGCCCGGCGATCGATTCGGGTAAAGGCGCCATGTTCACCAATCCGAGTCAAGCGGCCTCCTTTATGGCGAAACGCATTTGGGAAGCCGTGAGCGTTACCGTCGTGGCAGCTGTGGGTGCGATGAAATGGCTGCAATCATCGGCCAAACTGATGGCGGCCGAAGTGAAAGACAAGAAAACCAAAGAAGTTCTGCGTAAACGCTGCGCGGTTCATTGGGTCACCCCGGATGGTTTCCCGGTGTGGCAGGAATATCGTAAACCGAAACAAAAACGCGTTCACCTGATGTTTCTGGGTAGTTATTACGATGCGCGTATGAAAGAAACGAGCTCTGACTGTTCCATTGATGCCCATAAACAGGAAAGCGGTATCTCTCCGAACTTCGTGCATAGCCAAGATGGCAATCACCTGCGTATGACCGTTGTCTACGCGCGCGAAAAATATAACGTGGAAAGTTTTGCCCTGATTCACGACTCCTTCGGCACGATCCCGGCAGATGTTCCGAACCTGTTTAAAGCTGTGCGCGAAACCATGGTTAATATGTACGAAAACAATGACGTGCTGGCAGATTTTTATGAACAGTTCGCTGACCAACTGCATGAAAGTCAGCTGGATAAAATGCCGGCGCTGCCGCCGAAAGGTAAACTGAATCTGCAAGACATTCTGAAATCCGATTTTGCATTCGCTTAA |
| *mreB* | ATGTTCAAACGTTTGAGGGGGCTGTTTTCCAGCGATCTATCGATCGACTTGGGTACGGCCAACACACTGATTTATGTACGCGGTCGTGGCATCGTTCTCGATGAACCGTCCGTAGTCGCTATCCGCCAGTCTGGCAACATGCGCAGCGTGGCGTCGGTTGGTACCGATGCCAAGCGTATGCTCGGTCGCACGCCAGGCAATATTACCGCCATCCGCCCGATGAAAGATGGTGTTATTGCCGATTTCACCGTGACTGAGCAGATGCTTCAACACTTTATTCGTAAAGTGCATCAAAGCACCTTCTTAACGCCCAGTCCTCGTGTCTTGGTGTGCGTTCCCTGCATGTCAACGCAGGTTGAACGGCGCGCGATTCGTGAGTCGGCGGAAGGTGCTGGCGCACGCGAAGTGTTTTTGATCGAAGAGCCGATGGCCGCCGCAATTGGTGCCGGGCTTCCGGTTGAAGAAGCGCAGGGCTCAATGGTGGTTGATATCGGTGGTGGTACTACCGAAATCGCCATTATTTCGCTCAACGGCGTGGTTTATTCTGAATCTATCCGTGTCGGTGGTGACCGTTTTGATGAGGCCATCACTGCGTATGTTCGTCGTCACTACGGCAGCCTGATTGGTGAAGCCACCGCTGAGCGAATCAAAGAAGAGATCGGTTGTGCTTATCCTGGGGGCGAACTGCGCGAAATTGATGTGCGTGGTCGTAACTTGGCGGAAGGCATACCGCGTAGTTTCACACTGAACTCCCACGAGATTCTTGAAGCACTTCAAGAAACATTGGGTTCCATTGTGGCAGCGGTCAAAAGTGCTCTTGAACAATCACCGCCGGAACTAGCTTCCGATATCGCTGAGCGTGGTTTGGTGCTGACAGGTGGTGGTGCACTCTTACGTGATCTCGATAAATTGATCGCTGAAGAGACAGGCCTGCCGGTGATTGTGGCTGAAGATCCGCTCACCTGTGTGGCCCGCGGTGGCGGTAAGGCGCTGGAAATGATTGATCAGCATACCTTCGAGTTGCTGTCGAGCGACTGA |
| *prpC* | ATGGCTGATAAACCGCAAAACAGCGCAGGACTCCGTGGACAAAGCGCTGGTACCACAGCGCTGTGTACGGTGGGTAAAACGGGTTCCGGATTAACCTACCGTGGTTTTGATATTAAAGAGTTGGCCGAGAAGGCAAAGTTTGAAGAAGTCGCGTATTTGTTACTGAAAGGCAAGCTGCCTAACCAAGCCGAGCTTGATGGCTACATCACCAAGCTAAAGGGGCTGCGTGGTTTGCCCGATGCCCTGAAATCCGTGCTGGAGCAAATTCCCAAAGATGCGCATCCGATGGATGTTATGCGCACCGGTACCTCCATGCTGGGTAACCTGGAAACTGAAGAGAGCTTTGACCAGCAGCAGGATGTTTCCGATCGCCTGTTAGCGGTGCTGCCTTCGATTATTTGCTACTGGTACCGTTTCAGCCACGACGGCGTTCGCATTGATACCGAAACTGACGATGCCTCTGTAGGCGGCCACTTCCTACATATGCTGCGCGGTGAGCCTGCTTCTGAGTTACATGCGCGGGTAATGAACGTATCGTTGATTCTATACGCCGAGCATGAGTTCAATGCCTCAACGTTTACGGCGCGGGTTTGCGCCTCGACGCTTTCTGACATGCACTCTTGTGTCACCGGTGCGATTGGTTCACTACGTGGCCCGCTGCACGGCGGTGCTAATGAAGCGGCCATGGCAATGATCGAGAACTGGGCATCGCCGGAAGAAGCCGAGCGCGAAATGCTCGGTATGCTTGAGCGCAAAGAGAAGATCATGGGCTTTGGCCATGCGATTTACCGCGAGTCTGACCCGCGTAATGAAATCATCAAAGAGTGGTCGCAGAAGCTTGCCGATGACGTAGGCGACAGCGTGCTTTACCCCGTTTCTGTGCGCTGTGAAGAAGTCATGTGGCGTGAGAAGAAACTATTCTGCAACGCGGACTTCTTCCATGCCAGTGCTTACCACTTCATGGATATTCCGACCAAGCTGTTTACGCCGATCTTCGTGATGTCCCGTTTGACAGGCTGGGCAGCCCACGTATTCGAGCAGCGCGCCAATAACCGCATTATTCGCCCCAGCGCCGACTACACTGGTCCTGAGAAGAGCGAGTGGGTGCCCATCGAAGCGCGTGACTAA |
| AAV | CCTGCTGCAAACGACGAAAACTACGCTGCAGCAGTT (**PAANDENYAAAV**) |
| sgRNA1 | AAAGGCGAAGAGCTGTTCACGTTTTAGAGCTAGAAATAGCAAGTTAAAATAAGGCTAGTCCGTTATCAACTTGAAAAAGTG |
| sgRNA2 | CATCCAGTTCCACCAGAATAGTTTTAGAGCTAGAAATAGCAAGTTAAAATAAGGCTAGTCCGTTATCAACTTGAAAAAGTG |
| sgRNA*_mreB_* | CAAGTCGATCGATAGATCGCGTTTTAGAGCTAGAAATAGCAAGTTAAAATAAGGCTAGTCCGTTATCAACTTGAAAAAGTG |
| sgRNA*_prpC_* | CAAAACAGCGCAGGACTCCGGTTTTAGAGCTAGAAATAGCAAGTTAAAATAAGGCTAGTCCGTTATCAACTTGAAAAAGTG |
| *sod* | ATGTCATTCGAATTACCTGCACTACCATATGCTAAAGATGCTCTGGCACCGCACATTTCTGCGGAAACCATCGAGTATCACTACGGCAAGCACCATCAGACTTATGTCACTAACCTGAACAACCTGATTAAAGGTACCGCGTTTGAAGGTAAATCACTGGAAGAGATTATTCGCAGCTCTGAAGGTGGCGTATTCAACAACGCAGCTCAGGTCTGGAACCATACTTTCTACTGGAACTGCCTGGCACCGAACGCCGGTGGCGAACCGACTGGAAAAGTCGCTGAAGCTATCGCCGCATCTTTTGGCAGCTTTGCCGATTTCAAAGCGCAGTTTACTGATGCAGCGATCAAAAACTTTGGTTCTGGCTGGACCTGGCTGGTGAAAAACAGCGATGGCAAACTGGCTATCGTTTCAACCTCTAACGCGGGTACTCCGCTGACCACCGATGCGACTCCGCTGCTGACCGTTGATGTCTGGGAACACGCTTATTACATCGACTATCGCAATGCACGTCCTGGCTATCTGGAGCACTTCTGGGCGCTGGTGAACTGGGAATTCGTAGCGAAAAATCTCGCTGCATAA |

Letters in **bold** are amino acid sequences of protein degradation tag used in this study.

**Table S3 Plasmids used in this study.**

| **Plasmid** | **Descriptions** | **References** |
| --- | --- | --- |
| pSEVA321 | Medium copy number expression vector containing *oriT* for the expression of genes of interest in *Halomonas* TD, RK2 replication origin, Cm^R^. | Silva-Rocha et al., 2013^[12]^ |
| pSEVA341 | High copy number expression vector containing *oriT* for the expression of genes of interest in *Halomonas* TD, pRO1600 replication origin, Km^R^ and Sp^R^. | Silva-Rocha et al., 2013^[12]^ |
| pSEVA321-P_lib_-*sfgfp* | *sfgfp* under the control of *porin* promoter mutant library, P_lib_, in plasmid-based expression system, pSEVA321, Cm^R^. | Shen et al., 2018^[13]^ |
| pQ08 | pSEVA321 derivative containing cas9 expression module of *S. pyogenes* controlled by its own promoter, Cm^R^_._ | Qin et al., 2018^[14]^ |
| pP_lacI_-*cinR-luxI*-P_cin_-*sf*GFP | pSEVA321 derivative, *cinR-luxI* controlled by P_lacI_, *sf*GFP controlled by P_cin_, Cm^R^_._ | This study |
| pP_porin68_-*cinR-luxI*-P_cin_-*sf*GFP | pSEVA321 derivative, *cinR-luxI* controlled by P_porin68_, *sf*GFP controlled by P_cin_, Cm^R^_._ | This study |
| pP_porin42_-*cinR-luxI*-P_cin_-*sf*GFP | pSEVA321 derivative, *cinR-luxI* controlled by P_porin42_, *sf*GFP controlled by P_cin_, Cm^R^_._ | This study |
| pP_porin183_-*cinR-luxI*-P_cin_-*sf*GFP | pSEVA321 derivative, *cinR-luxI* controlled by P_porin183_, *sf*GFP controlled by P_cin_, Cm^R^_._ | This study |
| pP_porin226_-*cinR-luxI*-P_cin_-*sf*GFP | pSEVA321 derivative, *cinR-luxI* controlled by P_porin226_, *sf*GFP controlled by P_cin_, Cm^R^_._ | This study |
| pP_lacI_-*luxR-cinI*-P_lux_-*sf*GFP | pSEVA321 derivative, *luxR-cinI* controlled by P_lacI_, *sf*GFP controlled by P_lux_, Cm^R^_._ | This study |
| pP_porin68_-*luxR-cinI*-P_lux_-*sf*GFP | pSEVA321 derivative, *luxR-cinI* controlled by P_porin68_, *sf*GFP controlled by P_lux_, Cm^R^_._ | This study |
| pP_porin42_-*luxR-cinI*-P_lux_-*sf*GFP | pSEVA321 derivative, *luxR-cinI* controlled by P_porin42_, *sf*GFP controlled by P_lux_, Cm^R^_._ | This study |
| pP_porin183_-*luxR-cinI*-P_lux_-*sf*GFP | pSEVA321 derivative, *luxR-cinI* controlled by P_porin183_, *sf*GFP controlled by P_lux_, Cm^R^_._ | This study |
| pP_porin226_-*luxR-cinI*-P_lux_-*sf*GFP | pSEVA321 derivative, *luxR-cinI* controlled by P_porin226_, *sf*GFP controlled by P_lux_, Cm^R^_._ | This study |
| pP_porin183_-*cinR-luxI*-P_cin_-*minCD* | pSEVA321 derivative, *cinR-luxI* controlled by P_porin183_, *minCD* controlled by P_cin_, Cm^R^_._ | This study |
| pP_porin183_-*cinR-luxI*-P_cin_-*vgb* | pSEVA321 derivative, *cinR-luxI* controlled by P_porin183_, *tat-vgb* controlled by P_cin_, Cm^R^_._ | This study |
| pP_porin183_-*luxR-cinI*-P_lux_-*minCD* | pSEVA321 derivative, *luxR-cinI* controlled by P_porin183_, *minCD* controlled by P_lux_, Cm^R^_._ | This study |
| pP_porin183_-*luxR-cinI*-P_lux_-*vgb* | pSEVA321 derivative, *luxR-cinI* controlled by P_porin183_, *tat-vgb* controlled by P_lux_, Cm^R^_._ | This study |
| pP_cin_-*sf*GFP | pSEVA321 derivative, *sf*GFP controlled by P_cin_, Cm^R^_._ | This study |
| pP_cin_-*Cy*OFP1 | pSEVA321 derivative, *Cy*OFP1 controlled by P_cin_, Cm^R^_._ | This study |
| pP_cin-TCAT-RBS0064_-*sf*GFP | pSEVA321 derivative, *sf*GFP controlled by P_cin-TCAT-RBS0064_, Cm^R^_._ | This study |
| pP_cin-TTAA-RBS2000_-*sf*GFP | pSEVA321 derivative, *sf*GFP controlled by P_cin-TTAA-RBS2000_, Cm^R^_._ | This study |
| pP_lux_-*sf*GFP | pSEVA321 derivative, *sf*GFP controlled by P_lux_, Cm^R^_._ | This study |
| pP_lux_-*Cy*OFP1 | pSEVA321 derivative, *Cy*OFP1 controlled by P_lux_, Cm^R^_._ | This study |
| pP_lux-CATG-RBS2000_-*sf*GFP | pSEVA321 derivative, *sf*GFP controlled by P_lux-CATG-RBS2000_, Cm^R^_._ | This study |
| pP_lux-TTGT-RBS0064_-*sf*GFP | pSEVA321 derivative, *sf*GFP controlled by P_lux-TTGT-RBS0064_, Cm^R^_._ | This study |
| pP_MmP1_*-sfGFP* | pSEVA321 derivative, *sfGFP* gene cluster controlled by P_MmP1_ with lacO | Zhao et al., 2017^[3]^ |
| pP_MmP1_-*tnaA*-*fmo* | pSEVA321 derivative, *tnaA-fmo* gene cluster controlled by P_MmP1_ with lacO | This study |
| pP_cin_-*tnaA*-*fmo* | pSEVA321 derivative, *tnaA-fmo* gene cluster controlled by P_cin_ | This study |
| pP_lux_-*tnaA-fmo* | pSEVA321 derivative, *tnaA-fmo* gene cluster controlled by P_lux_ | This study |
| pP_porin58_-*luxR-luxI* | pSEVA341 derivative, *luxR-luxI* gene cluster controlled by P_porin58_, Km^R^ and Sp^R^. | This study |
| pP_porin194_-*luxR-luxI* | pSEVA341 derivative, *luxR-luxI* gene cluster controlled by P_porin194_, Km^R^ and Sp^R^. | This study |
| pP_porin68_-*luxR-luxI* | pSEVA341 derivative, *luxR-luxI* gene cluster controlled by P_porin68_, Km^R^ and Sp^R^. | This study |
| pP_porin226_-*luxR-luxI* | pSEVA341 derivative, *luxR-luxI* gene cluster controlled by P_porin226_, Km^R^ and Sp^R^. | This study |
| pP_lux-RBS0064_-*sf*GFP | pSEVA321 derivative, *sf*GFP controlled by P_lux-RBS0064_, Cm^R^ | This study |
| pP_lux-RBS2000_-*sf*GFP | pSEVA321 derivative, *sf*GFP controlled by P_lux-RBS2000_, Cm^R^ | This study |
| pP_lux-RBS0064_-*tnaA-fmo* | pSEVA321 derivative, *tnaA-fmo* gene cluster controlled by P_lux-RBS0064_, Cm^R^ | This study |
| pP_lux-RBS2000_-*tnaA-fmo* | pSEVA321 derivative, *tnaA-fmo* gene cluster controlled by P_lux-RBS2000_, Cm^R^ | This study |
| pP_porin221_-*dCas9*-P_porin58_-*sf*GFP | pSEVA321 derivative, *dCas9* controlled by P_porin221,_ *sf*GFP controlled by P_porin58_, Cm^R^_._ | This study |
| pP_J23117_-*dCas9*-P_MmP1_-sgRNA1 | pSEVA321 derivative, *dCas9* controlled by P_J23117_, sgRNA(*sf*GFP-site1) controlled by P_MmP1_, Cm^R^_._ | This study |
| pP_J23117_-*dCas9* | pSEVA321 derivative, *dCas9* controlled by P_J23117_, Cm^R^_._ | This study |
| pP_porin221_-*dCas9* | pSEVA321 derivative, *dCas9* controlled by P_porin221_, Cm^R^_._ | This study |
| pP_porin194_-*dCas9* | pSEVA321 derivative, *dCas9* controlled by P_porin194_, Cm^R^_._ | This study |
| pP_porin194_-*dCas9* | pSEVA321 derivative, *dCas9* controlled by P_porin287_, Cm^R^_._ | This study |
| pP_MmP1_-sgRNA2 | pSEVA341 derivative, sgRNA (*sf*GFP-site2) controlled by P_MmP1_, Km^R^ and Sp^R^. | This study |
| pP_MmP1_-sgRNA1 | pSEVA341 derivative, sgRNA (*sf*GFP-site1) controlled by P_MmP1_, Km^R^ and Sp^R^. | This study |
| pP_cin_-sgRNA1 | pSEVA341 derivative, sgRNA (*sf*GFP-site1) controlled by P_cin_, Km^R^ and Sp^R^. | This study |
| pP_cin_-sgRNA2 | pSEVA341 derivative, sgRNA (*sf*GFP-site2) controlled by P_cin_, Km^R^ and Sp^R^. | This study |
| pP_cin_-sgRNA1+2 | pSEVA341 derivative, sgRNA (*sf*GFP-site1) and sgRNA(*sf*GFP-site2) controlled by P_cin_, Km^R^ and Sp^R^. | This study |
| pP_lux_-sgRNA1 | pSEVA341 derivative, sgRNA (*sf*GFP-site1) controlled by P_lux_, Km^R^ and Sp^R^. | This study |
| pP_lux_-sgRNA2 | pSEVA341 derivative, sgRNA (*sf*GFP-site2) controlled by P_lux_, Km^R^ and Sp^R^. | This study |
| pP_lux_-sgRNA1+2 | pSEVA341 derivative, sgRNA (*sf*GFP-site1) and sgRNA(*sf*GFP-site2) controlled by P_lux_, Km^R^ and Sp^R^. | This study |
| pP_MmP1_-sgRNA1+2 | pSEVA341 derivative, sgRNA (*sf*GFP-site1) and sgRNA(*sf*GFP-site2) controlled by P_MmP1_, Km^R^ and Sp^R^. | This study |
| pP_cin_-*dCas9*-P_porin58_-*sf*GFP | pSEVA321 derivative, *dCas9* controlled by P_cin_ ,*sf*GFP controlled by P_porin58_, Cm^R^_._ | This study |
| pP_cin_-*dCas9-AAV*-P_porin58_-*sf*GFP | pSEVA321 derivative, *dCas9-AAV* controlled by P_cin_ ,*sf*GFP controlled by P_porin58_, Cm^R^_._ | This study |
| pP_cin_-*dCas9-AAV*-P_porin58_-*sf*GFP-*AAV* | pSEVA321 derivative, *dCas9-AAV* controlled by P_cin_ ,*sf*GFP*-AAV* controlled by P_porin58_, Cm^R^_._ | This study |
| pP_lux_-*dCas9*-P_porin58_-*sf*GFP | pSEVA321 derivative, *dCas9* controlled by P_lux_, *sf*GFP controlled by P_porin58_, Cm^R^_._ | This study |
| pP_lux-CATG-RBS2000_-*dCas9*-P_porin58_-*sf*GFP | pSEVA321 derivative, *dCas9* controlled by P_lux-CATG-RBS2000_, *sf*GFP controlled by P_porin58_, Cm^R^_._ | This study |
| pP_lux_-*dCas9-AAV*-P_porin58_-*sf*GFP | pSEVA321 derivative, *dCas9-AAV* controlled by P_lux_, *sf*GFP controlled by P_porin58_, Cm^R^_._ | This study |
| pP_lux-CATG-RBS2000_-*dCas9-AAV*-P_porin58_-*sf*GFP-*AAV* | pSEVA321 derivative, *dCas9-AAV* controlled by P_lux-CATG-RBS2000_, *sf*GFP-*AAV* controlled by P_porin58_, Cm^R^_._ | This study |
| pP_cin_-*dCas9-AAV* | pSEVA321 derivative, *dCas9-AAV* controlled by P_cin_, Cm^R^_._ | This study |
| pP_lux_-*dCas9-AAV* | pSEVA321 derivative, *dCas9-AAV* controlled by P_lux_, Cm^R^_._ | This study |
| pP_cin_-sgRNA*_mreB_* | pSEVA341 derivative, sgRNA*_mreB_* controlled by P_cin_, Km^R^ and Sp^R^. | This study |
| pP_lux_-sgRNA*_mreB_* | pSEVA341 derivative, sgRNA*_mreB_* controlled by P_lux_, Km^R^ and Sp^R^. | This study |
| pP_cin_-sgRNA*_prpC_* | pSEVA341 derivative, sgRNA*_prpC_* controlled by P_cin_, Km^R^ and Sp^R^ | This study |
| pP_lux_-sgRNA*_prpC_* | pSEVA341 derivative, sgRNA*_prpC_* controlled by P_lux_, Km^R^ and Sp^R^ | This study |
| pP_porin226-_*luxR-cinI­*-P_lux_-*mmP1* | pSEVA321 derivative, *luxR-cinI* controlled by P_porin226_, *mmP1* controlled by P_lux_, Cm^R^_._ | This study |
| pP_porin226_-*luxR-cinI­*-P_lux_-*mmP1*- P_MmP1 (without LacO)_-*sf*GFP | pSEVA321 derivative, *luxR-cinI* controlled by P_porin226_, *mmP1* controlled by P_lux_ and *sf*GFP controlled by P_MmP1 (without LacO)_, Cm^R^_._ | This study |
| pP_porin226_-*cinR-luxI­*-P_cin_-*mmP1* | pSEVA321 derivative, *cinR-luxI* controlled by P_porin226_, *mmP1* controlled by P_cin_, Cm^R^_._ | This study |
| pP_porin226_-*cinR-luxI­*-P_cin_-*mmP1*- P_MmP1 (without LacO)_-*sf*GFP | pSEVA321 derivative, *cinR-luxI* controlled by P_porin226_, *mmP1* controlled by P_cin_ and *sf*GFP controlled by P_MmP1 (without LacO)_, Cm^R^_._ | This study |
| p341-P_MmP1 (without LacO)_-*sfGFP* | pSEVA341 derivative, *sf*GFP controlled by P_MmP1 (without LacO)_, Km^R^ and Sp^R^. | This study |
| p321-P_MmP1 (without LacO)_-*sf*GFP | pSEVA321 derivative, *sf*GFP controlled by P_MmP1 (without LacO)_, Cm^R^ | This study |
| pP_J23119_-sgRNA*_mmP1_*-C1-donor | pSEVA341 derivative, P_J23119_-sgRNA (targeting Mmp1 RNA Polymerase expression module on genome), 1000bp P_cin-TACT-RBS0064_ donor, Km^R^ and Sp^R^. | This study |
| pP_J23119_-sgRNA*_mmP1_*-C2-donor | pSEVA341 derivative, P_J23119_-sgRNA (targeting Mmp1 RNA Polymerase expression module on genome), 1000bp P_cin-TTAA-RBS2000_ donor, Km^R^ and Sp^R^. | This study |
| pP_J23119_-sgRNA*_mmP1_*-L1-donor | pSEVA341 derivative, P_J23119_-sgRNA (targeting Mmp1 RNA Polymerase expression module on genome), 1000bp P_lux-CATG-RBS2000_ donor, Km^R^ and Sp^R^. | This study |
| pP_J23119_-sgRNA*_mmP1_*-L2-donor | pSEVA341 derivative, P_J23119_-sgRNA (targeting Mmp1 RNA Polymerase expression module on genome), 1000bp P_lux-TTGT-RBS0064_ donor, Km^R^ and Sp^R^. | This study |
| p321-P_MmP1(without LacO)_-*sod* | pSEVA321 derivative, *sod* controlled by P_MmP1 (without LacO)_, Cm^R^ | This study |

**Supplementary figures**


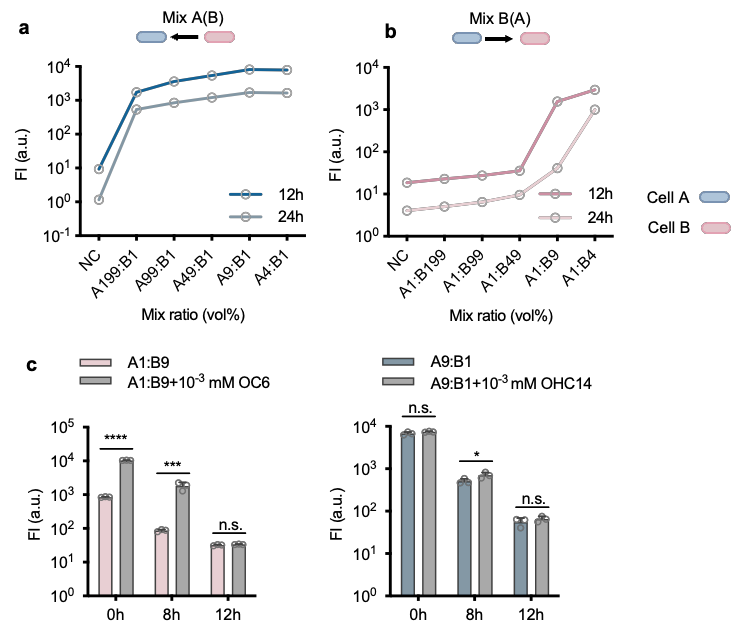


**Fig. S1 Studying the effects on sfGFP induction level based on different mix ratios of cells A and B.**

The seed cultures of recombinant cells A and B harboring *cinR-luxI* and *luxR-cinI* expression cassettes under the control of P_lacI_ promoter (see Fig. 1a) were initially mixed (mixed at 0 h) at different ratios, including 199:1, 99:1, 9:1 and 4:1, for 12 h cultivation. Parts **a** and **b** displayed two independent mixed cultures of cell A and B. Mixed A(B) in blue in the part **a**, a given ratio (vol%) of cell-B culture was sampled and added to cell-A culture; Mixed B(A) in pink in **b**, a given ratio (vol%) of cell-A culture was sampled and added to cell-B culture. Fluorescent intensity (FI, a.u.) of mixed culturing cells was measured by flow-cytometer (LSRFortessa, BD bioscience, USA). **c**. Studying the effects on induction levels of sfGFP by supplementing 10^-3^ mM OC6 and OC14 in A1:B9 (left) and A9:B1 (right) mixed cultural groups, respectively. Cells were mixed after 0, 8 and 12 h cultivation. Fluorescent intensity (FI, a.u.) of mixed culturing cells was measured by flow-cytometer (CytoFLEX, Beckman Coulter, USA). All cell cultures were obtained after 12 h and 24h mixed culture in shake flask and diluted about 100 times for FI recording and analysis. Cytometry data were analyzed by FlowJo software (v10.7) for quantitative determination of FI (a.u., mean value of captured cell counts in arbitrary unit). Data points are shown as mean ± SD of three replicates. *p* value: n.s., not significant; **p* < 0.0332; ***p* < 0.0021; ****p* < 0.0002; *****p* < 0.0001.


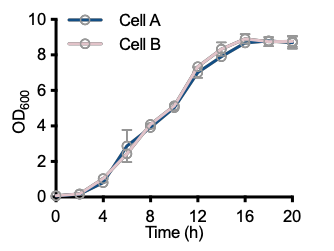


**Fig. S2 Cell growth characterization of recombinant cells A and B in 60LB.**

Cell growth curves of recombinant cells A and B (see Fig. 1a) were characterized independently by off-line measurement of OD_600_ in every 2 h (60LB, shake flask, without OHC14/OC6 supplementation). Data points are shown as mean ± SD of three replicates.


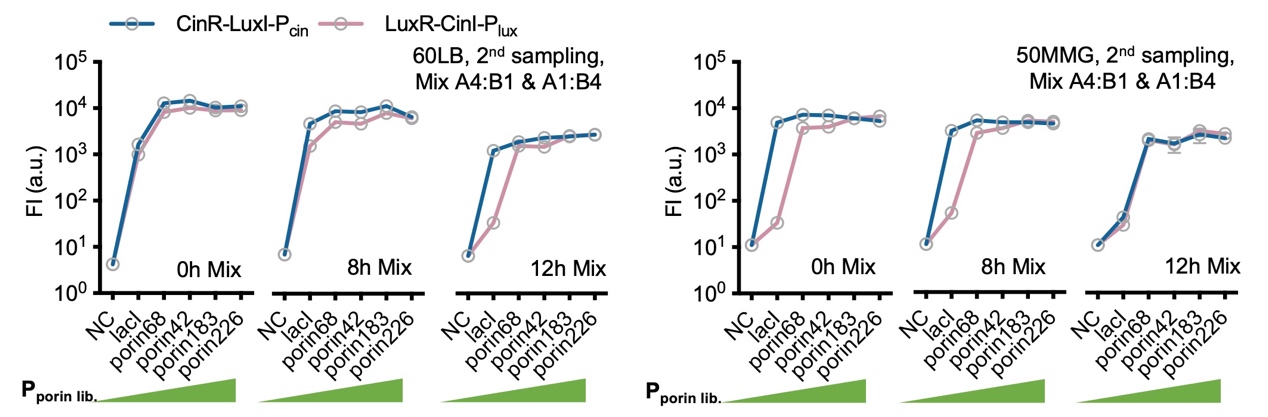


**Fig. S3 Expression assessment of optimized QS-based collaborative dynamic control systems at 2^nd^ sampling by flow-cytometer.**

Promoter engineering was employed to optimize the expression levels of *cinR-luxI* and *luxR-cinI* modules for enhanced dynamic induction level of sfGFP. Promoter mutants of different strength, including P_porin68_, P_porin42_, P_porin183_ and P_porin226_, were constructed in *Halomonas* TD and tested in contrast to the original design (*cinR-luxI* and *luxR-cinI* driven by P_lacI_). FI of mixed culturing cells grown in 60LB (left) and 50MMG (right) were measured by flow-cytometer. Cells were harvested at 2^nd^ sampling, which were sampled at 24 h (mixing at 8 h) and 36 h (mixing at 12 h), see Fig. 1a. NC, wild type TD. FI, Fluorescent intensity in arbitrary unit (a.u.). FI value of all cell cultures was measured by flow-cytometer after100-time dilution. Data points are shown as mean ± SD of three replicates.


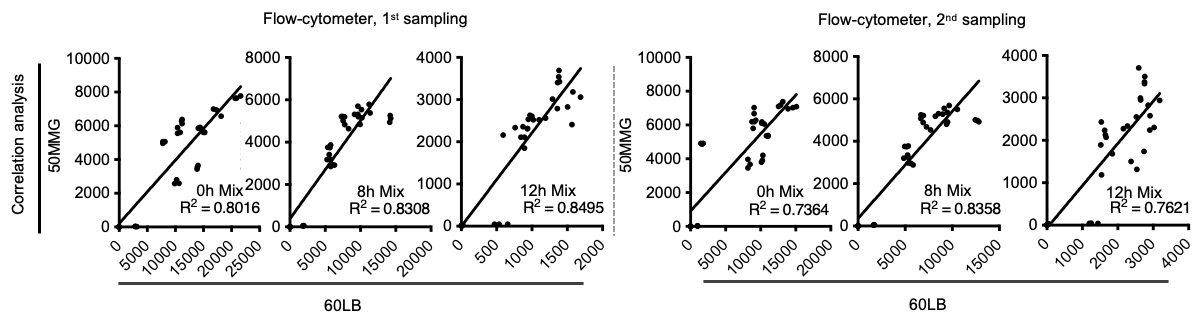


**Fig. S4 Correlation analysis of mixed cultural gene expression control across 60LB and 50MMG media via flow-cytometer.**

The FI values of mixed cultural cells A and B grown in 60LB medium (*x-axis*) was plotted against the groups grown in 50MMG (*y-axis*), which showed high linear correlation-ship with *R*^2^ > 0.7. FI of different mixed cultural groups of cells A and B, which contains different *cinR-luxI* and *luxR-cinI* expression modules driven by P_lacI_, P_porin68_, P_porin42_, P_porin183_ and P_porin226_ promoters, respectively, were analyzed by flow-cytometer (see Fig. S3). Left: 1^st^ sampling; Right: 2^nd^ sampling. FI value of all cell cultures was measured by flow-cytometer after100-time dilution. Data points are shown as mean ± SD of three replicates. FI, Fluorescence Intensity in arbitrary unit (a.u.).


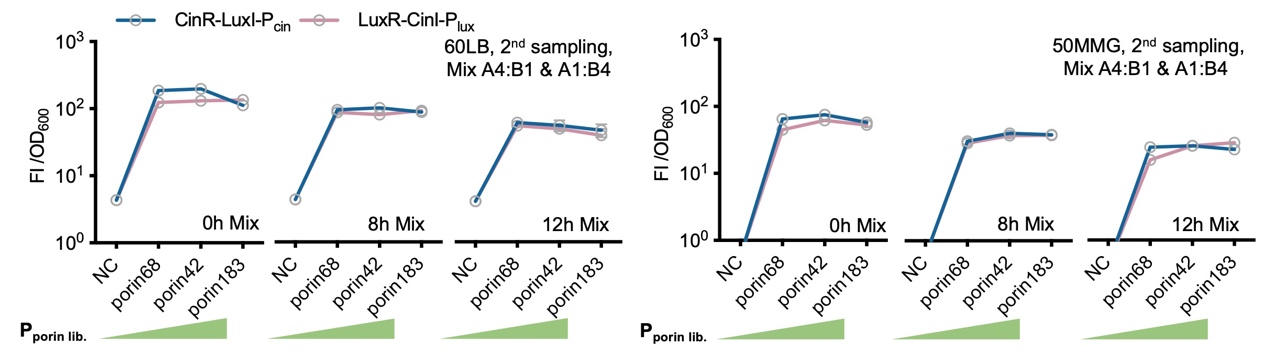


**Fig. S5 Expression assessment of optimized QS-based collaborative dynamic control systems at 2^nd^ sampling by microplate reader.**

The constructs containing optimized *cinR-luxI* and *luxR-cinI* modules driven by P_porin68_, P_porin42_ and P_porin183_ promoters, respectively, as well as negative control group (NC) from Fig. S3 were selected for further test in *Halomonas* TD by microplate reader. FI of mixed culturing cells grown in 60LB (left panel) and 50MMG (right panel) was measured by microplate reader. Cells were harvested at 2^nd^ sampling, which were sampled at 24 h (mixing at 8 h) and 36 h (mixing at 12 h), see Fig. 1a. NC, wild type TD. FI/OD_600_, normalized fluorescent intensity by dividing OD_600_. All cell cultures were obtained from 96-deep well plate culturing and diluted to 0.2-0.8 of OD_600_ for FI recording and analysis. Data points are shown as mean ± SD of three replicates.

**
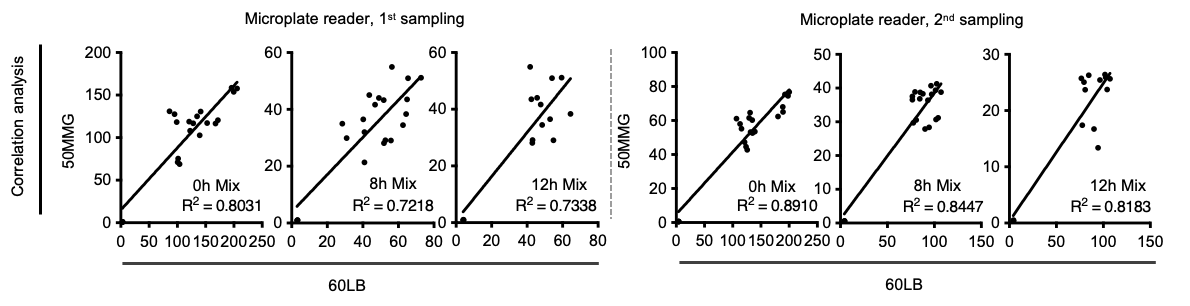
**

**Fig. S6 Correlation analysis of mixed cultural gene expression control across 60LB and 50MMG media via microplate reader.**

The FI values of mixed cultural cells A and B grown in 60LB medium (*x-axis*) was plotted against the groups grown in 50MMG (*y-axis*), which showed high linear correlation-ship with *R*^2^ > 0.7. Data points (FI) of different mixed cell cultural groups of cells A and B, which contains different *cinR-luxI* and *luxR-cinI* expression modules driven by P_porin68_, P_porin42_ and P_porin183_ promoters, respectively, were obtained by microplate reader measurement (see Fig. S5). Left: 1^st^ sampling; Right: 2^nd^ sampling. All cell cultures were obtained after 96-deep well plate culturing and diluted to 0.2-0.8 of OD_600_ for FI recording and analysis by microplate reader. Data points are shown as mean ± SD of three replicates. FI/OD_600_, normalized fluorescence intensity by dividing OD_600_.

**
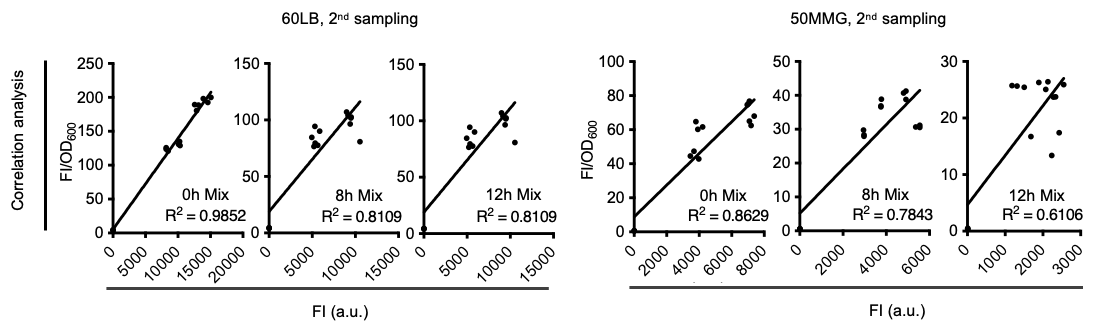
**

**Fig. S7 Correlation analysis of gene expression levels measured by flow-cytometer and microplate reader.**

The FI values of mixed cultural cells A and B grown in 60LB (left group) and 50MMG (right group) media were measured by flow-cytometer (*x-axis*) and plotted against the groups measured by microplate reader (*y-axis*) (2^nd^ sampling). These results were highly consistence with that of 1^st^ sampling from Fig. 1f (bottom panel). Data points are shown as mean ± SD of three replicates. FI: fluorescence intensity in arbitrary unit (a.u.), cells grown in shake flask measured by flow-cytometer after 100-time dilution; FI/OD_600_: normalized fluorescent intensity by dividing OD_600_, cells grown in 96-deep well plate were measured by microplate reader after customized dilution with OD_600_ reaching 0.2 to 0.8.


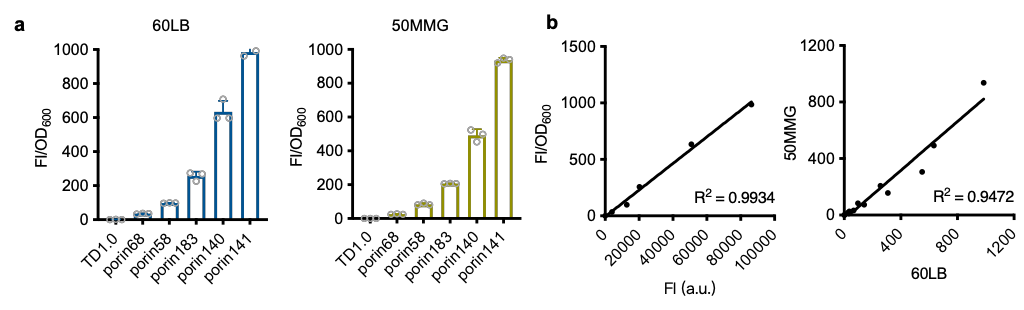


**Fig. S8 Correlation analysis of constitutive promoters across different media and FI measuring devices.**

**a,** Expression strength of P_porin68_, P_porin58_, P_porin183_, P_porin140_ and P_porin141_ promoters was characterized using sfGFP as reporter. Fluorescence of recombinant cells grown in 60LB (left) and 50MMG (right) media was measured by microplate reader. **b,** Left, linear fitting analysis of fluorescence intensity measured by flow-cytometer (*x-axis*) against microplate reader (*y-axis*); Right, linear fitting analysis of fluorescence intensity by recombinant cells grown in 60LB (*x-axis*) against in 50MMG (*y-axis*). FI, fluorescence intensity in arbitrary unit (a.u.) measured by flow-cytometer. FI/OD_600_, normalized fluorescent intensity by dividing OD_600_. Data points are shown as mean ± SD of three replicates.

**
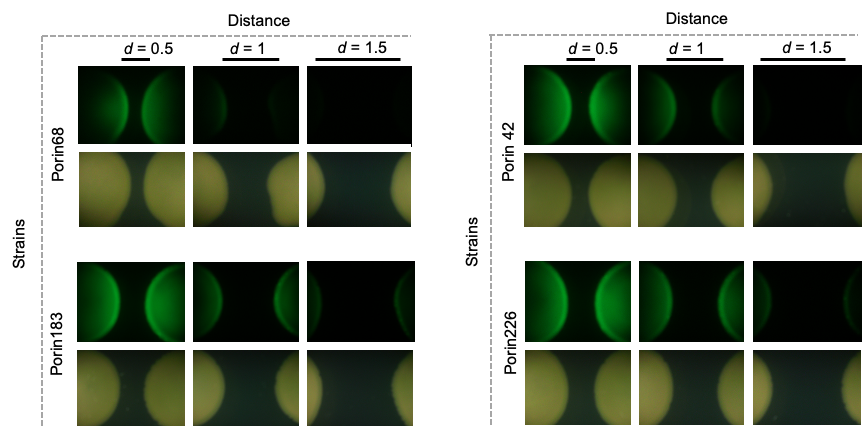
**

**Fig. S9** **Pattern formation characterization of cell-cell communication by recombinant cells A and B on agar plate**.

Bacterial lawns of cells A (left semicircle) and B (right semicircle) carrying different constructs of *cinR-luxI* and *luxR-cinI* expression modules from Fig. S3 were circularly spread on the same agar plates (60LB), respectively, with edge distance (*d*) setting at 0.5, 1.0 and 1.5 cm. After 24 h incubation, plates were photographed under dark (upper panel) and light (bottom panel) fields, respectively, to obtain visible patterns of cell-cell communication activity from each group. Fluorescence distribution of each semicircle under dark field indicates the effectiveness of cell-cell communication resulted from different expression strength of *cinR-luxI* and *luxR-cinI* modules, controlled by P_porin68_, P_porin183_, P_porin42_ and P_porin226_, and colonized distance of bacterial lawns (*d*).

**
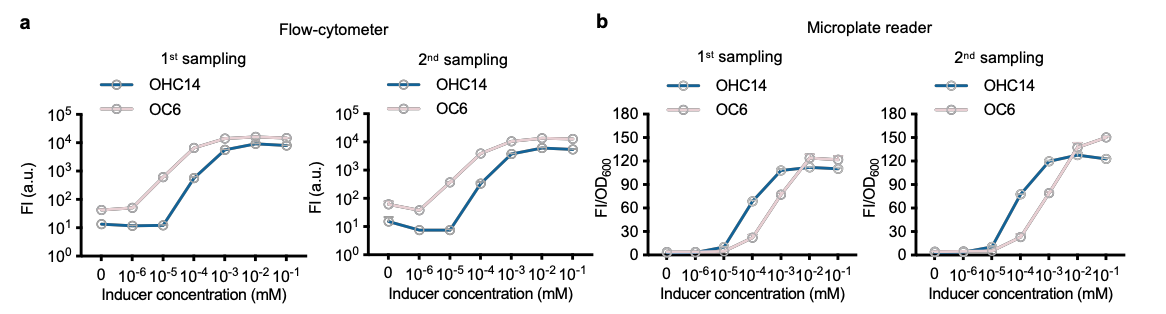
**

**Fig. S10** **Dose-response characterization for recombinant TY01 and TY02 strains harboring** ***sf*GFP expression modules driven by P_cin_/P_lux_ promoters**.

QS-based collaborative dynamic control circuits were constructed in recombinant cell A (TY01+ P_cin_) and B (TY02+ P_lux_) with chromosomal-carried *cinR-luxI* and *luxR-cinI* modules driven by P_porin226_, harboring plasmid-carried *sfGFP* expression modules driven by P_cin_ and P_lux_, respectively (see Fig. 3a). Dose-response curves of cells A and B grown in 60LB were characterized independently by supplementing different concentrations of OHC14 (cell A, line in blue) and OC6 (cell B, line in pink). Fluorescent intensity of recombinant cells was simultaneously analyzed by flow-cytometry (part **a**) and microplate reader (part **b**). FI: Fluorescence Intensity in arbitrary unit (a.u.), FI of recombinant cells was measured by flow-cytometer after 100-time dilution; FI/OD_600_: normalized fluorescent intensity by dividing OD_600_, FI of recombinant cells was measured by microplate reader after customized dilution with OD_600_ reaching 0.2 to 0.8. All data points are shown as mean ± SD of three replicates.

**
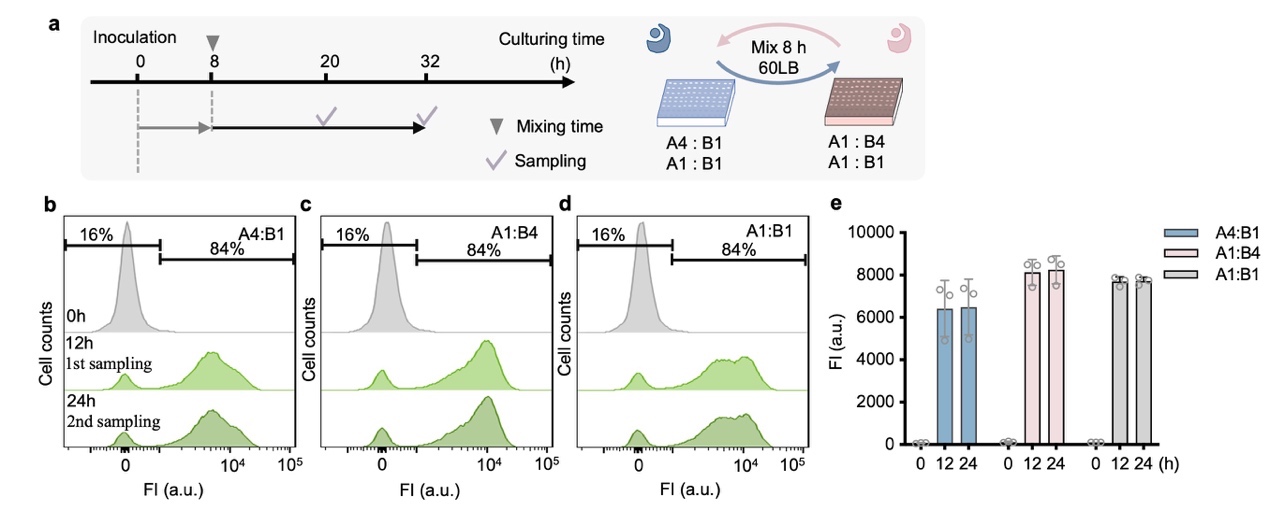
**

**Fig. S11 Characterization of QS-based collaborative dynamic control system by flow cytometer.**

**a**, Characterization workflow for testing the QS-based dynamic control performances by cell-A and -B mixed cultures grown in 96-deep well plate in single cell level. Cells A and B were mixed at 4:1 (A4:B1, A1:B4) and 1:1 (A1:B1) ratios, respectively, after 8 h pre-culture independently. Inverted triangles represent mixing time points of cells A and B, and ticks indicate the timing of cell culture sampling for FI measurement. **b-d**, Fluorescent intensity distribution of captured cell events from each mixed cultural group (A4:B1 in the part **b**; A1:B4 in the part **c**; A1:B1 in the part **d**) at 0 h, 12 h (1st sampling) and 24 h (2^nd^ sampling), respectively. The ratio of positive events with measured fluorescence in each group reached up to 84%. **e**, Mean values of FI of positive events from part **b-d**. Data points in **c** were shown as mean ± SD of three replicates.

**
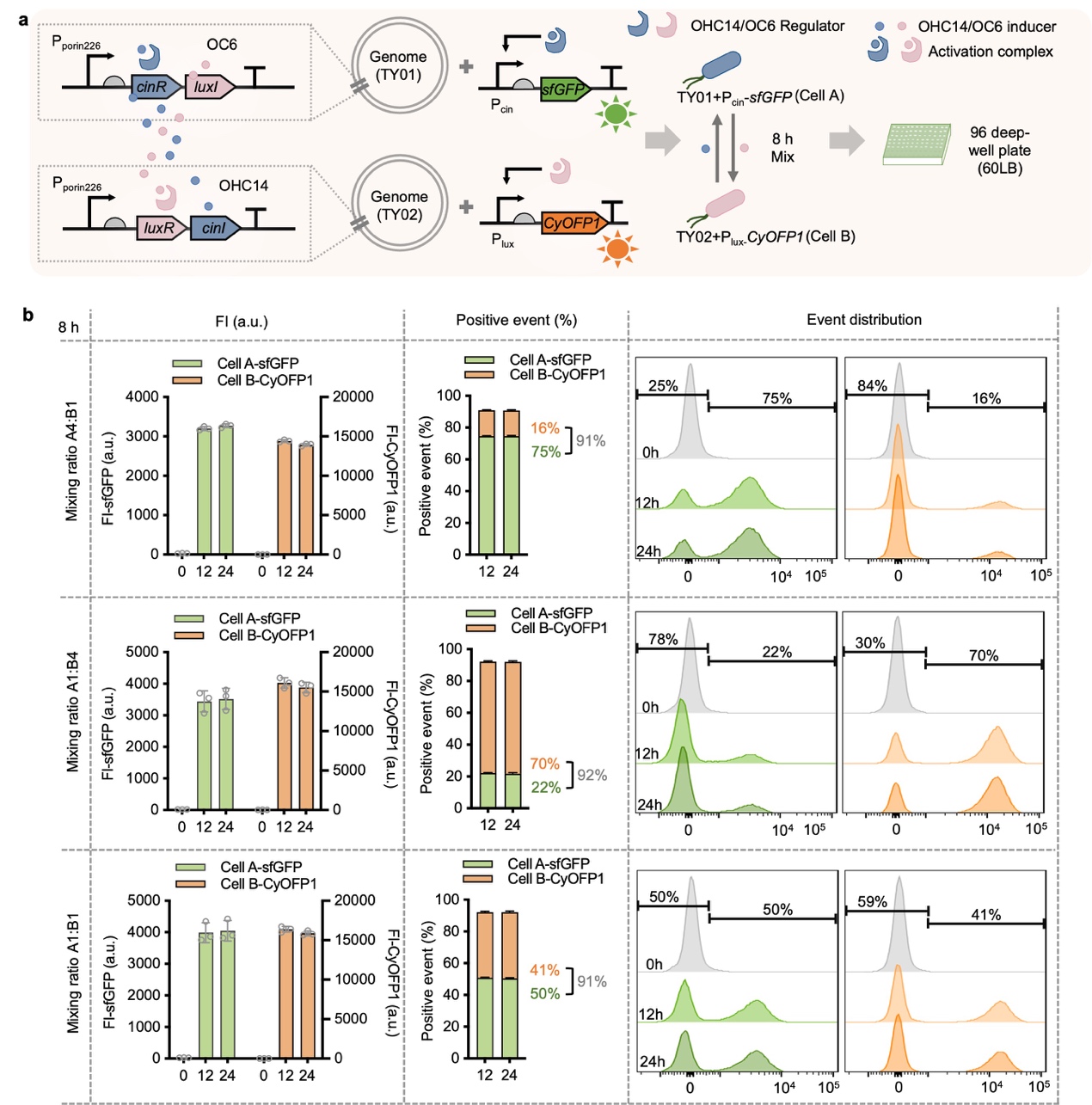
**

**Fig. S12 Characterizing the expression variance of recombinant cells A and B involved in QS-based collaborative dynamic control.**

**a**, Schematic design for expression variance characterization of cell A and B using two reporter fluorescent protein, sfGFP (cell A) and CyOFP1 (cell B) driven by P_cin_ and P_lux_ panel, respectively, derived from QS-based collaborative dynamic control system in Fig. 3a. **b**. Flow cytometer analysis of mixed culturing cells, including FI values of positive cell counts (left row), positive events of sfGFP and CyOFP1 and their ratios thereof (middle row), and fluorescence distribution patterns of captured cell counts (right row). Cells A and B were mixed at 4:1 (upper panel, A4:B1; middle panel, A1:B4) and 1:1 (, bottom panel, A1:B1) ratios, respectively, after 8 h pre-culture independently. Histograms and patterns in green indicate reporter sfGFP in cell A, and the ones in orange indicate reporter CyOFP1 cell B. Cell cultures of each group were sampled at 0 h (control), 12h (1^st^ sampling) and 24h (2^nd^ sampling) for FI measurement. Data points of histograms in **b** are shown as mean ± SD of three replicates.

**
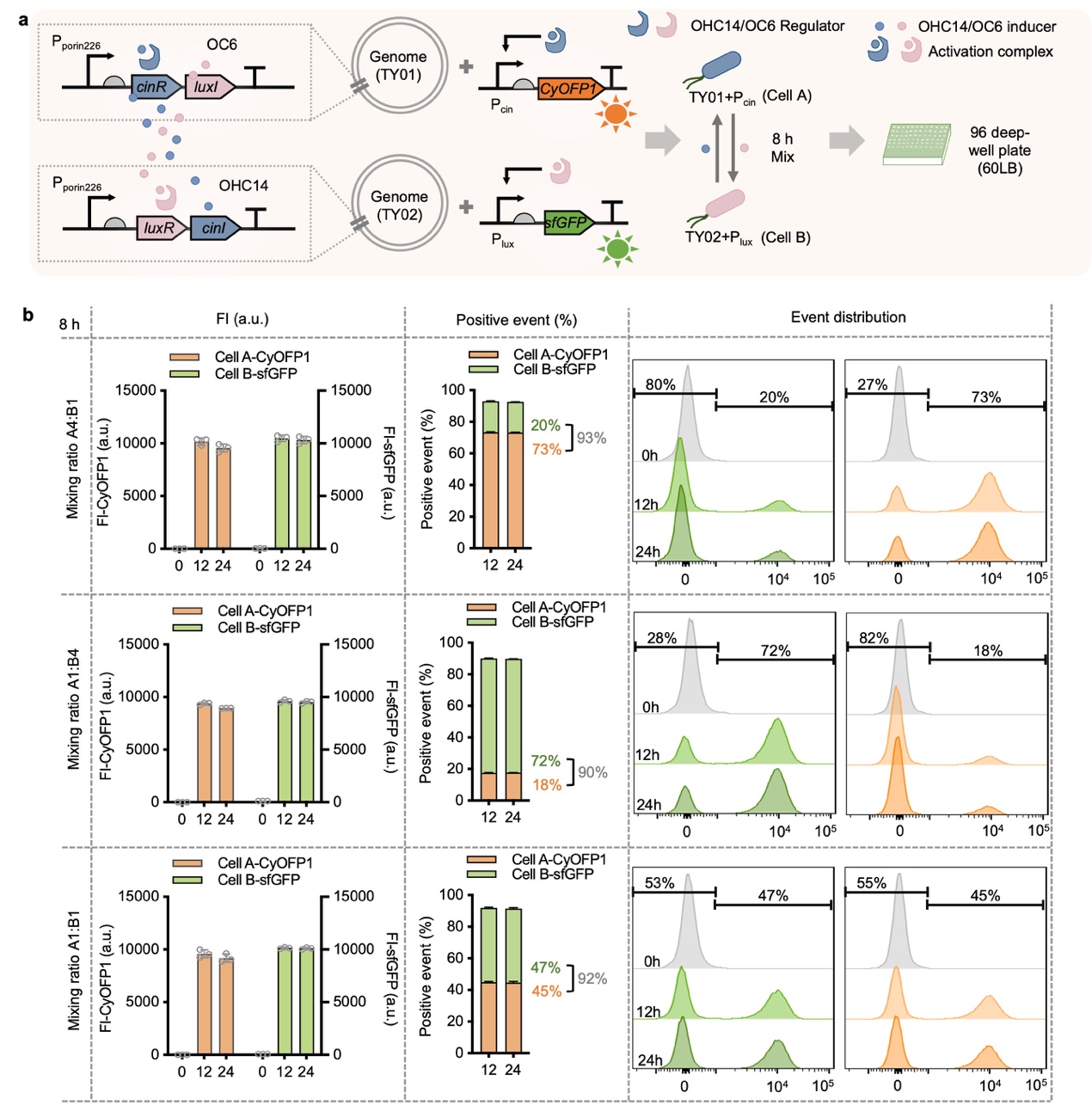
**

**Fig. S13 Characterizing the expression variance of cell A and B involved in QS-based collaborative dynamic control.**

**a**, Schematic design for expression variance characterization of cell A and B using two reporter fluorescent protein, CyOFP1 (cell A) and sfGFP (cell B) driven by P_cin_ and P_lux_ panel, respectively, compared to the design from Fig. S12. **b**. Flow cytometer analysis of mixed culturing cells, including FI values of positive cell counts (left row), positive events of sfGFP and CyOFP1 and their ratios thereof (middle row), and fluorescence distribution patterns of captured cell counts (right row). Cells A and B were mixed at 4:1 (upper panel, A4:B1; middle panel, A1:B4) and 1:1 (bottom panel, A1:B1) ratios, respectively, after 8 h pre-culture independently. Histograms and patterns in green indicate reporter sfGFP in cell B, and the ones in orange indicate reporter CyOFP1 cell A. Cell cultures of each group were sampled at 0 h (control), 12h (1^st^ sampling) and 24h (2^nd^ sampling) for FI measurement. Data points of histograms in **b** are shown as mean ± SD of three replicates.

**
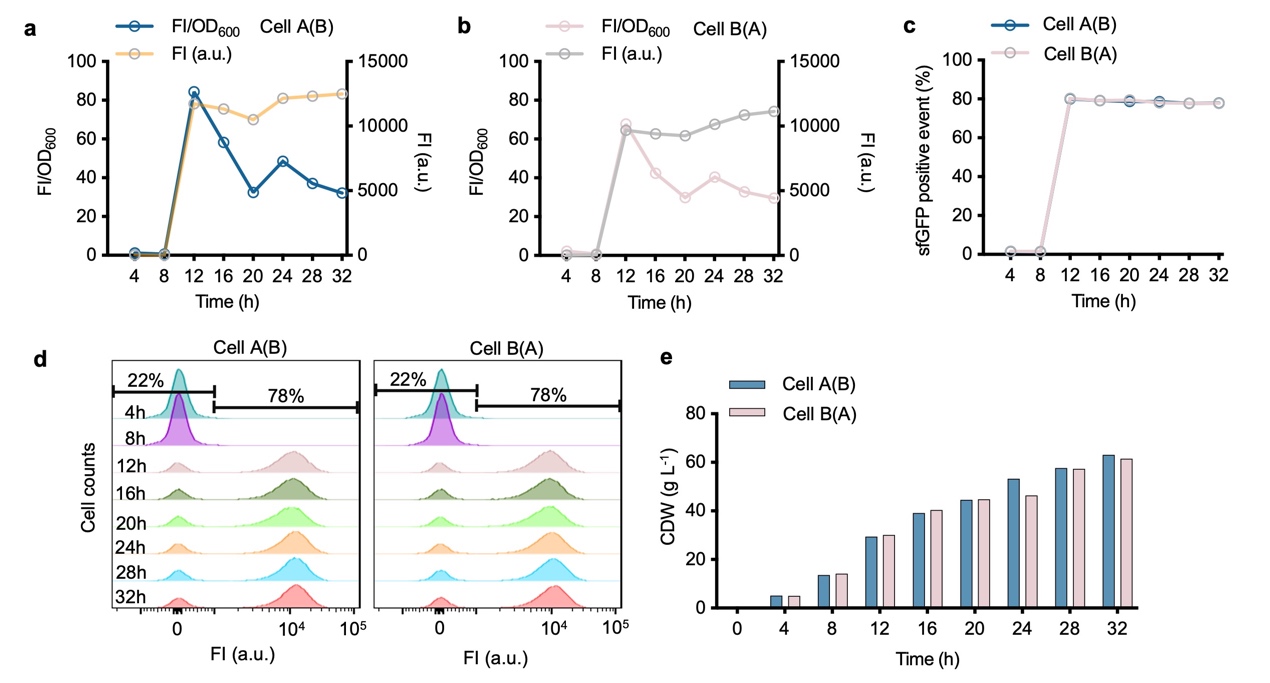
**

**Fig. S14 Comparative analysis of sfGFP expression determined by flow-cytometer against microplate reader during fed-batch study.**

Pre-cultures of cell A and B were 1:1 mixed (vol%) after 8 h independent growth in two 7-L bioreactors, respectively. **a** and **b**, time-course FI values of mixed culturing cells from two independent bioreactors were simultaneously measured by flow cytometer (FI, a.u.) and microplate reader (FI/OD_600_, diluted to 0.2-0.8 of OD_600_). **c**, percentage of positive events with sfGFP expression from part **a** and **b**. **d**, fluorescence distribution of captured cell events analyzed by flow cytometer. **e**, Cell dry weight of mixed cultures in every 4 h during the fed-batch study.

**
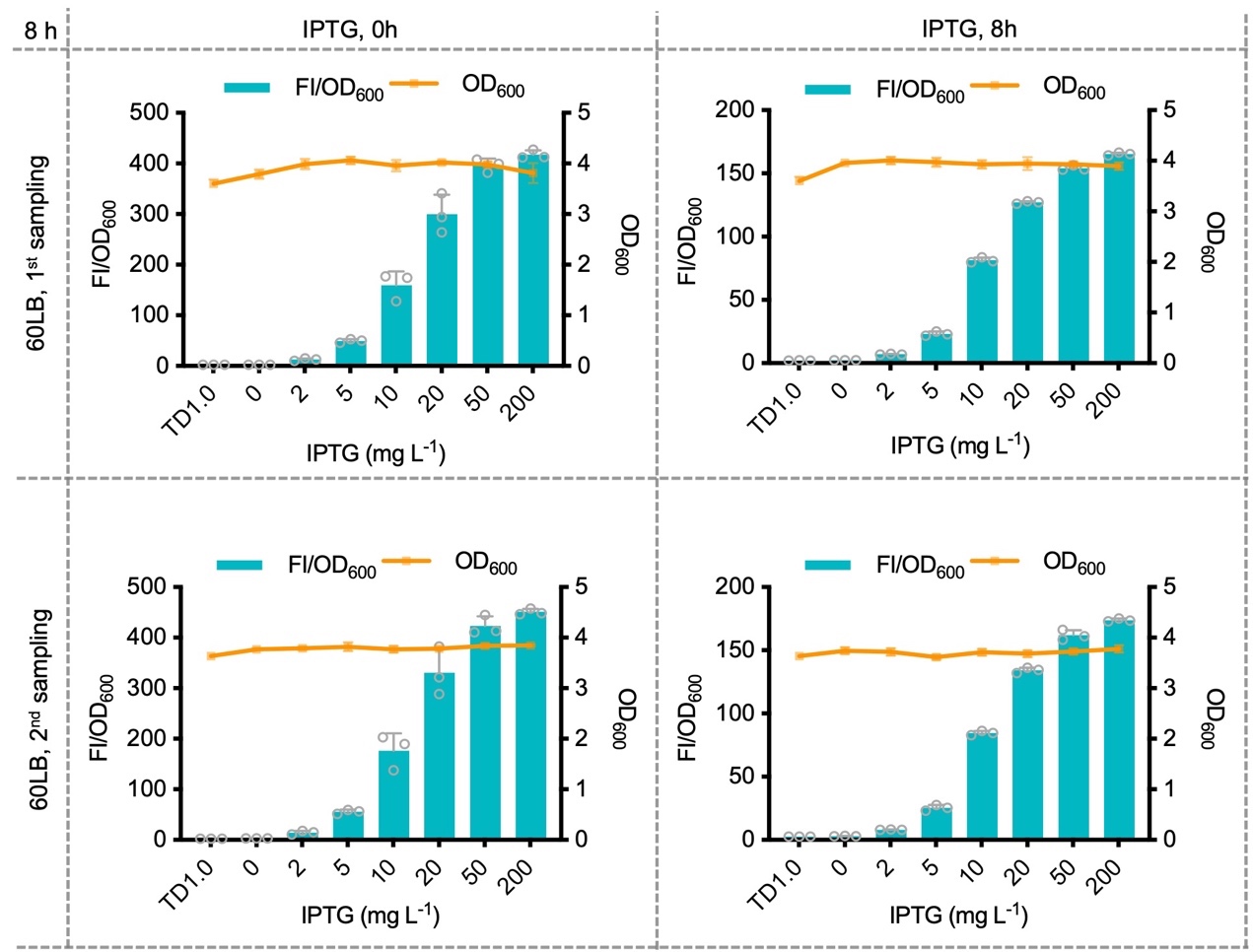
**

**Fig. S15 Dose-response characterization of IPTG-induced system (MmP1).**

Recombinant cells harboring IPTG-induced MmP1 system (using sfGFP as reporter) grown in 60LB medium in a 96-deep well plate. Different IPTG concentrations, including 0, 2, 5, 10, 20, 50 and 200 mg L^-1^, were supplemented at 0 h (left row) and 8 h (right row), respectively, for induced expression of sfGFP. Cell cultures were sampled for FI (histogram, FI/OD_600_) and OD_600_ (line) measurement after 12 h (upper panel, 1^st^ sampling) and 24 h (bottom panel, 2^nd^ sampling) growth, respectively. Data points are shown as mean ± SD of three replicates.

**
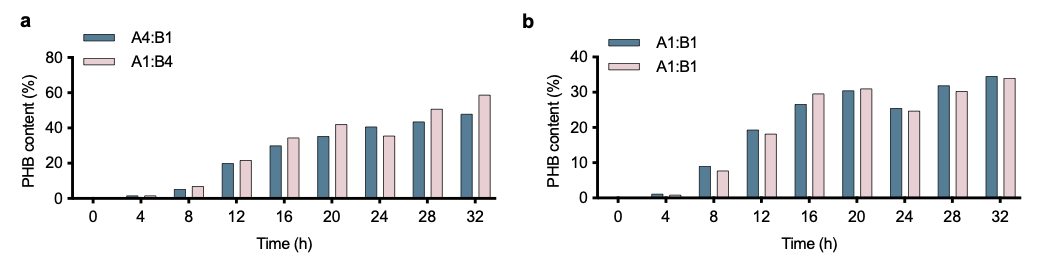
**

**Fig. S16** **PHB accumulation of fed-batch study by recombinant strains TY01 and TY02 harboring sfGFP expression module.**

Fed-batch study of recombinant cells A and B from Supplementary Fig. 10 (also see Fig. 3e) was conducted in a 7-L bioreactor. Cells A and B were independently pre-cultured for 8 h after inoculation and mixed at 4:1 (part **a**, A4:B1 group in blue and A1:B4 group in pink) and 1:1 (part **b**) ratio. Cell dry weight (see Fig. 3e) and PHB content were analyzed in every 4 h. Fed-batch fermentation of each mixed culture group (A4:B1/A1:B4 and 1:1) was conducted in two parallel 7-L bioreactors.


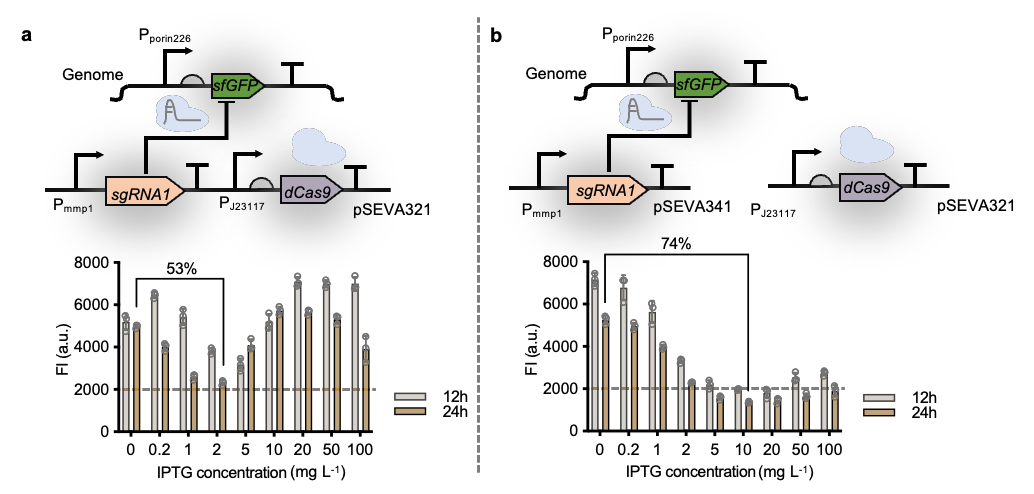


**Fig. S17** **Repression efficiency assessment of prototyping CRISPRi system**.

Two expression units involved in CRISPRi system, including sgRNA1 driven by P_MmP1_ induced by IPTG and dCas9 driven by P_J23117_, were constructed in single- (part **a**) and double- (part **b**) plasmid system, respectively, for repression efficiency assessment. A constitutive expression module of *sf*GFP controlled by P_porin226_ was integrated on the genome locus G7 in start host *Halomonas* TD1.0, namely TD-GFP, and used as donor cell for CRIPSRi-based repression test. Repression efficiency assessment was carried out by recombinant cell TD-GFP harboring constructs from part **a** and part **b**, respectively, grown in 50MMG in the presence of different concentrations of IPTG. FI value of cell cultures after 12 h and 24 h growth was measured and analyzed by flow-cytometer. FI, Fluorescence Intensity in arbitrary unit (a.u.). Data points are shown as mean ± SD of three replicates.


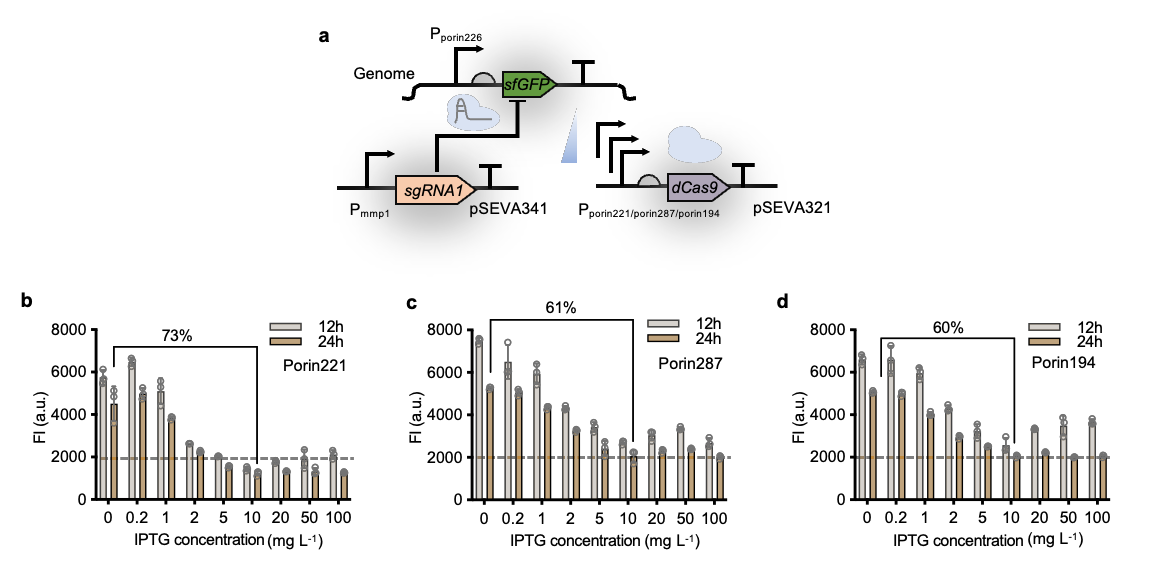


**Fig. S18 Repression efficiency analysis of CRISPRi system under different expression levels of dCas9.**

**a,** Three promoters (P_porin221_ in part **b**, P_porin287_ in part **c**, P_porin194_ in part **d**) of different strength were used to control the expression of *d*Cas9 based on the double-plasmid design in Fig. S17. Repression efficiency assessment was carried out by recombinant cell TD-GFP harboring different CRISPRi systems grown in 50MMG in the presence of different concentrations of IPTG. FI value of cell cultures after 12 h and 24 h growth was measured and analyzed by flow-cytometer. FI, Fluorescence Intensity in arbitrary unit (a.u.). Data points are shown as mean ± SD of three replicates.


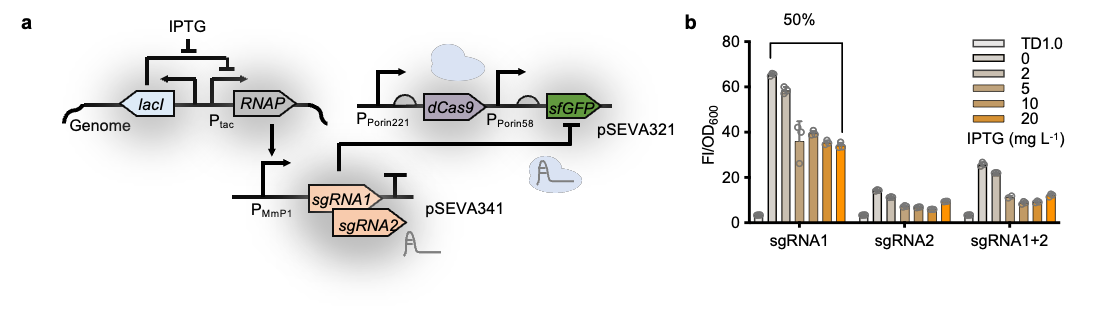


**Fig. S19 Debugging of sgRNA design for effective CRISPRi repression in recombinant *Halomonas* TD.**

**a,** Two sfGFP-targeted sgRNA sequences, sgRNA1 and sgRNA2, and their combination (sgRNA1+2) were designed for CRISPRi repression test. In contrast to previous design of double-plasmid system in Fig. S18, *sfGFP* controlled by P_porin58_ was constructed together with *dCas9* cassettes on the same plasmid. **b,** The repression performances of three sgRNA combinations (sgRNA1, sgRNA2, sgRNA1+2) of different transcribed levels were characterized in 50MMG medium after 12h culturing in the presence of 0, 2, 5, 10 and 20 mg L^-1^ IPTG, respectively (data obtained after 24h culturing was shown in Fig. 4a). FI values were measured by microplate reader after cell cultures diluted to 0.2-0.8 of OD_600_ and normalized by dividing OD_600_ value. All data points are shown as mean ± SD of three replicates.

**
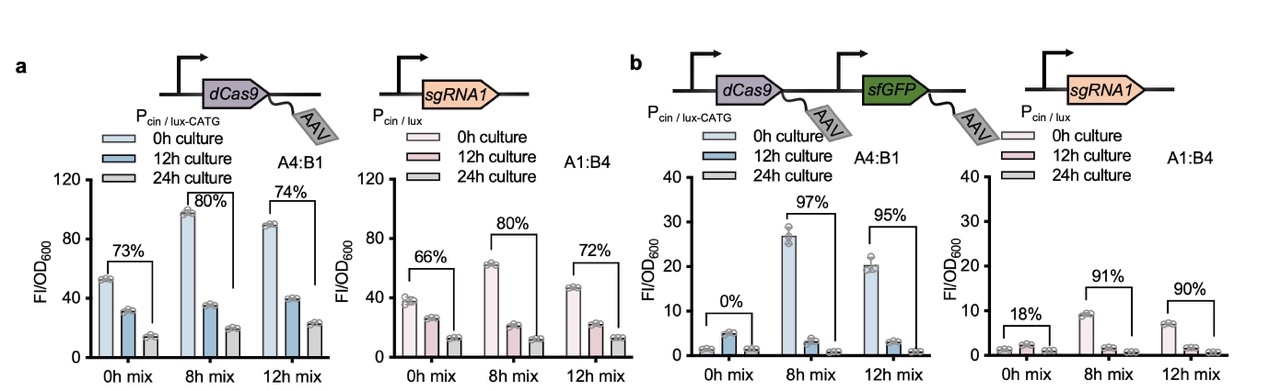
**

**Fig.** **20 Assessment of repression efficiency by mixing cells A and B after 0 h (0h mix), 8 h (8h mix) and 12 h (12h mix) pre-culture independently**.

The *sf*GFP expression levels of pre-cultured cells A and B derived from Fig. 4d right before mixing were used as control group (0h culture) against the repressed groups after 12 h (12h culture) and 24 h (24h culture) mixing growth. **a**, A degradation tag AAV was added to the C-terminal of dCas9 controlled by Pcin and P_lux_ promoter mutant (P_lux-CATG_) with lower leakiness to study the effect on repression activity in recombinant cell B (TY02), respectively. **b**, A degradation tag AAV was added to the C-terminal of sfGFP to assess the effect on basal expression and dynamic repression of sfGFP based on the design in part **a.** FI values were measured by microplate reader after cell cultures diluted to 0.2-0.8 of OD_600_ and normalized by dividing OD_600_ (FI/OD_600)_. Data points are shown as mean ± SD of three replicates.


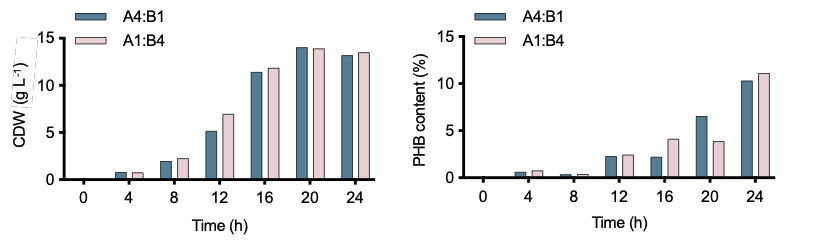


**Fig. S21** **PHB accumulation of fed-batch study by recombinant strains TC01 and TC02 harboring optimized QS-based collaborative CRISPRi system.**

Fed-batch study of recombinant cells TC01 and TC02 from Fig. 4g was conducted in a 7-L bioreactor. Independent pre-culture of cells A and B were mixed at 4:1 (left, A4:B1 group in blue and A1:B4 group in pink) after 8 h of inoculation, forming two mixed culture groups. Cell dry weight (left) and PHB content (right) were analyzed in every 4 h. Fed-batch fermentation was conducted in two parallel 7-L bioreactors.

**
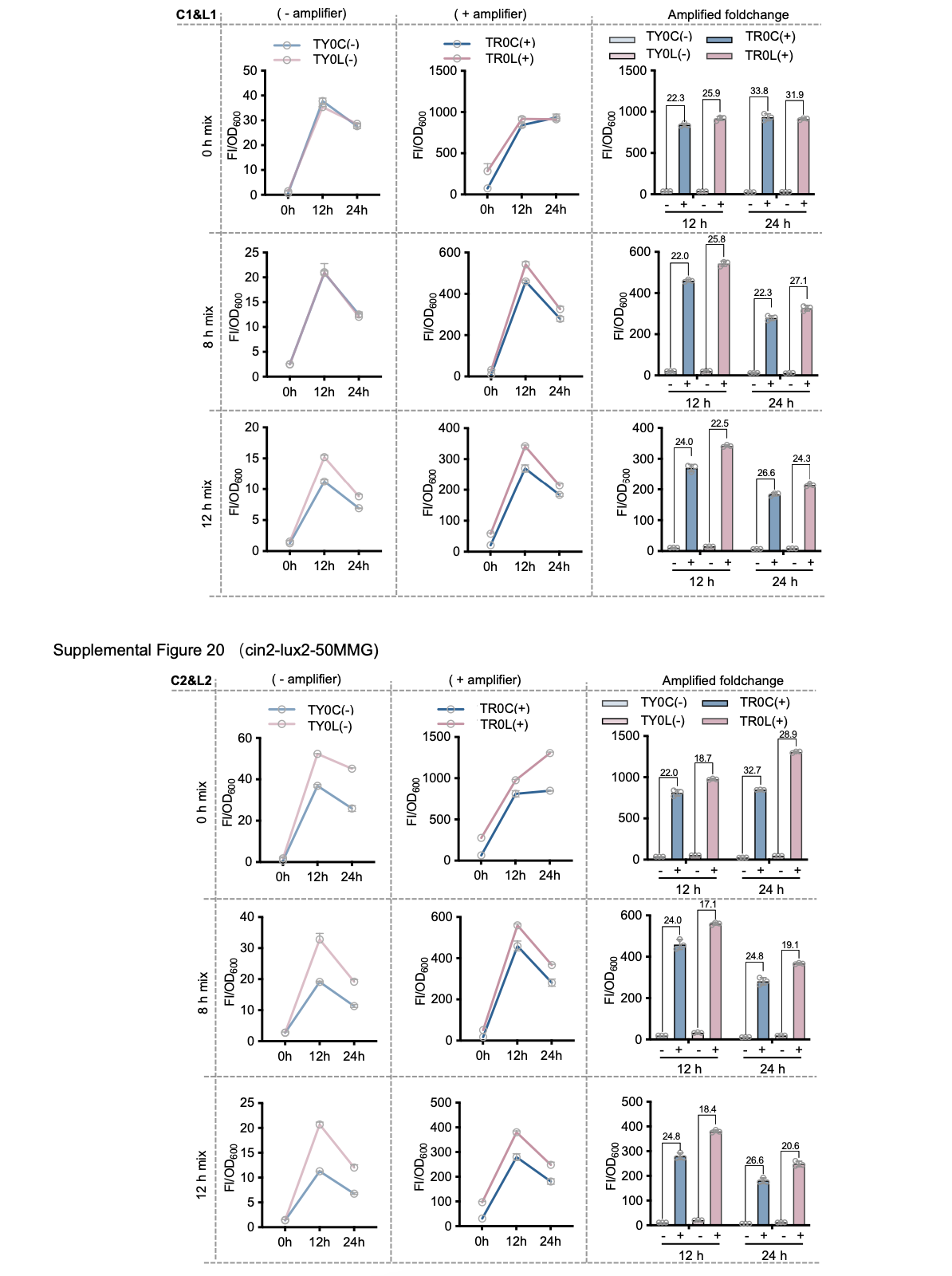
**

**Fig. S22 Robustness test of QS-based collaborative amplifier (C1&L1) in 50MMG.**

In contrast to the assessment in 60LB medium from Fig. 5d, the amplifier design of C1&L1 (see Fig. 5a) was characterized in 50MMG by mixing cells A, TR0C(+), and cell B, TR0L(+), after 0 h (upper panel), 8 h (middle panel) and 12 h (bottom panel) independent growth in 96 deep-well plate. *sf*GF*P* under the control of P_cin_ (C1+RBS0064) and P_lux_ (L1+RBS2000) were constructed in strains TY01 and TY02 as controls (without amplifier), namely TY0C(-) and TY0L(-), respectively, for amplified foldchange calculation. Normalized FI (FI/OD_600_) of QS-based collaborative dynamic control circuits without (‘-’) and with (‘+’) amplifier were shown for comparative analysis. And ‘0 h’ in *x-axis* indicates the ‘OFF-state’ right before cell mixing (control group) compared to the ‘ON-state’ after 12 and 24 h mixed culture. Histograms with amplified foldchanges were shown in the right row. FI values were measured by microplate reader after cell cultures diluted to 0.2-0.8 of OD_600_ and normalized by dividing OD_600_ value. All data points are shown as mean ± SD of three replicates.

**
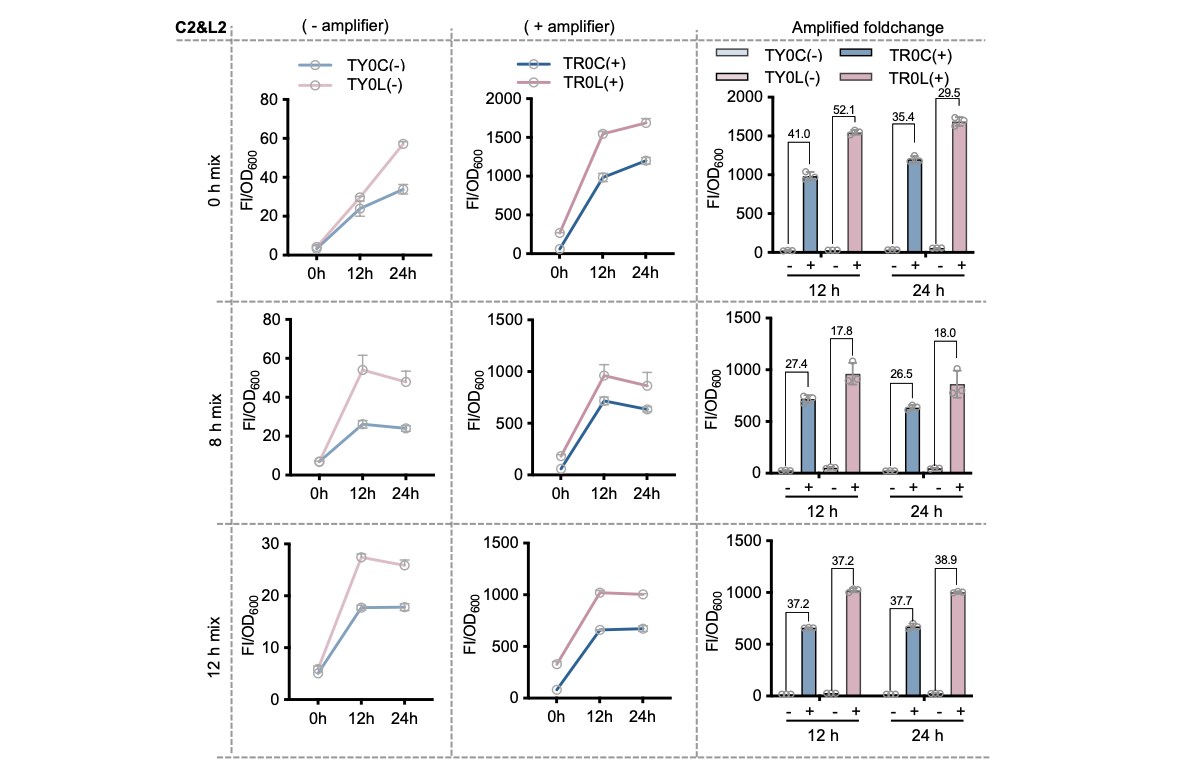
**

**Fig.** **S23 Comparative analysis of QS-based collaborative amplifier by tuning the induction level of MmP1 RNAP.**

Assessment of dynamic activation performance of C2&L2 group (see Fig. 5a) by mixing cells A, TR0C(+), and cell B, TR0L(+), after 0 h (upper panel), 8 h (middle panel) and 12 h (bottom panel) independent growth in 96 deep-well plate in 60LB. The sfGFP under the control of P_cin_ (C2+RBS2000) and P_lux_ (L2+RBS0064) were constructed in strains TY01 and TY02 as controls (without amplifier), namely TY0C(-) and TY0L(-), respectively, for amplified foldchange calculation. Normalized FI/OD_600_ of QS-based collaborative dynamic control circuits without (‘-’) and with (‘+’) amplifier were shown for comparative analysis. And ‘0 h’ in *x-axis* indicates the ‘OFF-state’ right before cell mixing (control group) compared to the ‘ON-state’ after 12 and 24 h mixed culture. Histograms with amplified foldchanges were showed in the right row. FI values were measured by microplate reader after cell cultures diluted to 0.2-0.8 of OD_600_ and normalized by dividing OD_600_ value. All data points are shown as mean ± SD of three replicates.


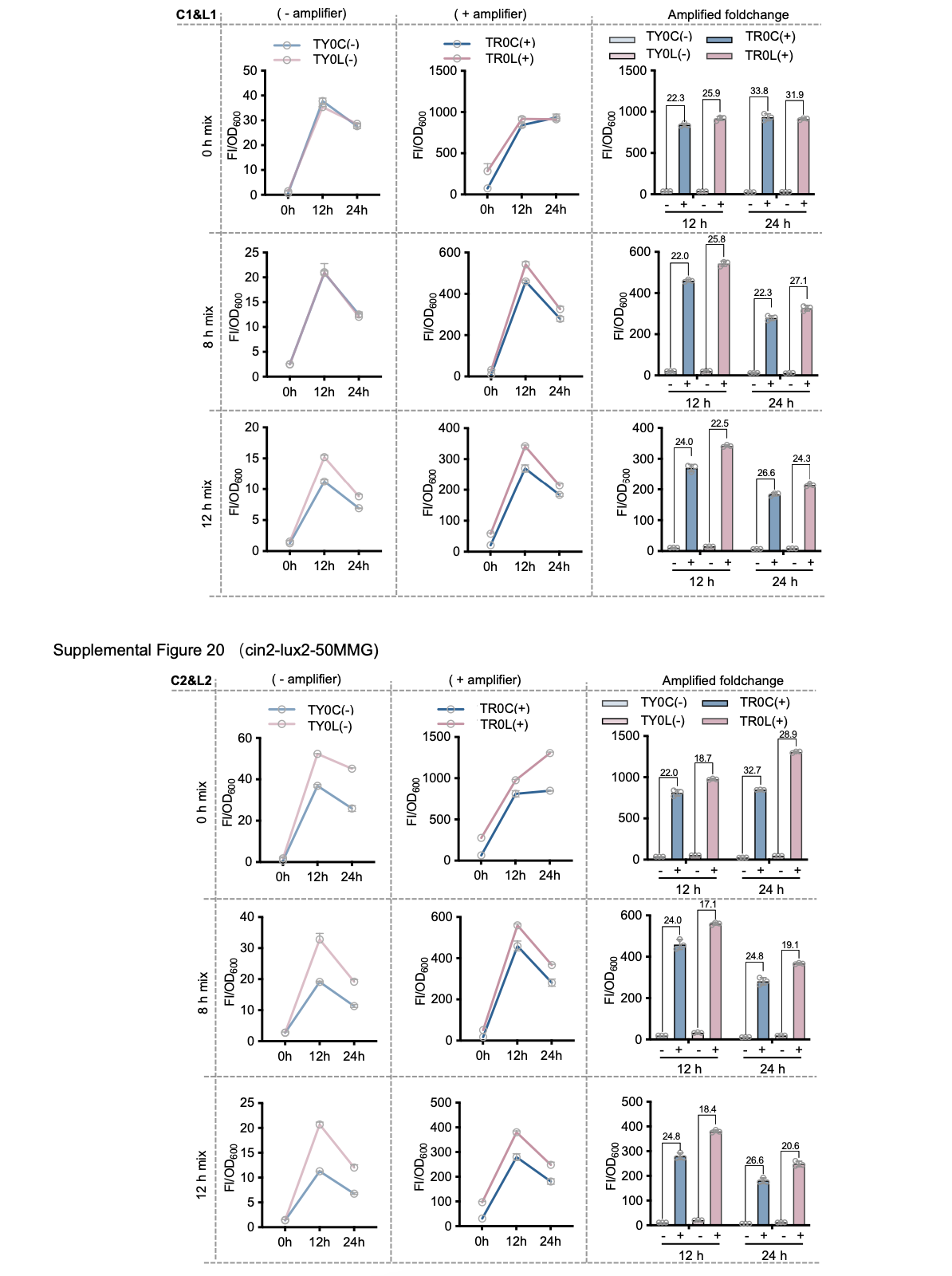


**Fig. S24 Robustness test of QS-based collaborative amplifier (C2&L2) in 50MMG.**

In contrast to the assessment in 60LB medium from Fig. S23, the amplifier design of C2&L2 (see Fig. 5a) was characterized in 50MMG by mixing cell A, TR0C(+), and cell B TR0L(+), after 0 h (upper panel), 8 h (middle panel) and 12 h (bottom panel) independent growth in 96 deep-well plate. *sf*GFP under the control of P_cin_ (C2+RBS2000) and P_lux_ (L2+RBS0064) were constructed in strains TY01 and TY02 as controls (without amplifier), namely TY0C(-) and TY0L(-), respectively, for amplified foldchange calculation. Normalized FI (FI/OD_600_) of QS-based collaborative dynamic control circuits without (‘-’) and with (‘+’) amplifier were shown for comparative analysis. And ‘0 h’ in *x-axis* indicates the ‘OFF-state’ right before cell mixing (control group) compared to the ‘ON-state’ after 12 and 24 h mixed culture. Histograms with amplified foldchanges were showed in the right row. FI values were measured by microplate reader after cell cultures diluted to 0.2-0.8 of OD_600_ and normalized by dividing OD_600_ value. All data points are shown as mean ± SD of three replicates.

**
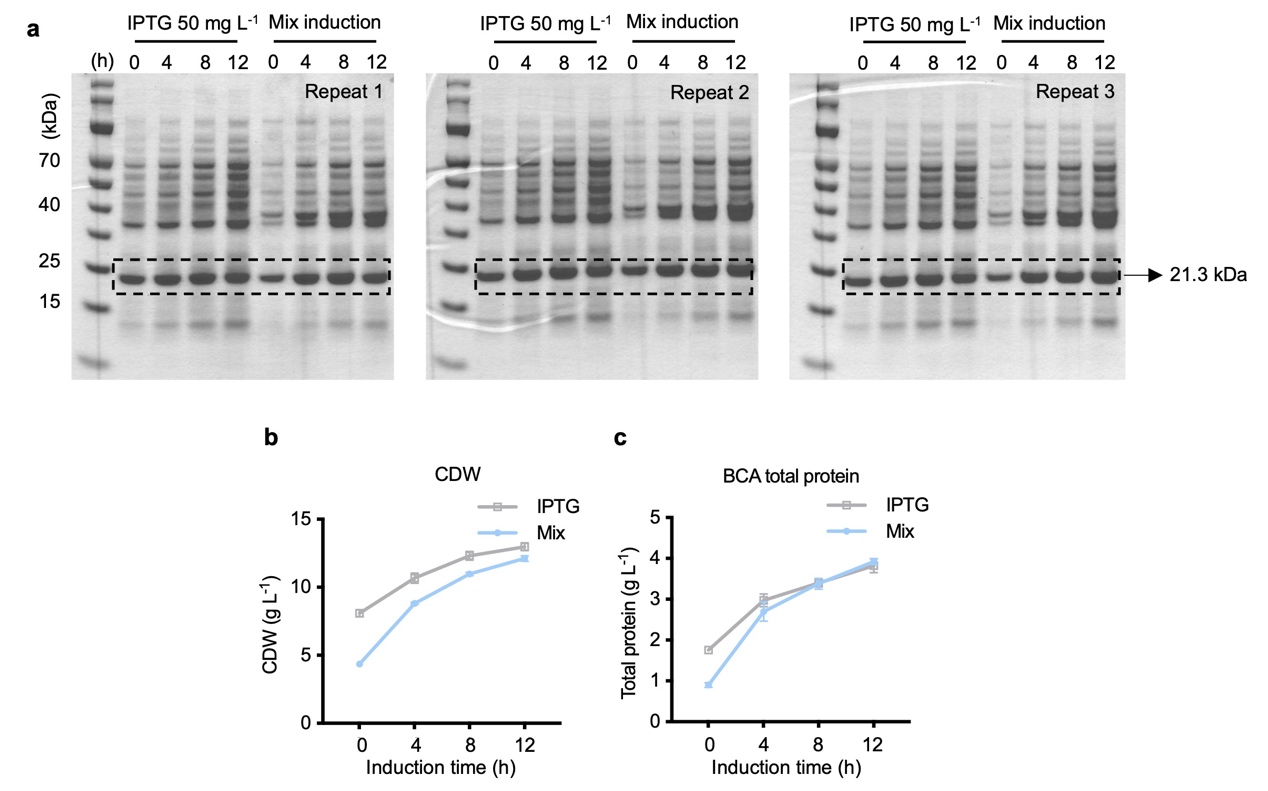
**

**Fig. S25 Comparative analysis of SOD production by engineered *Halomonas* TD using different inducible systems.**

**a**. SDS-PAGE analysis (triplicates) of intracellular soluble SOD by engineered *Halomonas* TD using IPTG-induced system (50 mg L^-1^) and QS-based collaborative amplifier system (mixed at 8 h), respectively, grown in 50MM medium supplemented with 30 g L^-1^ glucose and 5 g L^-1^ urea. The induction performances at 0 h, 4 h, 8 h and 12 h were assessed by measuring the SOD titer and protein ratio of SOD (Fig. 5g), the CDW (part **b**) and total protein (part **c**). Data points in **b** and **c** are shown as mean ± SD of three replicates.

**
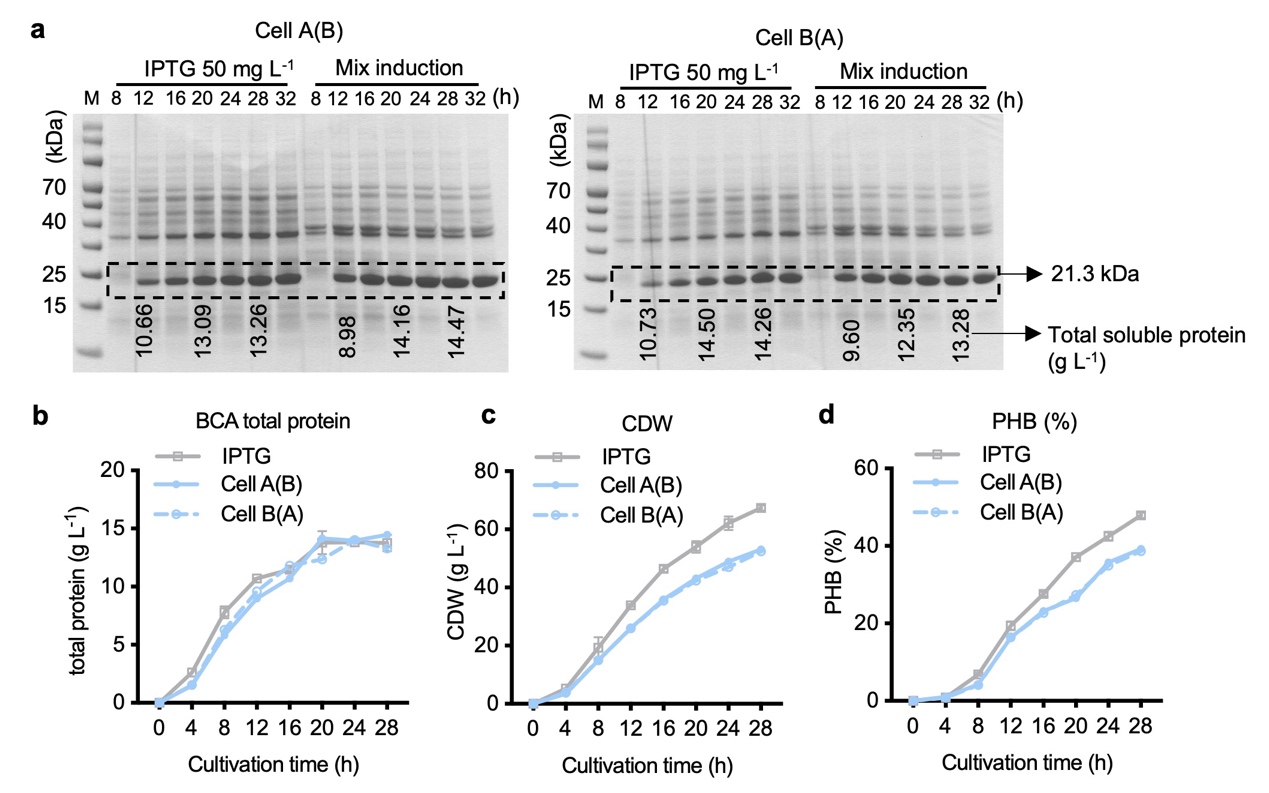
**

**Fig. S26 Fed-batch study for SOD production using QS-based collaborative amplifier system.**

**a**. SDS-PAGE analysis for SOD synthesis controlled by IPTG-induced system (50 mg L^-1^) and QS-based collaborative amplifier (mixed at 8 h), respectively, conducted in two parallel 7-L bioreactors. The time-course expression levels, protein ratio and enzyme activity of SOD (Figs. 5h-5i), as well as total protein (**b**), CDW (**c**) and PHB content (**d**), were analyzed in every 4 h after mixed culturing. Data points are shown as mean ± SD of two replicates for IPTG-induced group.

**
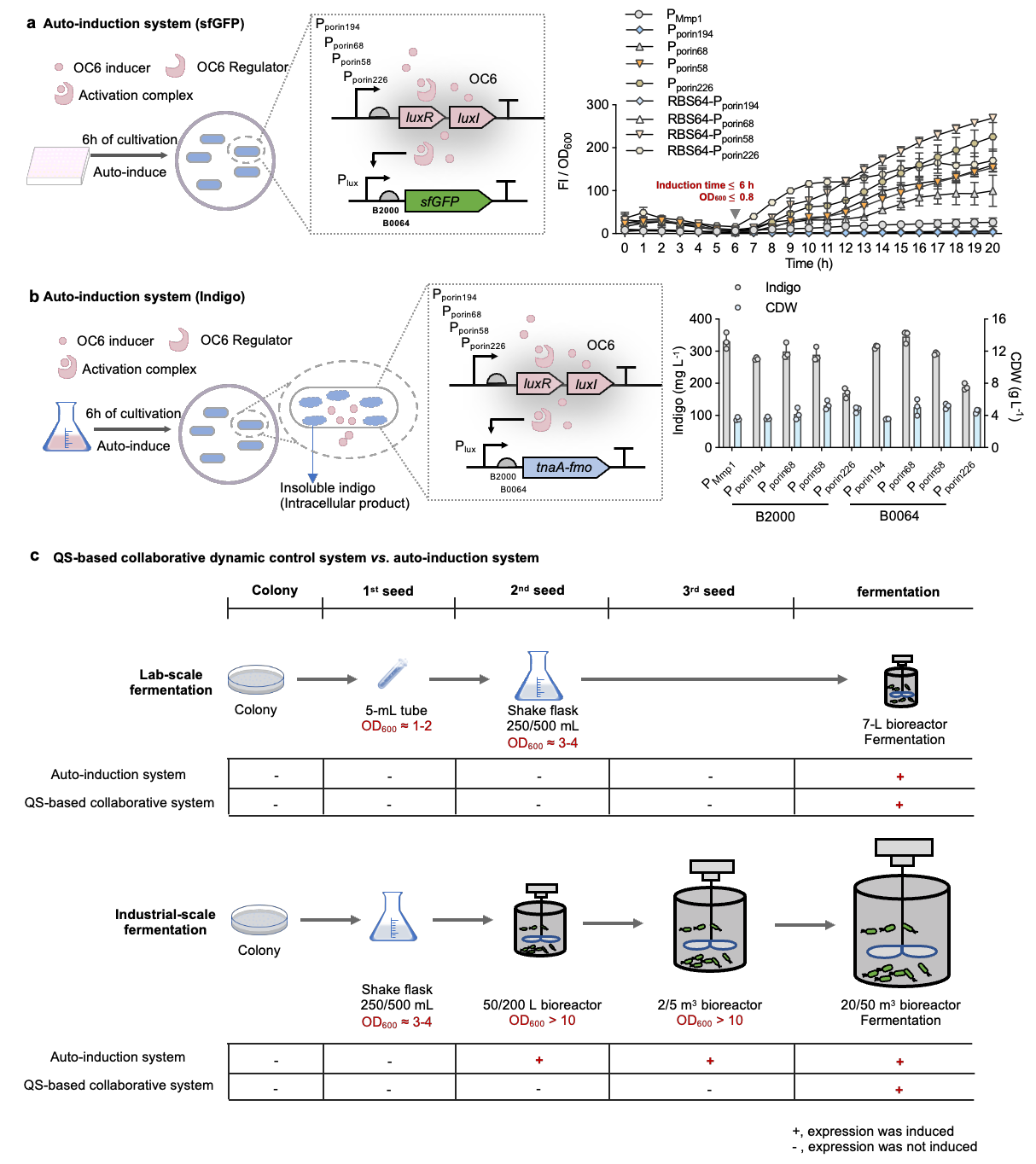
**

**Fig. S27 Comparative analysis of auto-induction and QS-based collaborative system in *Halomonas* TD.**

**a**, In contrast, an auto-induction system, consisting of *luxR*-*luxI* regulation panel (pSEVA321) and P_lux_-sfGFP control panel (pSEVA341), was designed for dynamic gene expression control. Specifically, promoters (P_porin194_, P_porin58_ and P_porin226_) and RBS (B2000 and B0064) with different expression strength were used to modulate the auto-induced performance (*luxR*-*luxI*) and expression output (P_lux_-sfGFP), respectively. Fluorescent intensity (FI/OD_600_) of different recombinant cells grown in a 96-well plate was on-line recorded by microplate reader in every hour for characterizing the auto-induction performance, including initially induced time point (≤ 6 h), OD_600_ (≤ 0.8), etc. **b**, Schema and shake flask study of indigo production from tryptophan by recombinant TD strain harboring *tnaA-fmo* module controlled by auto-induction system. **c**, Differences and challenges of auto-induction system during seed preparation process from lab-scale to industry-scale fermentation compared to QS-based collaborative dynamic control system developed in this study. The threshold of induced cell density for auto-induction system, which would be activated during seed preparation process conducted in large-size bioreactor ('+’ in red in bottom panel), is difficult to control and significantly lower than that by QS-based collaborative dynamic control. Data points in **a** to **b** are presented as mean ± SD from three independent biological replicates.

**References**

[1] R. Simon, U. Priefer, A. Pühler, *Bio/Technology* **1983**, *1* (9), 784.

[2] D. Tan, Y. S. Xue, G. Aibaidula, G. Q. Chen, *Bioresour Technol* **2011**, *102* (17), 8130.

[3] H. Zhao, H. M. Zhang, X. Chen, T. Li, Q. Wu, Q. Ouyang, G. Q. Chen, *Metab Eng* **2017**, *39*, 128.

[4] D. Tan, Q. Wu, J.-C. Chen, G.-Q. Chen, *Metab Eng* **2014**, *26*, 34.

[5] P. Ouyang, H. Wang, I. Hajnal, Q. Wu, Y. Guo, G. Q. Chen, *Metab Eng* **2018**, *45*, 20.

[6] Ji. K. Du, D. Yang, Z. W. Luo, S. Y. Lee, *Journal of Biotechnol* **2018**, 267, 19-28.

[7] W. Tao, L. Lv, G.-Q. Chen, *Microbial Cell Factories* **2017**, *16* (1), 48.

[8] X.-R. Jiang, Z.-H. Yao, G.-Q. Chen, *Metabolic Engineering* **2017**, *44*, 30.

[9] X. Z. Fu, D. Tan, G. A, Q. Wu, J. C. Chen, G. Q. Chen, *Metab Eng* **2014**, 23, 78-91.

[10] X. Wang, J. N. Han, X. Zhang, Y. Y. Ma, Y. Lin, H. Wang, D. J. Li, T. R. Zheng, F. Q. Wu, J. W. Ye, G. Q. Chen, *Nat Commun* **2021**, *12* (1), 1411.

[11] J. Zhou, M. Y. Li, Q. F. Chen, X. J. Li, L. F. Chen, Z. L. Dong, W. J. Zhu, Y. Yang, Z. L, Q. Chen, *Nat Commun* **2022**, 13, 3432.

[12] R. Silva-Rocha, E. Martinez-Garcia, B. Calles, M. Chavarria, A. Arce-Rodriguez, A. de Las Heras, A. D. Paez-Espino, G. Durante-Rodriguez, J. Kim, P. I. Nikel, R. Platero, V. de Lorenzo, *Nucleic Acids Res* **2013**, *41*, D666,.

[13] R. Shen, J. Yin, J. W. Ye, R. J. Xiang, Z. Y. Ning, W. Z. Huang, G. Q. Chen, *Acs Synth Biol* **2018**, *7* (8), 1897.

[14] Q. Qin, C. Ling, Y. Zhao, T. Yang, J. Yin, Y. Guo, G. Q. Chen, *Metab Eng* **2018**, *47*, 219.
